# Proteomic landscape of the primary somatosensory cortex upon sensory deprivation

**DOI:** 10.1093/gigascience/gix082

**Published:** 2017-08-23

**Authors:** Koen Kole, Rik G.H. Lindeboom, Marijke P.A. Baltissen, Pascal W.T.C. Jansen, Michiel Vermeulen, Paul Tiesinga, Tansu Celikel

**Affiliations:** 1 Department of Neurophysiology; 2 Department of Neuroinformatics, Donders Institute for Brain, Cognition, and Behaviour, Radboud University, Heyendaalseweg 135, 6525 HJ, Nijmegen, the Netherlands; 3 Department of Molecular Biology, Radboud Institute for Molecular Life Sciences, Radboud University, Geert Grooteplein 28, 6525 GA, Nijmegen, the Netherlands

**Keywords:** barrel cortex, whisker plucking, juvenile mice, mass spectrometry, label-free quantification, proteomics

## Abstract

Experience-dependent plasticity (EDP) powerfully shapes neural circuits by inducing long-lasting molecular changes in the brain. Molecular mechanisms of EDP have been traditionally studied by identifying single or small subsets of targets along the biochemical pathways that link synaptic receptors to nuclear processes. Recent technological advances in large-scale analysis of gene transcription and translation now allow systematic observation of thousands of molecules simultaneously. Here we employed label-free quantitative mass spectrometry to address experience-dependent changes in the proteome after sensory deprivation of the primary somatosensory cortex. Cortical column- and layer-specific tissue samples were collected from control animals, with all whiskers intact, and animals whose C-row whiskers were bilaterally plucked for 11–14 days. Thirty-three samples from cortical layers (L) 2/3 and L4 spanning across control, deprived, and first- and second-order spared columns yielded at least 10 000 peptides mapping to ∼5000 protein groups. Of these, 4676 were identified with high confidence, and >3000 were found in all samples. This comprehensive database provides a snapshot of the proteome after whisker deprivation, a protocol that has been widely used to unravel the synaptic, cellular, and network mechanisms of EDP. Complementing the recently made available transcriptome for identical experimental conditions (see the accompanying article by Kole et al.), the database can be used to (i) mine novel targets whose translation is modulated by sensory organ use, (ii) cross-validate experimental protocols from the same developmental time point, and (iii) statistically map the molecular pathways of cortical plasticity at a columnar and laminar resolution.

## Data Description

### Context

Sensory experience shapes neural circuits throughout life via experience-dependent plasticity (EDP). Changes in neural circuits, in turn, allow the brain to adapt to recent sensory, motor, and perceptual experiences of animals in their ever-changing environments.

The rodent barrel cortex, a subfield of the primary somatosensory cortex, processes sensory information originating from whiskers. Each cortical (barrel) column receives the majority of its sensory input from 1 (so-called principal) whisker, anatomically delineating the neural circuits associated with each whisker. Taking advantage of this organizational principle, previous studies have shown that targeted deprivation of select whiskers results in weakening of the sensory evoked responses in synaptic projections originating from barrel cortical layer (L)4 and targeting L2/3 in an experience-dependent manner [1, 2]. In contrast, corresponding projections in the neighbouring sparing whiskers’ cortical columns are strengthened [3]. The molecular mechanisms of EDP, however, are still largely unknown. Understanding how sensory experience shapes neuronal circuits will benefit from systematic analysis of the transcriptome and proteome following altered sensory experience. In an accompanying manuscript, we have provided a snapshot of the transcriptomic changes after 11–12 days of long sensory deprivation resolved across cortical columns and layers [4]. The database presented herein employs the same sensory deprivation protocol but focuses on the proteomic changes across cortical layers of L4 and L2/3 in columnar resolution.

### Methods

#### Animals

**Figure 1:**
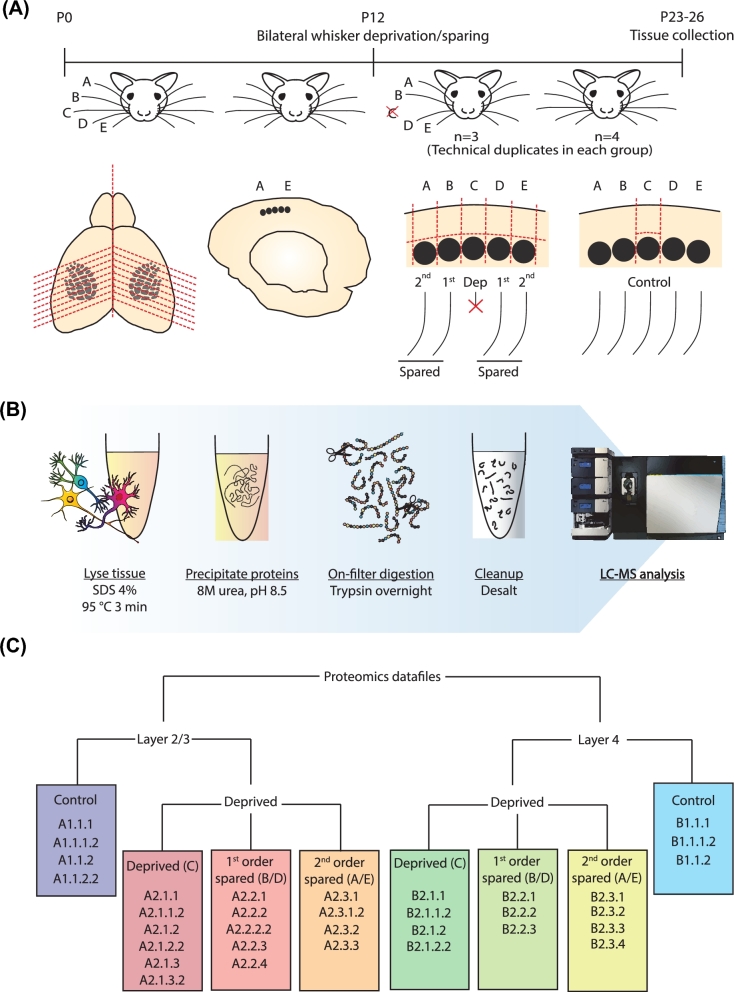
Overview of the experimental setup, sample collection, and data organization. (**A**) Pups were bilaterally spared or deprived of their C-row whiskers between P12 and P23-P26. Whisker deprivation, i.e., plucking, was repeated every third day to ensure that there was no regrowth of the whiskers. (**B**) Proteins were denatured and purified, followed by on-filter digestion into tryptic peptides, which were subsequently desalted on C18 StageTips and sequenced on a mass spectrometer. (**C**) Organization of data files in the database. Colours correspond to the colour code codes in Figs 2, 3, and 5, as well as the MS output file in the Supplementary Data. Sample codes of 5 digits (e.g., A2.1.1.2) indicate a technical replicate of the sample listed above it (e.g., A2.1.1). See Supplementary Table S1 for mapping of samples to mouse IDs.

All experiments were performed in accordance with National Institutes of Health Guidelines for the Care and Use of Laboratory Animals and were approved by the Animal Ethics Committee of the Radboud University in Nijmegen, the Netherlands. Pregnant wild-type mice (C57Bl6; Charles River, stock number 000664) (RRID:NCBITaxon_10090) were kept at a 12-hour light/dark cycle with access to food *ad libitum*. Cages were checked for birth daily. Experience-dependent plasticity was induced as described previously [4]. Briefly, at P12, C-row whiskers were plucked under isoflurane anaesthesia while control animals were not plucked but anaesthetized and handled similarly (Fig. 1A). Animals across groups were housed together with their mothers until tissue collection at P23-P26.

#### Slice preparation and sample collection

Tissue samples were collected from acute brain slices as described before [4]. In short, pups were deeply anaesthetized using isoflurane and perfused with ice-cold carbogenated slicing medium before 400-μm thalamocortical slices [1] were prepared. Slices were incubated in carbogenated aCSF at 37°C for 30 minutes before they were transferred to a holding chamber containing carbogenated aCSF at room temperature. Slices remained in this chamber until cortical layers and columns were isolated within ∼5–40 minutes.

For sample isolation, slices were placed under a microscope equipped with Dodt gradient contrast, used for visualization of the granular segments of the live neocortical tissue, such as the L4 in the barrel cortex. Visualized cortical columns (A-E) were separated from each other using a pulled pipette (Sutter Instruments P-2000), tip size of ∼5 micrometers, serving as a microneedle. Layers (L) 2/3 and L4 were isolated based on the established contrast criteria commonly used in electrophysiological analysis of barrel cortical neurons in acute slices [1, 2].

In the barrel cortex, cortical columns can be grouped by their relative distance to each other. Cortical columns B and D, for example, are named as the first-order neighboring cortical columns in respect to the C row column. Similarly, A and E row columns constitute the second-order neighboring columns. To increase the sample yield and have single animal resolution for the proteomic mapping, we pooled the samples within each layer across B and D, and A and E columns. Immediately after dissection, tissue samples were placed in Eppendorf tubes, snap-frozen in liquid nitrogen, and stored at –80°C until further use.

In the control group, tissues were collected from 3 separate mice (biological replicates) whereas the deprived group consisted of 4 animals. Only C-row layers were sampled in the control animals as the comparison across the C-rows between control and deprived animals allowed us to directly address the molecular changes associated with the whisker deprivation. Due to the small tissue sizes, obtaining successful liquid chromatography–mass spectrometry (LC-MS) runs was technically challenging. Thus, not all laminar samples from all cortical columns are retained for the full analysis (see Supplementary Table S1 for the distribution of samples across groups). In addition to these biological replicates, we ran 10 of the samples a second time, providing 10 technical replicates.

#### Lysate preparation and protein digestion

Samples were prepared for mass spectrometry using the filter-aided sample preparation (FASP) method, as described before (Fig. 1B) [5]. Briefly, mouse brain tissues were homogenized in lysis buffer (4% w/v SDS, 100 mM Tris/HCl and 0.1 M DTT, pH 7.6) and incubated at 95°C for 3 minutes. To shear DNA and reduce sample viscosity, samples were ultrasonicated. Samples were then clarified by centrifugation, after which the proteins in the extract were denatured using urea buffer (8M urea, 0.1 M Tris/HCl, pH 8.5) and centrifuge-filtered using 30 kDa filters (Microcon YM-30). After washing with urea buffer (pH 8.0), proteins were alkylated with iodoacetamide, followed by washing with ammonium bicarbonate. Trypsin (Promega Cat#V5280) was applied to digest the extracted proteins. The resulting peptides were then collected by centrifugation and desalted using C18 (Empore) StageTips. Given the small sample size, protein yield was not determined before moving on to mass spectrometry.

#### Mass spectrometry

Tryptic peptides were separated on an Easy-nLC 1000 (Thermo; RRID:SCR_014993) using a 214-minute-long gradient of acetonitrile (7–30%) followed by washes at 60%, followed by 95% acetonitrile for 240 minutes of total data collection. Mass spectra were collected on a LTQ-Orbitrap Fusion Tribrid mass spectrometer (Thermo; RRID:SCR_014992) in data-dependent top-speed mode with dynamic exclusion set at 60 seconds. Precursor MS spectra were acquired at an m/z range of 400–1500 at a resolution of 120.000 and a target value of 300 000 ions per full scan in the Orbitrap. MS/MS spectra were acquired in HCD mode using 35% collision energy, and fragmentation spectra were recorded in the ion trap.

#### Data processing

Raw data were analysed using MaxQuant (RRID:SCR_014485) version 1.5.1.0 with match-between-runs, label-free quantification, and intensity-based absolute quantification (iBAQ) enabled. Dependent peptides were enabled to perform an unbiased search against modifications on the identified peptides. The RefSeq protein sequence database downloaded on 28 June 2016 was used to identify proteins. Identified proteins were filtered for reverse hits and common contaminants. Contaminant proteins were determined by the MaxQuant software suite and include proteins that are often introduced during a typical mass spectrometry experiment such as keratins and trypsin. All other processing was performed in MATLAB (RRID:SCR_001622) or R (RRID:SCR_001905) programming languages.

#### Data validation and quality control

**Figure 2:**
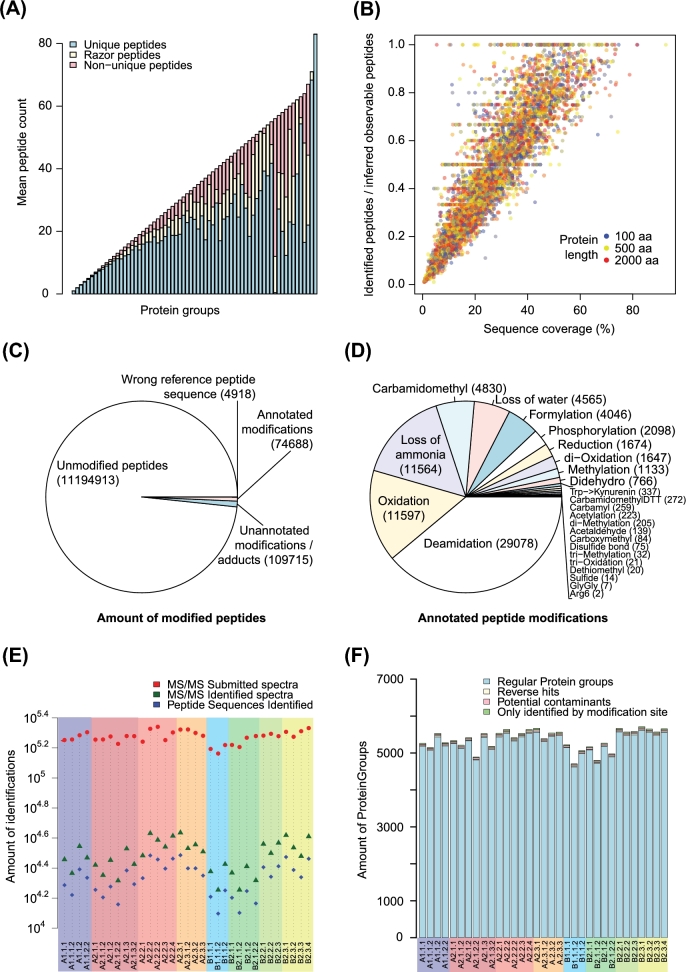
Overview of protein groups, sequence coverage, and peptide modifications. (**A**) Stack representation of designated protein groups with the mean contents of unique, razor, and non-unique peptides represented in blue, yellow, and red, respectively. (**B**) Sequence coverage of identified proteins was plotted as total protein sequence coverage against coverage of theoretically observable peptides (as determined by MaxQuant). (**C**) All identified peptides. (**D**) Identified peptide modifications with an annotated mass shift. (**E**) Submitted and identified MS spectra and uniquely identified amino acid sequences per sample. (**F**) Peptide and protein group identification confidence per sample. Colour coding corresponds to the experimental groups’ in Fig. 1C.

Peptides were assigned to protein groups based on shared peptide sequences, the majority of which consist mainly of unique peptide sequences (71%) (Fig. 2A). Razor peptides (i.e., peptides that can be assigned to more than 1 protein but are assigned to the protein group with the most other peptides, i.e., Occam's razor principle) on average made up 13% of the designated protein groups; non-unique peptides on average constituted 16%. When testing how much of the total and theoretically observable protein sequence length was identified by the analyses, we observed for most proteins a good coverage of the theoretically observable peptides (44% on average) (Fig. 2B). Complete sequence coverage is never achieved, likely because of the remaining tryptic peptides being too long or too short to be measured by mass spectrometry. Since high numbers of peptide modifications and adducts can interfere with accurate protein quantification, we assessed the types of peptide modifications that we could observe on the identified peptides (Fig. 2C and D). Peptide modifications may occur *in vivo* but more likely arise during the sample preparation steps. Reassuringly, the majority of peptides (98.33%) were found to be unmodified. For 0.96% of the peptides, we found a modified form with an unannotated mass shift, while 0.65% of peptides were modified and had a mass shift that could be annotated to a known peptide modification (Fig. 2C). In total, we could identify 25 different types of peptide modifications (Fig. 2D). Of these, the top 3 modifications were deamidation (38.94%), oxidation (15.53%), and loss of ammonia (15.48%), which are all common peptide modifications. Next, we addressed the data quality for individual samples, which showed that on average 23 489 unique amino acid sequences (ranging from 13 095 to 72 418) could be identified per sample (Fig. 2E); the majority of these (>98%) could be assigned to regular protein groups, excluding reverse hits, contaminants, or peptides identified only by modification. The reverse hit rate (i.e., false discovery rate) or the number of proteins that could only be identified based on a modified peptide was never higher than 0.7%, suggesting high confidence of protein identification. Additionally, the number of potential contaminants was low for all samples (minimum, 29; first quartile, 33; median, 35; mean, 34.52; third quartile, 36; maximum, 38), suggesting high sample purity (Fig. 2F).

**Figure 3:**
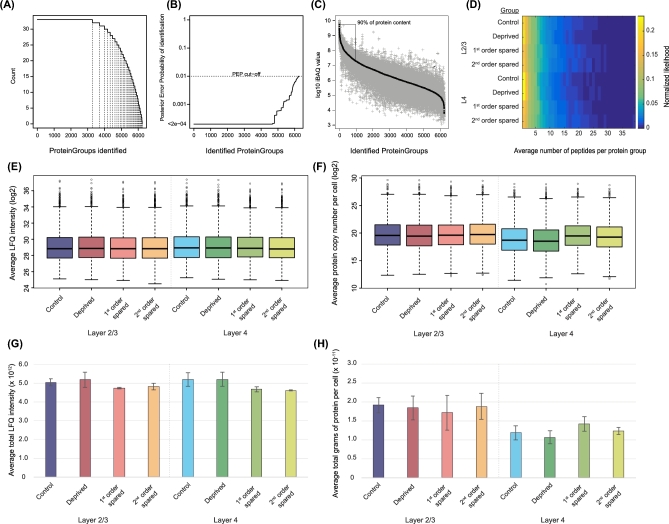
Quantification of protein groups across all samples. Control/deprived, C column; first-order spared, B/D columns; second-order spared, A/E columns (see Fig. 1). (**A**) Number of observations per protein group in the entire dataset. (**B**) Confidence of protein group identification across samples. (**C**) Protein content versus identified protein groups. For every protein group, all measured iBAQ values are plotted in grey, with the median value in black. (**D**) Averages and variances of peptides per protein group in each experimental group. (**E**) Box plot of LFQ intensity averages across samples within each group. (**F**) Box plot of protein copy numbers per cell (inferred as in [6]) averaged across samples within experimental groups. (**G**) Summed LFQ intensities averaged within experimental groups. (**H**) Total mass of identified proteins per cell, averaged within experimental groups. The inferred protein copy number per cell was divided by Avogrado's number (6.0221409 × 10^23^) and then multiplied by the protein mass in kilodaltons (kDa), yielding the total mass of identified proteins per cell.

Of the designated protein groups (i.e., protein groups with a posterior error probability [PEP; confidence of peptide identification] of <0.01, *n* = 6245), more than 3000 could be reliably identified in all of our samples (Fig. 3A and B); peptides in 4676 protein groups could be identified with high confidence (PEP < 0.0002). Of all identified proteins, 90% of the total protein content (as determined by intensity-based absolute quantification) [7] was contained in the 979 most abundant proteins (Fig. 3C). In this dataset, we identified and quantified proteins over 5 orders of magnitude, suggesting high sensitivity even at low protein concentrations.

To estimate the variance in protein quantification across samples, we averaged the number of identified peptides per protein group, which showed similar distributions across experimental groups (Fig. 3D). Additionally, we have performed 2 different normalizations: (i) averaging the LFQ intensity and copy number of each protein (as quantified according to the “proteomic ruler” approach [6], which uses the signal intensities of measured histones as an internal normalization) across samples within groups (Fig. 3E and F, respectively), and (ii) calculating the total LFQ intensity or protein mass across proteins within each sample and averaging across independent samples within a group (Fig. 3G and H, respectively). In the former, we included only those proteins that had a protein copy number of non-0. The results showed that, independent of the method of quantification, the experimental groups were similar to each other, suggesting that comparisons within protein groups between experimental groups should not be hampered by systematic differences in (inferred) protein abundances. Calculating the total mass of identified proteins per cell (by dividing inferred protein copy numbers per cell by Avogrado's number and multiplying by protein mass in kDa) showed that L2/3 cells on average contain 18.42 ±0.78 picograms of identified protein; this was 12.29 ±1.28 picograms in L4 cells (*P* = 0.0004, Student's *t* test) (Fig. 3H). The number of identified proteins averaged per group across layers did not differ (*P* = 0.6964, unpaired Student's *t* test). Since protein identification rates are likely to be independent from cortical layer identity, these results suggest that the total protein levels per cell are lower in L4. To investigate how the 2 quantification methods (i.e., LFQ and proteomic ruler approach) correspond, we examined the correlation between LFQ intensities and protein copy numbers (Supplemental Fig. S1). The correlation (*R*^2^) between the 2 quantification methods ranged from 0.76 to 0.80, suggesting good consensus of protein abundance estimation.

**Figure 4:**
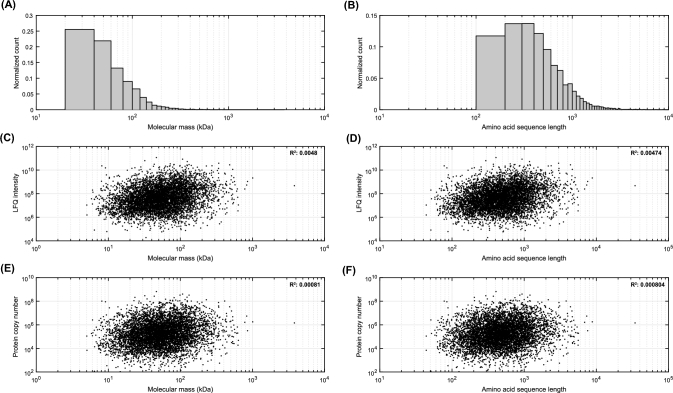
Protein quantification is independent from peptide mass or length. Distributions of (**A**) molecular mass and (**B**) amino acid sequence length; smaller and shorter proteins are the most prevalent. When plotting peptide mass or length against protein LFQ intensity (**C, D**) or protein copy number (inferred as in [6]) (**E, F**), weak (if any, see *R*^2^ values on figurines) correlations are observed, suggesting that protein abundance estimation is not biased by peptide mass or length (also see Fig. 2B).

We then assessed the distributions of molecular mass (kDa) and amino acid sequence length of the proteins identified in our samples. On average, proteins were 71.65 ±82.77 kDa in mass (Fig. 4A) and had a mean length of 643.63 ±745.27 amino acids (Fig. 4B). To exclude any bias in protein abundance estimation based on protein length, we plotted mass or sequence length against LFQ intensities or estimated protein copy number [5]. This showed only weak, if any, correlations (*R*^2^ values < ∼0.005) between LFQ intensity or copy number and peptide mass or length, suggesting that proteins of all sizes are equally well identified (Fig. 4C, D, E, F).

**Figure 5:**
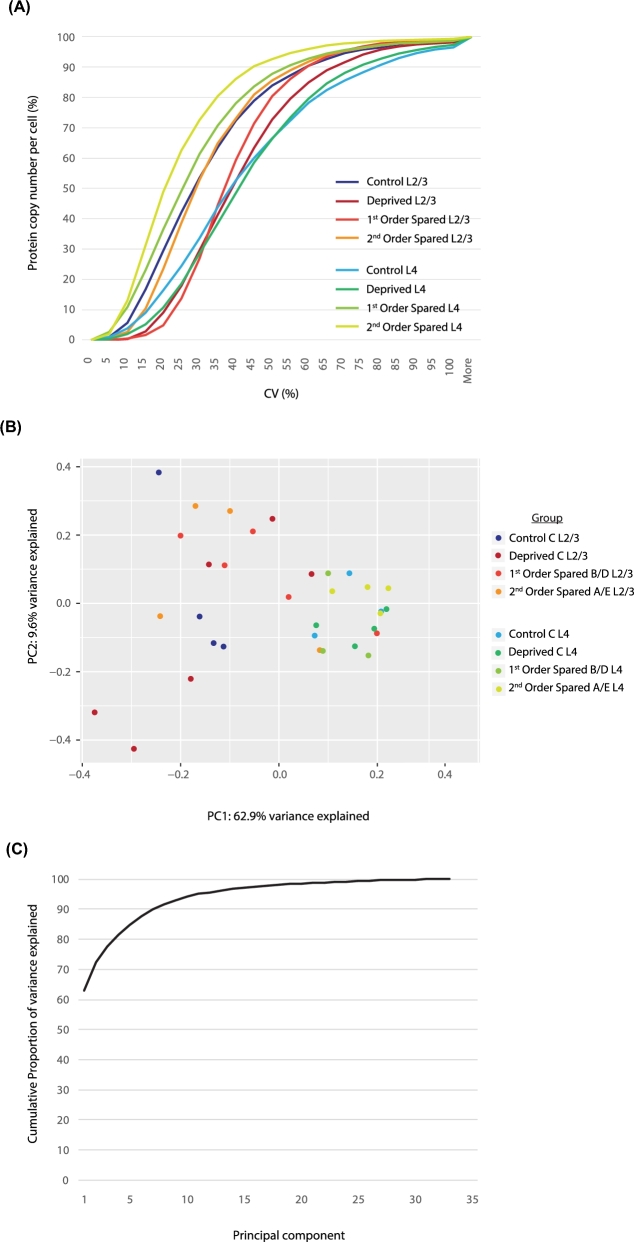
Variance quantification of individual samples. (**A**) Cumulative plot of the coefficient of variation of the inferred protein copy numbers [6] per cell and per experimental group. On average, ∼73% of proteins show a CV of 45% or less. (**B**) PCA based on inferred protein copy numbers per cell. PC 1 and 2 explain ∼73% of variance, and samples cluster mostly based on cortical laminar origin. (**C**) Cumulative plot of percent variance explained by each PC. The first 5 PCs explain 85% of the variance.

Next, we examined the variance between samples by calculating the coefficient of variation (CV) of inferred protein copy numbers (Fig. 5A) [6]. About 73% of proteins showed a CV of 45% or less on average. We then employed principal component analysis (PCA), which showed that 72.5% of variance was explained by PC1 and 2 and that samples were clustered mostly by cortical layer (Fig. 5B and C). These analyses were repeated for identified peptides for each protein group in individual samples, using different cut-offs of identified peptides (Fig. 3D). When no cut-off was used (i.e., including proteins identified by at least 1 peptide) (see Fig. 3D for the distribution across all groups), on average 73.88% of proteins showed a CV of 30% or less (Supplemental Fig. S2A); With a cut-off of 10 identified peptides, a CV of 15% or less was found for 70.74% of proteins (Supplemental Fig. S2B). PCA using both of these cut-offs showed that samples cluster mostly around C column–derived samples. Principal components (PCs) 1 and 2 explained 77.6% and 86.5% of variance, depending on the cut-off value used (Supplemental Fig. S2C–F).

**Figure 6:**
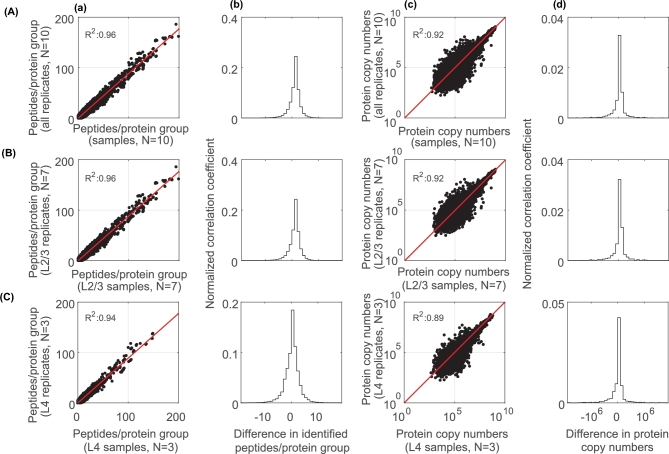
Matrix of correlation coefficients of biological and technical replicates. Data from (**A**) all biological samples and their corresponding replicates combined across experimental groups and cortical layers, (**B**) L2/3, and (**C**) L4. (**a, b**) Scatter plots showing peptides per protein group (a) or protein copy numbers (inferred copy numbers per cell (b)) [6] for biological samples (x-axis) and their technical replicates (y-axis). (**b, d**) Histograms showing differences in identified peptides per protein group (b) or protein copy numbers (**d**) between biological and technical replicates. Note that across all samples, the variations between the biological sample and the technical replicas are small, with Pearson *R*^2^ values between 0.89–96.

Since our dataset contains several technical duplicates, we asked how well they correlate with the biological replicates and compared identified peptides per protein group and protein copy numbers of biological and technical replicates (Fig. 6). Biological samples and their direct technical replicates were highly correlated (*R*^2^ ≥ 0.89) (Fig. 6A–C, a and c), which was also found for the remaining pairwise comparisons (*R*^2^ ≥ 0.90) (Supplemental Figs S3 and S4). These results suggest that samples are highly comparable in terms of peptide and protein counts and that the sequential nature of mass spectroscopy does not systematically, or in statistically appreciably fashion, bias protein quantifications, at least in our samples.

### Re-use potential

The current dataset provides a proteomics view of the experience-dependent plasticity in the mouse barrel cortex. Since barrel cortex is a popular model system where sensory processing and experience-dependent plasticity are studied from molecules to behavior (e.g., [1–3, 8, 10, 12, 13]), this resource should help to identify some of the molecular underpinnings of cortical plasticity. Given the relatively high anatomical resolution at which samples were collected, the current dataset would also be beneficial in the understanding of molecular constituents of cortical laminar identity and function. It should be noted, however, that the collected samples contain the entirety of the cellular population, i.e., are not cell type–specific. Signals originating from all cell types are thus averaged, which should be considered by researchers reusing this dataset. The cellular complexity of the samples studied herein will be particularly useful for those efforts aiming to identify the neuronal as well as the non-neuronal basis of experience-dependent plasticity.

A combinatorial approach between proteomics and transcriptomics (e.g., RNA sequencing) [4] is a promising outlook that could help to identify those molecular targets that are essential for reorganization of neural networks following sensory deprivation. Proteomics data can help broaden the scope of findings from transcriptomics studies as they can provide novel insights into post-transcriptional regulation of protein expression, the time course of protein expression (since proteins typically have a longer half-life than RNAs), and post-translational modifications that could orchestrate specific protein functions.

Only a few studies are available that focus on large-scale molecular changes in neural circuits following sensory deprivation [9–10]. As large-scale molecular techniques are becoming more accessible, studies employing them to investigate the molecular bases of plasticity are likely to follow suit. The phenotype of EDP in the barrel cortex depends heavily on the experimental approach used (e.g., enrichment vs deprivation, single whisker experience vs whole row deprivation, developmental time points) [12, 13]. The current dataset should prove useful to validate, expand, and compare the findings of molecular studies employing different protocols. Moreover, comparing our dataset with those obtained from other brain regions (e.g., visual cortex, auditory cortex) would help to determine where previously observed differences in plasticity across different brain [13] regions might arise.

## Availability of the supporting data

Data supporting this work are available in the *GigaScience* respository, *Giga*DB [14]. The raw mass spectrometry proteomics data have been deposited in the ProteomeXchange Consortium via the PRIDE partner repository [15] with the dataset identifier PXD005971.

## Additional files

Supplemental Figure S1. Correlation between LFQ and protein copy numbers. Scatter plots of LFQ values (x-axis) and inferred protein copy numbers [6] (y-axis), showing a linear correlation between the 2 quantification methods (*R*^2^ > 0.75).

Supplemental Figure S2. Variance quantification of individual samples. (A, B) Cumulative plots of the coefficient of variance in the number of identified peptides in each experimental group. Including proteins with at least 1 identified peptide (A), CVs of 30% or less are found in ∼74%. With an increased cut-off (10 peptides), ∼70% of proteins show a CV of 15% or less (C). (C, D) Principal component analysis using numbers of identified peptides per protein. With a cut-off of 1 identified peptide, ∼78% of variance is explained by PC 1 and 2 (B); this is ∼87% when a cut-off of 10 identified peptides is used (D). (E, F) Cumulative plots of showing the percent variance explained by each PC. With a cut-off of 1 identified peptide (C), the first 5 PCs explain ∼83% of the variance; using a cut-off of 10 peptides (F), this is ∼91%.

Supplemental Figure S3. Distribution of peptides per protein group in biological and technical replicates. Scatter plots of identified peptides per protein group from biological and technical replicates (see Fig. 1C for coding). Red-bordered graphs indicate pairwise comparisons between biological samples and their direct technical replicate; graphs with black borders contain the remaining comparisons. Overall, a strong linear correlation is observed in pairwise comparisons (*R*^2^ = 0.95 ±0.01), in particular between biological and technical replicate pairs (*R*^2^ ≥ 0.96 ±0.01). Scale bars correspond to 100 peptides per protein group.

Supplemental Figure S4. Copy number distribution of biological and technical replicates. Log-log plots show protein copy numbers from biological and technical replicates. Pairwise comparisons between biological samples and their direct technical replicate are indicated by red borders; black borders indicate the remaining comparisons. As in Supplemental Fig. S2, pairwise comparisons show high correlations between individual samples (average *R*^2^ = 0.90 ±0.01), which are highest for biological and technical replicate pairs (*R*^2^ = 0.93 ±0.03).

Supplemental Table S1. Origin and distribution of samples. Colours correspond to those in Fig. 1C. Samples that were run once are marked X, technically duplicated samples are marked XX.

Supplemental Table S2. R commands for PCA analysis and plots.

## Abbreviations

EDP: experience-dependent plasticity; LC-MS: liquid chromatographymass spectrometry; L2/3: cortical layer 2/3, also known as supragranular layers; L4: cortical layer 4, i.e., granular layer.

## Competing interests

The authors declare no competing interests.

## Funding

The current work was funded by the Faculty of Science of the Radboud University, Nijmegen, the Netherlands (grant number 626830–6200821), as well as the ALW Open Programme of the Netherlands Organization for Scientific Research (NWO; grant number 824.14.022).

## Author contributions

K.K. performed all experimental manipulations and sample acquisition, prepared the tables and figures, and performed bioinformatic analysis. R.L. performed bioinformatic analysis and prepared the tables and figures. P.J. and M.B. performed sample preparation and mass spectrometry. M.V. supervised the proteomics pipeline. P.T. co-supervised the project. T.C. designed and supervised the project and performed bioinformatic analysis. K.K. and T.C. wrote the manuscript. All authors edited and approved the final version of the manuscript.

## Supplementary Material

GIGA-D-17-00057_Original-Submission.pdf

GIGA-D-17-00057_Revision-1.pdf

Response-to-Reviewer-Comments_Original-Submission.pdf

Reviewer-1-Report-(Original-Submission).pdf

Reviewer-2-Report-(Original-Submission).pdf

Supplement materials
