# Erratum to: Proteomic landscape of the primary somatosensory cortex upon sensory deprivation

**DOI:** 10.1093/gigascience/giy041

**Published:** 2018-07-20

**Authors:** Koen Kole, Rik G H Lindeboom, Marijke P A Baltissen, Pascal W T C Jansen, Michiel Vermeulen, Paul Tiesinga, Tansu Celikel

In the final publication of “Proteomic landscape of the primary somatosensory cortex upon sensory deprivation,” by Koen Kole et al., the publication month for reference 4 was incorrect. The publication month for reference 4 has been corrected and the corrected manuscript is published in *GigaScience*, Volume 6, Issue 10: https://doi.org/10.1093/gigascience/gix082.
