# Supplementary material for: Proteomic landscape of the primary somatosensory cortex upon sensory deprivation
Source: Gigascience. 2017 Aug 23;6(10):1–10. doi: 10.1093/gigascience/gix082 (PMC5632293; doi:10.1093/gigascience/gix082)
Supplement: GIGA-D-17-00057_Original-Submission.pdf [file gix082_GIGA-D-17-00057_Original-Submission.pdf]

Manuscript
Click here to download Manuscript Kole_Lindeboom_Gigascience.pdf

Proteomic landscape of the primary somatosensory cortex upon sensory deprivation

Koen Kole[1,2]*, Rik G.H. Lindeboom[3]*, Marijke P.A. Baltissen[3], Pascal W.T.C. Jansen[3], Michiel Vermeulen[3], Paul Tiesinga[2], Tansu Celikel[1]

(1) Department of Neurophysiology, (2) Department of Neuroinformatics, Donders Institute for Brain, Cognition, and Behaviour, Radboud University, Nijmegen - the Netherlands. (3) Department of Molecular Biology, Radboud Institute for Molecular Life Sciences, Radboud University, Nijmegen - the Netherlands

* indicates equal contribution

E-mail addresses (in the order of appearance): k.kole@neurophysiology.nl, r.lindeboom@science.ru.nl, m.baltissen@science.ru.nl, p.jansen@science.ru.nl, michiel.vermeulen@science.ru.nl, p.tiesinga@science.ru.nl, celikel@neurophysiology.nl (corresponding author)

Keywords (3-10): Barrel cortex, whisker plucking, juvenile mice, mass-spectrometry, label-free quantification, proteomics

Abstract (word count: 244)

Background: Experience-dependent plasticity (EDP) powerfully shapes neural circuits by inducing long-lasting molecular changes in the brain. Molecular mechanisms of EDP have been traditionally studied by identifying single or small subsets of targets along the biochemical pathways that link synaptic receptors to nuclear processes. Recent technological advances in large-scale analysis of gene transcription and translation now allow systematic observation of thousands of molecules simultaneously. Here we employed label-free quantitative mass spectrometry to address experience-dependent changes in the proteome after sensory deprivation of the primary somatosensory cortex.

Findings: Cortical column- and layer-specific tissue samples were collected from control animals, with all whiskers intact, and animals whose C-row whiskers were bilaterally plucked for 11-14 days. 33 samples from cortical layers (L) 2/3 and L4 spanning across control, deprived, 1st and 2nd order spared columns yielded at least 10,000 peptides mapping to ~5000 protein groups. Of these, 4,676 were identified with high confidence and >3000 are found in all samples.

Conclusions: This comprehensive database provides a snapshot of the proteome after whisker deprivation, a protocol that has been widely used to unravel the synaptic, cellular and network mechanisms of EDP. Complementing the recently made available transcriptome for identical experimental conditions (Kole et al, submitted), the database can be used to (1) mine novel targets whose translation is modulated by sensory organ use, (2) cross-validate experimental protocols from the same developmental time point, and (3) statistically map the molecular pathways of cortical plasticity at a columnar and laminar resolution.

Data Description

Context

Sensory experience shapes neural circuits throughout life via experience-dependent plasticity (EDP). Changes in neural circuits, in turn, allow the brain to adapt to recent sensory, motor and perceptual experiences of animals in their ever-changing environments.

The rodent barrel cortex, a subfield of the primary somatosensory cortex, processes sensory information originating from whiskers. Each cortical (barrel) column receives majority of its sensory input from one (so called principal) whisker, anatomically delineating the neural circuits associated with each whisker. Taking advantage of this organizational principle, previous studies have shown that targeted deprivation of select whiskers result in weakening of the sensory evoked responses in synaptic projections originating from barrel cortical layer (L)4 and targeting L2/3 in an experience-dependent manner [1, 2]. In contrast, corresponding projections in the neighbouring sparing whiskers' cortical columns are strengthened [3]. The molecular mechanisms of EDP, however, are still largely unknown. Understanding how sensory experience shapes neuronal circuits will benefit from systematic analysis of the transcriptome and proteome following altered sensory experience. In an accompanying manuscript we have provided a snapshot of the transcriptomic changes after 11-12 days long sensory deprivation resolved across cortical columns and layers (Kole et al, submitted). The database presented herein employs the same sensory deprivation protocol but focuses on the proteomic changes across cortical layers of L4 and L2/3 in columnar resolution.

Methods

Animals: All experiments were performed in accordance with NIH Guidelines for the Care and Use of Laboratory Animals, and were approved by the Animal Ethics Committee of the Radboud

University in Nijmegen, the Netherlands. Pregnant wild type mice (C57Bl6; Charles River) were kept at a 12 hour light/dark cycle with access to food *ad libitum*. Cages were checked for birth daily. Experience-dependent plasticity was induced as described previously (Kole et al, submitted). Briefly, at P12, C-row whiskers were plucked under isoflurane anaesthesia while control animals were not plucked but anaesthetized and handled similarly (**Figure 1A**). Animals across groups were housed together with their mothers until tissue collection at P23-P26. The deprived and control groups consisted of 3 female pups each, corresponding to 3 independent biological samples (i.e. replicates) in each experimental group. In addition 10 of the samples were ran a second time, providing 10 technical replicates (see Figure 1 for the distribution of samples across groups).

[Figure 1 is about here]

Slice preparation and sample collection: Tissue samples were collected from acute brain slices as described before (Kole et al, submitted). In short, pups were deeply anaesthetized using isoflurane and perfused with ice-cold carbogenated slicing medium before 400 µm thalamocortical slices [1] were prepared. After a 30 minute recovery in aCSF, L4 and L2/3 were isolated from barrel cortical columns A-E under a microscope using a pulled patch pipette; tissue from columns A and B were pooled with tissue from columns E and D, respectively. Immediately after dissection, tissue samples were snap frozen in liquid nitrogen and stored at -80ºC until further use.

Lysate preparation and protein digestion: Samples were prepared for mass spectrometry using the filter-aided sample preparation (FASP) method, as described before [4] (**Figure 1B**). Briefly, mouse brain tissues were homogenized in lysis buffer (4% w/v SDS, 100 mM Tris/HCl and 0.1 M DTT, pH 7.6) and incubated at 95 °C for 3 min. To shear DNA and reduce sample viscosity, samples were ultrasonicated. Samples were then clarified by centrifugation, after

which the proteins in the extract were precipitated using urea buffer (8M urea, 0.1 M Tris/HCl, pH 8.5) and centrifuge-filtered using 30 kDa filters (Microcon YM-30). After washing with urea buffer (pH 8.0), proteins were alkylated with iodoacetamide, followed by washing with ammonium bicarbonate. Trypsin (Promega Cat#V5280) was applied to digest the extracted proteins. The resulting peptides were then collected by centrifugation and desalted using C18 (Empore) StageTips. Given the small sample size protein yield was not determined before moving on to mass spectrometry.

Mass spectrometry: Tryptic peptides were separated on an online Easy-nLC 1000 (Thermo) using a 214 minute long gradient of acetonitrile (7% to 30%) followed by washes at 60%, followed by 95% acetonitrile for 240 min of total data collection. Mass spectra were collected on a LTQ-Orbitrap Fusion Tribrid mass spectrometer (Thermo) in data-dependent top-speed mode with dynamic exclusion set at 60 s.

Data processing: Raw data was analysed using MaxQuant version 1.5.1.0. with match-between-runs, label-free quantification and intensity based absolute quantification (iBAQ) enabled. Dependent peptides were enabled to perform an unbiased search against modifications on the identified peptides. The RefSeq protein sequence database downloaded on 28-06-2016 was used to identify proteins. Identified proteins were filtered for reverse hits and common contaminants. To quantify protein expression in Figure 4C, we used the Proteomic Ruler approach [5]. All other processing was performed in MATLAB or R programming languages.

Data validation and quality control

Peptides were assigned to protein groups based on shared peptide sequences, the majority of which consist mainly of unique peptide sequences (71%, **Figure 2A**). Razor peptides (i.e.

peptides that can be assigned to more than one protein but are assigned to the protein group with the most other peptides, i.e. Occam's razor principle) on average made up 13% of the designated protein groups; non-unique peptides on average constituted 16%. When testing how much of the total and theoretically observable protein sequence length was identified by the analyses, we observe for most proteins a good coverage of the theoretically observable peptides (44% on average, **Figure 2B**). Complete sequence coverage is never achieved, likely because of the remaining tryptic peptides being too long or too short to be measured by mass spectrometry. Since high numbers of peptide modifications and adducts can interfere with accurate protein quantification, we assessed the types of peptide modifications that we could observe on the identified peptides (**Figure 2C** and **2D**). Reassuringly, the majority of peptides (98.33%) were found to be unmodified. For 0.96% of the peptides we found a modified form with an unannotated mass shift, while 0.65% of peptides was modified and had a mass shift that could be annotated to a known peptide modification (**Figure 2C**). In total we could identify 25 different types of peptide modifications (**Figure 2D**). Of these, the top three modifications were deamidation (38.94%), oxidation (15.53%) and loss of ammonia (15.48%), which are all common peptide modifications. Next, we addressed the data quality for individual samples, which showed that on average 23,489 unique amino acid sequences (ranging from 13,095 to 72,418) could be identified per sample (**Figure 2E**); the majority of these (>98%) could be assigned to regular protein groups, excluding reverse hits, contaminants or peptides identified only by modification. Reverse hit rate (i.e. false discovery rate) or the number of proteins that could only be identified based on a modified peptide was never higher than 0.7%, suggesting high confidence of protein identification. Additionally, the number of potential contaminants was low for all samples, suggesting high sample purity (**Figure 2F**).

[Figure 2 is about here]

Of the designated protein groups (i.e. protein groups with a Posterior Error Probability (PEP,

confidence of peptide identification) of <0.01, n = 6,245), over 3,000 could be reliably identified in all of our samples (**Figure 3A** and **3B**); peptides in 4,676 protein groups could be identified with high confidence (PEP <0.0002). Of all identified proteins, 90% of the total protein content (as determined by intensity based absolute quantification [6]) was contained in the 979 most abundant proteins (**Figure 3C**). In this dataset we identified and quantified proteins over five orders of magnitude, suggesting high sensitivity even at low protein concentrations.

[Figure 3 is about here]

To estimate the variance in protein quantification across samples, we averaged the number of identified peptides per protein group, which showed similar distributions across experimental groups (**Figure 3D**). Additionally we have performed two different normalizations: 1) Averaging the LFQ intensity and copy number of each protein (as quantified according to the "proteomic ruler" approach [5], which uses the signal intensities of measured histones as an internal normalization) across samples (**Figure 3E,F**, respectively), and 2) calculating the total LFQ intensity or protein mass across proteins within each sample and averaging across independent samples within a group (**Figure 3G,H**, respectively). In the former, we included only those proteins that had a protein copy number of non-zero. The results showed that independent of the method of quantification the experimental groups were similar to each other, suggesting that comparisons within protein groups between experimental groups should not be hampered by systematic differences in (inferred) protein abundances. Calculating the total mass of identified proteins per cell (by dividing inferred protein copy numbers per cell by Avogrado's number and multiplying by protein mass in kDa) showed that L2/3 cells on average contain 18.42 ±0.78 picograms of identified protein; this was 12.29 ±1.28 picograms in L4 cells ($p = 0.0004$, Student's t-test) (**Figure 3H**). The number of identified proteins averaged per group across layers did not differ ($p=0.6964$, unpaired Student's t-test). Since protein identification rates are likely to be independent from cortical layer identity, these results suggests that total protein

levels per cell are lower in L4. To investigate how the two quantification methods (i.e. LFQ and proteomic ruler approach) correspond, we examined the correlation between LFQ intensities and protein copy numbers (**Supplemental Figure 1**). The two quantification methods were decently correlated (R^2 ranged from 0.76 to 0.80), suggesting good consensus of protein abundance estimation.

We then assessed the distributions of molecular mass (kDa) and amino acid sequence length of the proteins identified in our samples. On average, proteins were 71.65 ±82.77 kDa in mass (mean) (**Figure 4A**) and had a mean length of 643.63 ±745.27 amino acids (**Figure 4B**). To exclude any bias in protein abundance estimation based on protein length, we plotted mass or sequence length against LFQ intensities or estimated protein copy number [5]. This showed only weak, if any, correlations (R^2 values <~0.005) between LFQ intensity or copy number and peptide mass or length, suggesting proteins of all sizes are equally well identified (**Figure 4C, D, E, F**).

[Figure 4 is about here]

Next, we examined the variance between samples by calculating the coefficient of variation (CV) of identified peptides for each protein group in individual samples, using different cut-offs of identified peptides (**Figure 3D**). When no cut-off was used (i.e. including proteins identified by at least one peptide, see Figure 3D for the distribution across all groups), on average 73.88% of proteins showed a CV of 30% or less (**Figure 5A**); With a cut-off of 10 identified peptides, a CV of 15% or less was found for 70.74% of proteins (**Figure 5C**). We then employed principal component analysis (PCA) using both of these cut-offs, which showed that samples cluster mostly around C column-derived samples. Principal components (PC) 1 and 2 explained 55% and 76% of variance, depending on the cut-off value used (**Figure 5B, Figure 5D**). These

analyses were repeated based on inferred protein copy numbers (including only non-zero values) (**Figure 5E, F**). About 73% of proteins showed a CV of 45% or less, on average. In the PCA, ~73% of variance was explained by PC1 and 2, and samples were clustered mostly by cortical layer.

[Figure 5 is about here]

Since our dataset contains several technical duplicates, we asked how well they correlate with the biological replicates and compared identified peptides per protein group and protein copy numbers of biological and technical replicates (**Figure 6**). Biological samples and their direct technical replicates were highly correlated ($R^2 \geq 0.89$, **Figure 6A-Ca,c**), which was also found for the remaining pairwise comparisons ($R^2 \geq 0.90$) (**Supplemental Figure 2, 3**). These results suggest that samples are highly comparable in terms of peptide and protein counts, and that sequential nature of the mass spectroscopy does not systematically, or in statistically appreciably fashion, bias protein quantifications, at least in our samples.

[Figure 6 is about here]

Re-use potential

The current dataset provides a proteomics view of the experience-dependent plasticity in the mouse barrel cortex. Since barrel cortex is heavily studied in the field of neuroscience [e.g. 1-3,8-10,12,13], especially in relation to plasticity, this resource should prove a valuable for researchers interested in the molecular and cellular underpinnings of EDP. Given the relatively high anatomical resolution at which samples were collected, the current dataset would also be beneficial in the understanding of molecular constituents of cortical laminar identity and function.

A combinatorial approach between proteomics and transcriptomics (e.g. RNA sequencing; Kole et al., submitted) is a promising outlook that could help identifying those molecular targets that

are essential for reorganization of neural networks following sensory deprivation. Proteomics data can aid to broaden the scope of findings from transcriptomics studies as it can provide novel insights into post-transcriptional regulation of protein expression, the time course of protein expression (since proteins typically have a longer half-life than RNAs) and post-translational modifications that could orchestrate specific protein functions.

Only a few studies are available that focus on large-scale molecular changes in neural circuits following sensory deprivation [9–11]. As large-scale molecular techniques are becoming more available, studies employing them to investigate the molecular bases of plasticity are likely to follow suit. The phenotype of EDP in barrel cortex depends heavily on the experimental approach used (e.g. enrichment vs. deprivation, single whisker experience vs. whole row deprivation, developmental time points [12]). The current dataset should prove useful to validate, expand and compare the findings of molecular studies employing different protocols. Moreover, comparing our dataset with those obtained from other brain regions (e.g. visual cortex, auditory cortex), would help to determine where previously observed differences in plasticity across different brain [13] regions might arise.

References

1. Allen CB, Celikel T, Feldman DE (2003) Long-term depression induced by sensory deprivation during cortical map plasticity in vivo. Nat Neurosci 6:291–9. doi: 10.1038/nn1012

2. Celikel T, Szostak VA, Feldman DE (2004) Modulation of spike timing by sensory deprivation during induction of cortical map plasticity. Nat Neurosci 7:534–41. doi: 10.1038/nn1222

3. Clem RL, Celikel T, Barth AL (2008) Ongoing in vivo experience triggers synaptic metaplasticity in the neocortex. Science 319:101–4. doi: 10.1126/science.1143808

4. Wiśniewski JR, Zougman A, Nagaraj N, Mann M (2009) Universal sample preparation method for proteome analysis. Nat Methods 6:359–362. doi: 10.1038/nmeth.1322

5. Wiśniewski JR, Hein MY, Cox J, Mann M (2014) A "Proteomic Ruler" for Protein Copy Number and Concentration Estimation without Spike-in Standards. Mol Cell Proteomics 13:3497–3506. doi: 10.1074/mcp.M113.037309

6. Schwanhäusser B, Busse D, Li N, Dittmar G, Schuchhardt J, Wolf J, Chen W, Selbach M (2011) Global quantification of mammalian gene expression control. Nature 473:337–342. doi: 10.1038/nature10098

7. Molyneaux BJ, Arlotta P, Menezes JRL, Macklis JD (2007) Neuronal subtype specification in the cerebral cortex. Nat Rev Neurosci 8:427–37. doi: 10.1038/nrn2151

8. Zeisel A, Munoz-Manchado AB, Codeluppi S, Lonnerberg P, La Manno G, Jureus A, Marques S, Munguba H, He L, Betsholtz C, Rolny C, Castelo-Branco G, Hjerling-Leffler J, Linnarsson S (2015) Cell types in the mouse cortex and hippocampus revealed by single-cell RNA-seq. Science (80-) 347:1138–1142. doi: 10.1126/science.aaa1934

9. Butko MT, Savas JN, Friedman B, Delahunty C, Ebner F, Yates JR, Tsien RY (2013) In vivo quantitative proteomics of somatosensory cortical synapses shows which protein levels are modulated by sensory deprivation. Proc Natl Acad Sci U S A 110:E726-35. doi: 10.1073/pnas.1300424110/-/DCSupplemental.www.pnas.org/cgi/doi/10.1073/pnas.1300424110

10. Vallès A, Boender AJ, Gijsbers S, Haast R a M, Martens GJM, de Weerd P (2011) Genomewide analysis of rat barrel cortex reveals time- and layer-specific mRNA expression changes related to experience-dependent plasticity. J Neurosci 31:6140–58. doi: 10.1523/JNEUROSCI.6514-10.2011

11. Fernández-Montoya J, Buendia I, Martin YB, Egea J, Negredo P, Avendaño C (2016) Sensory Input-Dependent Changes in Glutamatergic Neurotransmission- Related Genes and Proteins in the Adult Rat Trigeminal Ganglion. Front Mol Neurosci 9:132. doi: 10.3389/fnmol.2016.00132

12. Feldman DE, Brecht M (2005) Map plasticity in somatosensory cortex. Science 310:810–5. doi: 10.1126/science.1115807

13. Fox K, Wong ROL (2005) A comparison of experience-dependent plasticity in the visual and somatosensory systems. Neuron 48:465–77. doi: 10.1016/j.neuron.2005.10.013

14. Vizcaíno JA, Csordas A, del-Toro N, Dianes JA, Griss J, Lavidas I, Mayer G, Perez-Riverol Y, Reisinger F, Ternent T, Xu QW, Wang R, Hermjakob H. (2016) 2016 update of the PRIDE database and related tools. Nucleic Acids Res. 1;44(D1): D447-D456. doi: 10.1093/nar/gkv1145

Availability of the supporting data

Supporting data are available online (https://goo.gl/0lrdCg) and will be distributed via GigaScience DB. The raw mass spectrometry proteomics data have been deposited to the ProteomeXchange Consortium via the PRIDE partner repository [14] with the dataset identifier PXD005971.

List of abbreviations

EDP Experience dependent plasticity

L2/3 Cortical Layer 2/3, also known as supragranular layers

L4 Cortical Layer 4, i.e. granular layer

Competing interests

The authors declare no competing interests.

Author contributions

KK performed all experimental manipulations, sample acquisition and prepared the tables and figures, performed bioinformatic analysis. RL performed bioinformatic analysis and prepared the tables and figures. PJ and MB performed sample preparation and mass spectrometry. MV supervised the proteomics pipeline. PT co-supervised the project. TC designed and supervised the project, performed bioinformatic analysis. KK and TC wrote the manuscript. All authors edited otherwise approved the final version of the manuscript.

Figure legends

Figure 1. Overview of the experimental setup, sample collection and data organization. (A) Pups were bilaterally spared or deprived of their C-row whiskers between P12 and P23-P26. Whisker deprivation, i.e. plucking, was repeated every third day to ensure that there was no regrowth of the whiskers. **(B)** Proteins were denatured and purified, followed by on-filter digestion into tryptic peptides, which were subsequently desalted on C18 StageTips and sequenced on a mass spectrometer. **(C)** Organization of data files in the database. Colours correspond to the colour code codes in Figures 2,3, and 5, as well as the MS output file among the Supplemental Data. Sample codes of 5 digits (e.g. A2.1.1.2) indicate a technical replicate of the sample listed above it (e.g. A2.1.1)

Figure 2. Overview of protein groups, sequence coverage and peptide modifications. (A) Stack representation of designated protein groups with the mean contents of unique, razor and non-unique peptides represented in blue, yellow and red, respectively. **(B)** Sequence coverage of identified proteins was plotted as total protein sequence coverage against coverage of theoretically observable peptides (as determined by MaxQuant). **(C)** All identified peptides. **(D)** Identified peptide modifications with an annotated mass shift. **(E)** Submitted and identified MS spectra and uniquely identified amino acid sequences per sample. **(F)** Peptide and protein group identification confidence per sample. Colour coding corresponds to the experimental groups' in Figure 1C.

Figure 3. Quantification of protein groups across all samples. (A) Number of observations per protein group in the entire dataset. **(B)** Confidence of protein group identification across

samples. **(C)** Protein content versus identified protein groups. For every protein group all measured iBAQ values are plotted in grey, with the median value in black. **(D)** Averages and variances of peptides per protein group in each experimental group. **(E)** Box plot of LFQ intensity averages across samples within each group. **(F)** Box plot of protein copy numbers per cell (inferred as in [5]) averaged across samples within experimental groups. **(G)** Summed LFQ intensities averaged within experimental groups. **(H)** Total mass of identified proteins per cell, averaged within experimental groups. The inferred protein copy number per cell was divided by Avogrado's number (6.0221409 x 10^{23}) and then multiplied by the protein mass in kilodaltons (kDa), yielding the total mass of identified proteins per cell.

Figure 4. Distributions of **(A)** molecular mass and **(B)** amino acid sequence length; smaller and shorter proteins are the most prevalent. When plotted against protein LFQ intensity **(C, D) or** protein copy number (inferred as in [5]) **(E, F)**, weak (if any, see R^2 values on figurines) correlations are observed, suggesting that protein abundance estimation is not biased by peptide mass or length (also see Figure 2B).

Figure 5. Variance quantification of individual samples. (A, C) Cumulative plots of the coefficient of variance (CV) in the number of identified peptides in each experimental group. Including proteins with at least one identified peptide (A), CVs of 30% or less are found in ~74%. With an increased cut-off (10 peptides) ~70% of proteins show a CV of 15% or less (C). **(B, D)** Principal component analysis (PCA) using numbers of identified peptides per protein. With a cut-off of 1 identified peptide, ~55% of variance is explained by Principal Component (PC) 1 and 2 (B); this is ~76% when a cut-off of 10 identified peptides is used (D). **(E)** Cumulative plot of the CV of inferred [5] protein copy numbers per cell per experimental group.

On average, ~73% of proteins show a CV of 45% or less. **(F)** PCA based on inferred protein copy numbers per cell. PC1 and 2 explain ~73% of variance, and samples cluster mostly based on cortical laminar origin.

Figure 6. Matrix of correlation coefficients of biological and technical replicates. Data from **(A)** all biological samples and their corresponding replicates combined across experimental groups and cortical layers, **(B)** L2/3 **(C)** and L4. **(a, b).** Scatter plots showing peptides per protein group (a) or protein copy numbers (inferred copy numbers per cell [5], (b)) for biological samples (X axis) and their technical replicates (Y axis). **(b, d)** Histograms showing differences in identified peptides per protein group (b) or protein copy numbers (d) between biological and technical replicates. Note that across all samples, the variation between the biological sample and the technical replicas are small, with Pearson R^2 values between 0.89-96.

Supplemental Figure 1. Correlation between LFQ and protein copy numbers. Scatter plots of LFQ values (x axis) and inferred protein copy numbers [5] (y axis), showing a linear correlation between the two quantification methods ($R^2 > 0.75$).

Supplemental Figure 2. Distribution of peptides per protein group in biological and technical replicates. Scatter plots of identified peptides per protein group from biological and technical replicates. Red-bordered graphs indicate pairwise comparisons between biological samples and their direct technical replicate; graphs with black borders contain the remaining comparisons. Overall, a strong linear correlation is observed in pairwise comparisons ($R^2 = 0.95 \pm 0.01$), in particular between biological and technical replicate pairs ($R^2 \geq 0.96 \pm 0.01$). Scale bars correspond to 100 peptides.

Supplemental Figure 3. Copy number distribution of biological and technical replicates. Log-log plots showing protein copy numbers from biological and technical replicates. Pairwise comparisons between biological samples and their direct technical replicate are indicated by red borders; black borders indicate the remaining comparisons. As in **Supplemental Figure 2**, pairwise comparisons show high correlations between individual samples (average $R^2 = 0.90 \pm 0.01$), which is highest for biological and technical replicate pairs ($R^2 = 0.93 \pm 0.03$).

Figure 1 Click here to download Figure Figure1.pdf

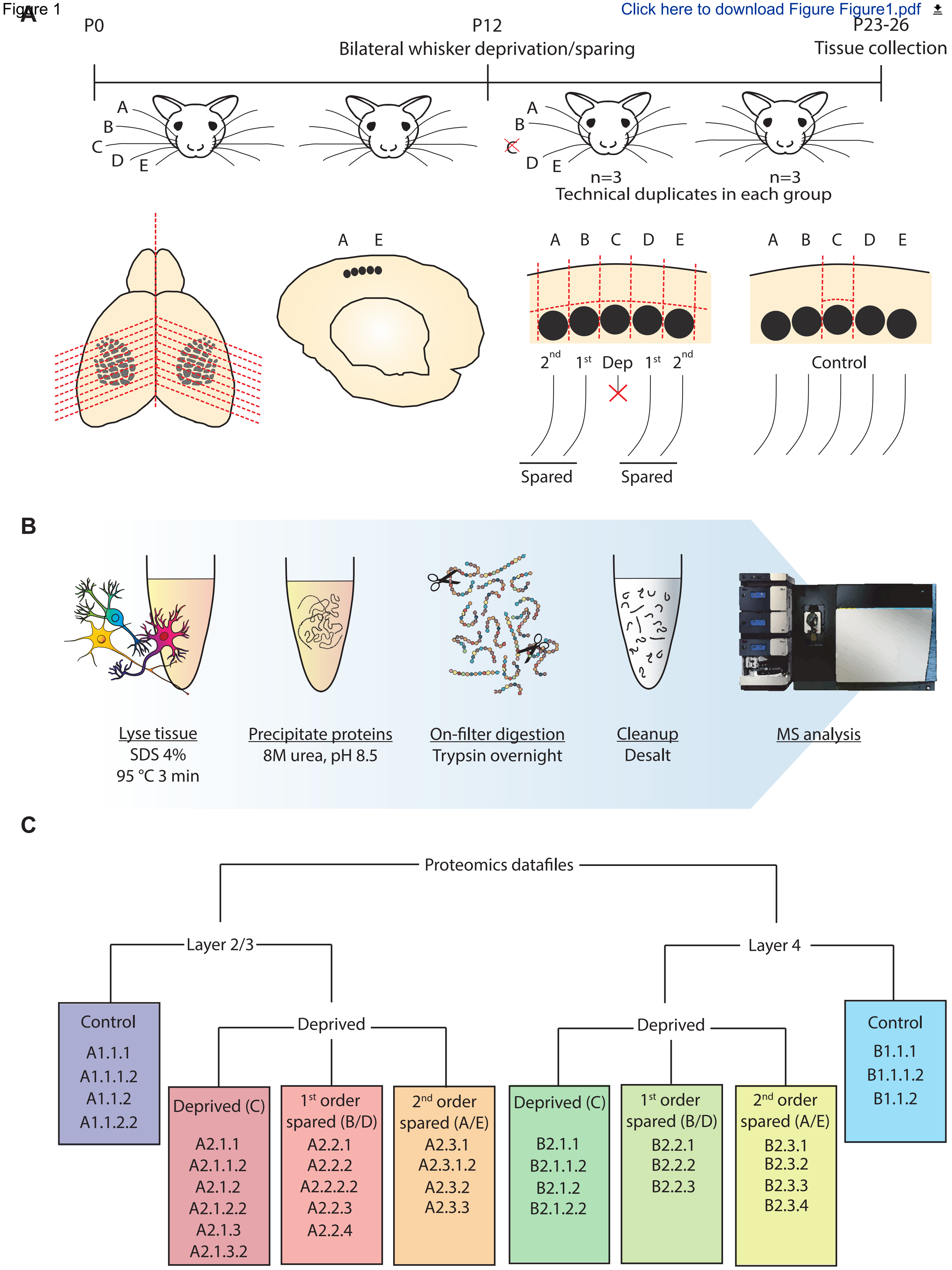

Figure 2

Click here to download Figure Figure2.pdf

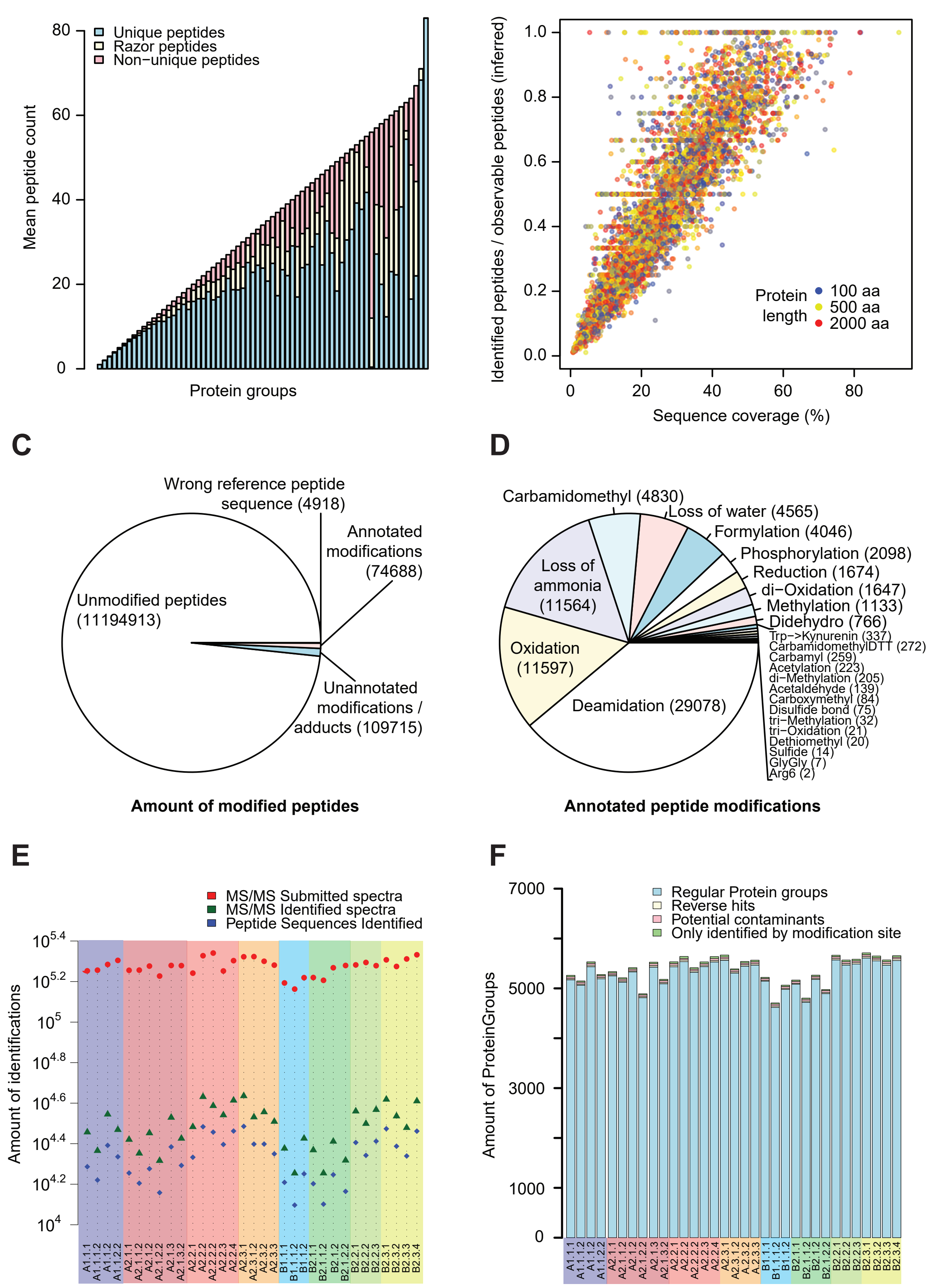

Click here to download Figure Figure3.pdf

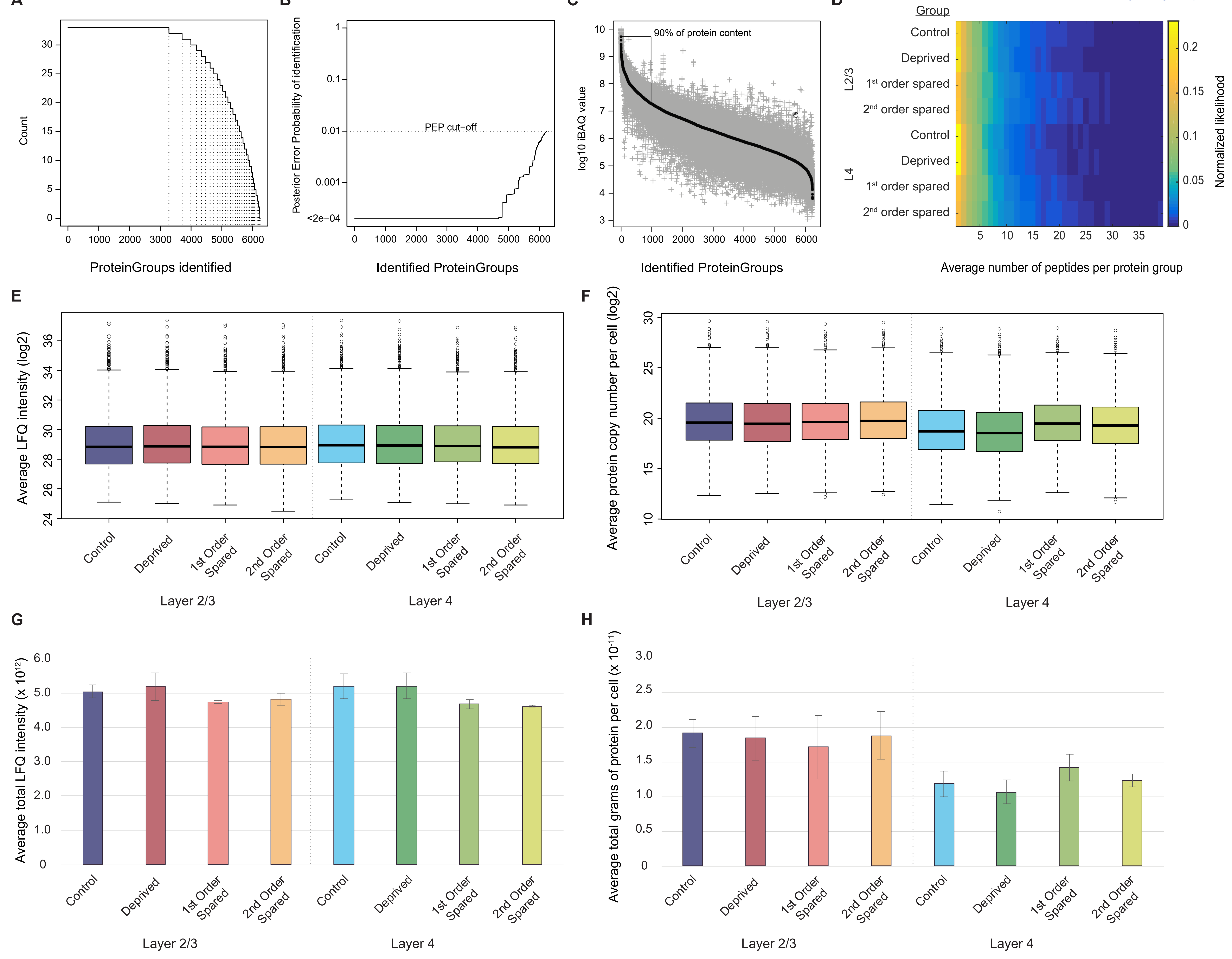

Click here to download Figure Figure4.pdf

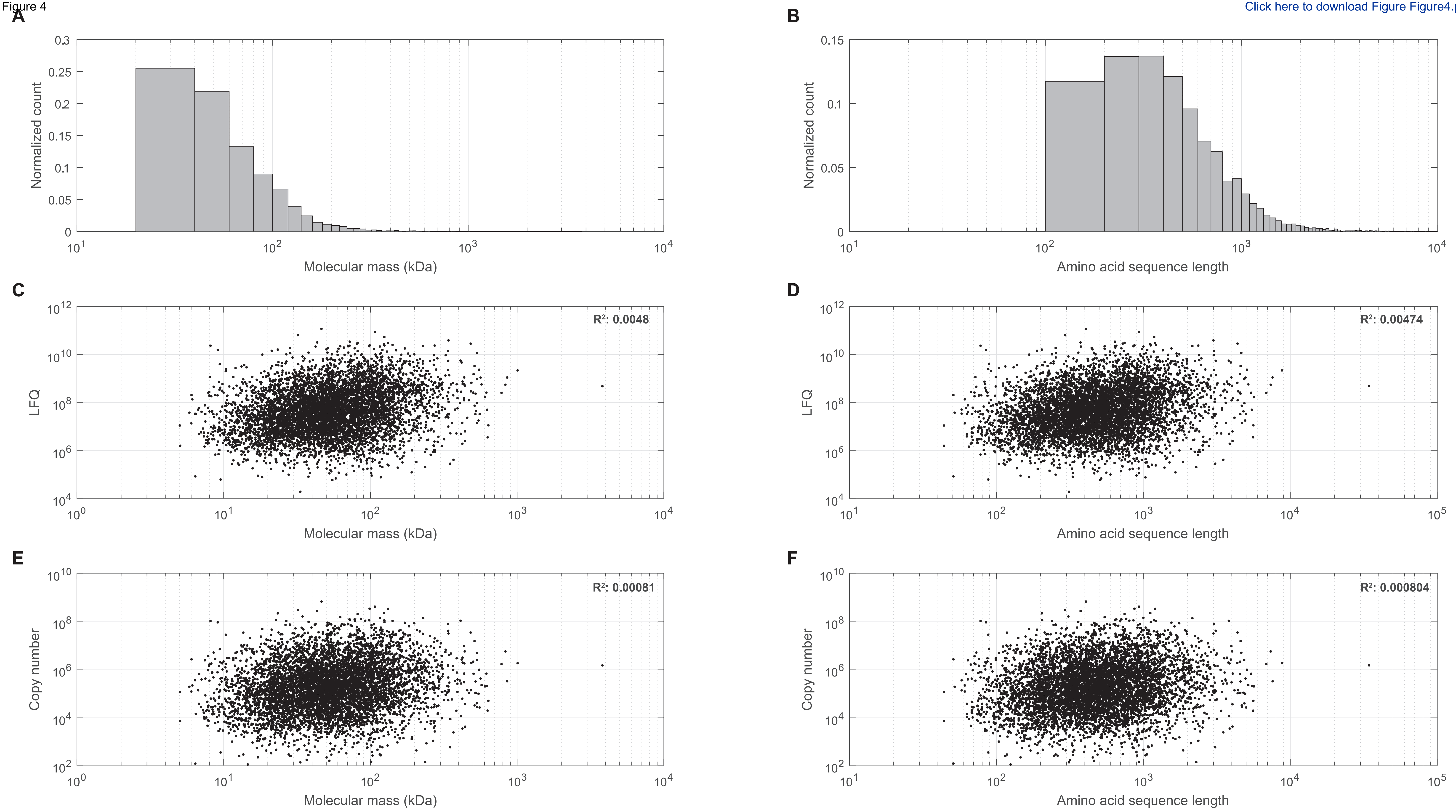

Figure 5

Click here to download Figure Figure5.pdf

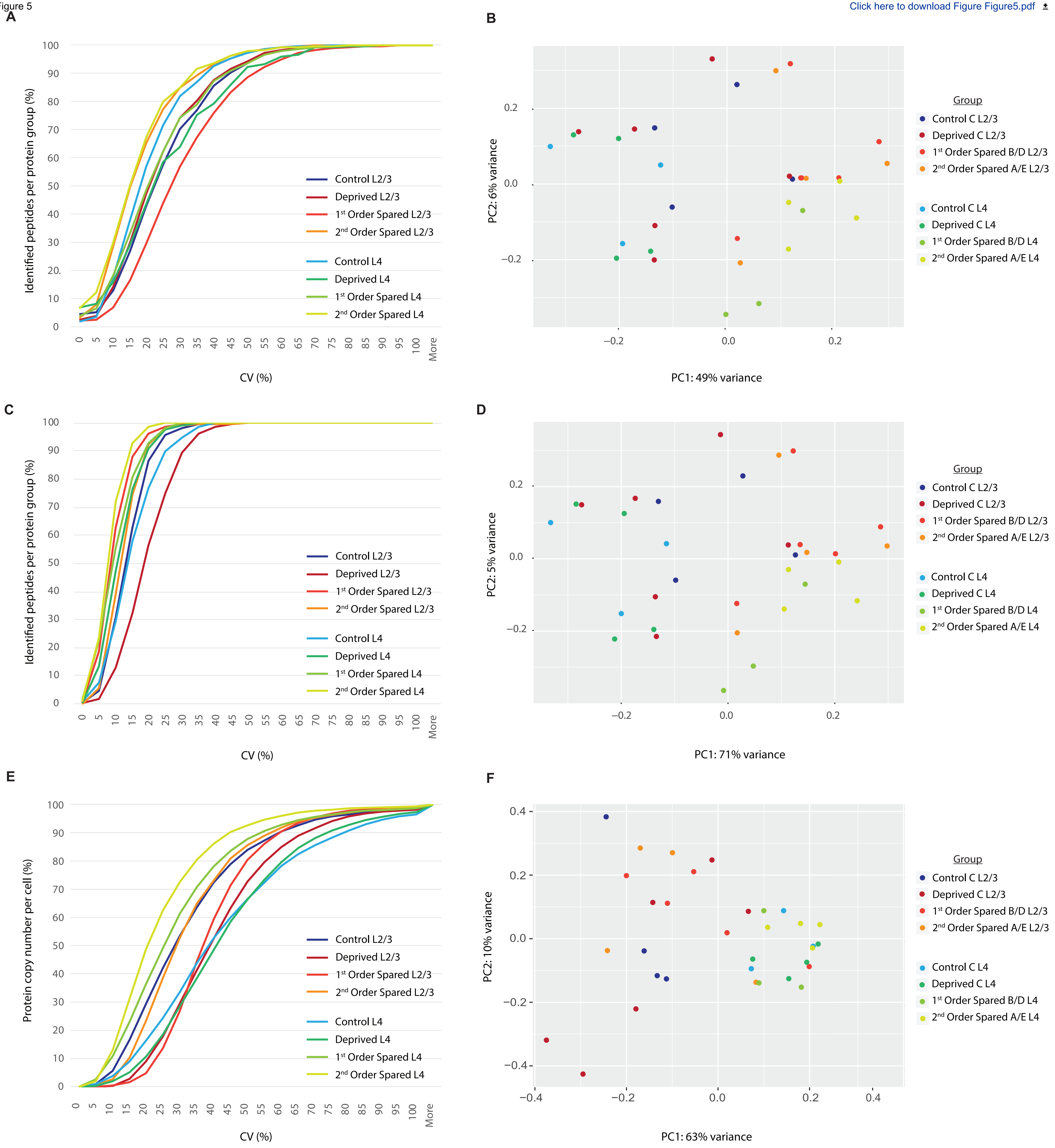

Figure 6

Click here to download Figure Figure6.pdf

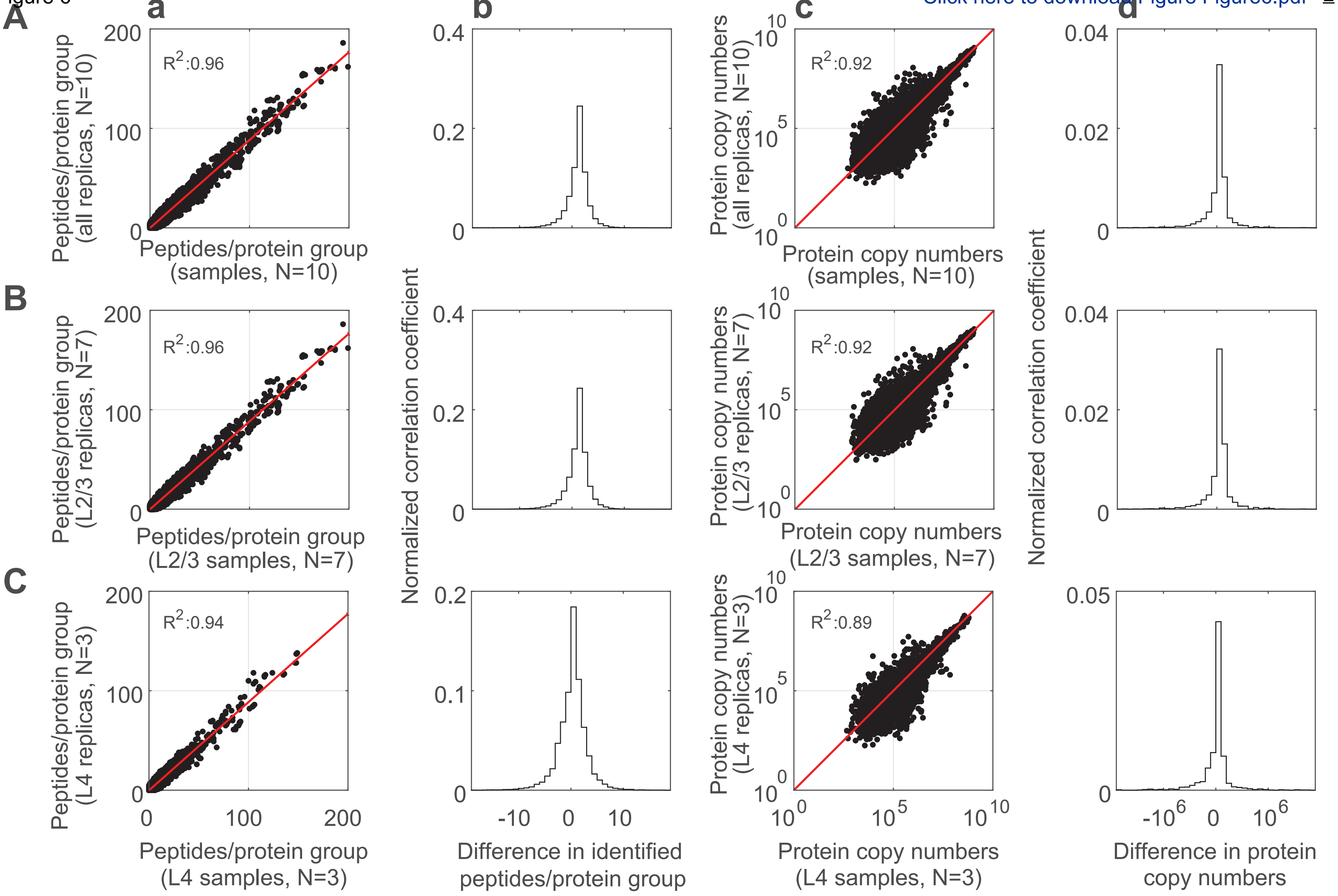

Supplemental Figure 1

Click here to access/download
Supplementary Material
Supplemental_Figure1.pdf

Supplemental Figure 2

Click here to access/download
Supplementary Material
Supplemental_Figure2.pdf

Supplemental Figure 3

Click here to access/download
Supplementary Material
Supplemental_Figure3.pdf
