# Supplementary material for: Proteomic landscape of the primary somatosensory cortex upon sensory deprivation
Source: Gigascience. 2017 Aug 23;6(10):1–10. doi: 10.1093/gigascience/gix082 (PMC5632293; doi:10.1093/gigascience/gix082)
Supplement: GIGA-D-17-00057_Revision-1.pdf [file gix082_GIGA-D-17-00057_Revision-1.pdf]

Manuscript with DOI
Click here to download Manuscript Gigascience_Kole_Lindeboom_R1 NN_KK.docx

Proteomic landscape of the primary somatosensory cortex upon sensory deprivation

Koen Kole[1,2]*, Rik G.H. Lindeboom[3]*, Marijke P.A. Baltissen[3], Pascal W.T.C. Jansen[3], Michiel Vermeulen[3], Paul Tiesinga[2], Tansu Celikel[1]

(1) Department of Neurophysiology, (2) Department of Neuroinformatics, Donders Institute for Brain, Cognition, and Behaviour, Radboud University, Nijmegen - the Netherlands. (3) Department of Molecular Biology, Radboud Institute for Molecular Life Sciences, Radboud University, Nijmegen - the Netherlands

* indicates equal contribution

E-mail addresses (in the order of appearance): k.kole@neurophysiology.nl, r.lindeboom@science.ru.nl, m.baltissen@science.ru.nl, p.jansen@science.ru.nl, michiel.vermeulen@science.ru.nl, p.tiesinga@science.ru.nl, celikel@neurophysiology.nl (corresponding author)

Abstract

Background: Experience-dependent plasticity (EDP) powerfully shapes neural circuits by inducing long-lasting molecular changes in the brain. Molecular mechanisms of EDP have been traditionally studied by identifying single or small subsets of targets along the biochemical pathways that link synaptic receptors to nuclear processes. Recent technological advances in large-scale analysis of gene transcription and translation now allow systematic observation of thousands of molecules simultaneously. Here we employed label-free quantitative mass spectrometry to address experience-dependent changes in the proteome after sensory deprivation of the primary somatosensory cortex.

Findings: Cortical column- and layer-specific tissue samples were collected from control animals, with all whiskers intact, and animals whose C-row whiskers were bilaterally plucked for 11-14 days. 33 samples from cortical layers (L) 2/3 and L4 spanning across control, deprived, 1st and 2nd order spared columns yielded at least 10,000 peptides mapping to ~5000 protein groups. Of these, 4,676 were identified with high confidence and >3000 are found in all samples.

Conclusions: This comprehensive database provides a snapshot of the proteome after whisker deprivation, a protocol that has been widely used to unravel the synaptic, cellular and network mechanisms of EDP. Complementing the recently made available transcriptome for identical experimental conditions (see accompanying article by Kole et al), the database can be used to (1) mine novel targets whose translation is modulated by sensory organ use, (2) cross-validate experimental protocols from the same developmental time point, and (3) statistically map the molecular pathways of cortical plasticity at a columnar and laminar resolution.

Keywords: Barrel cortex, whisker plucking, juvenile mice, mass-spectrometry, label-free quantification, proteomics

Data Description

Context

Sensory experience shapes neural circuits throughout life via experience-dependent plasticity (EDP). Changes in neural circuits, in turn, allow the brain to adapt to recent sensory, motor and perceptual experiences of animals in their ever-changing environments.

The rodent barrel cortex, a subfield of the primary somatosensory cortex, processes sensory information originating from whiskers. Each cortical (barrel) column receives majority of its sensory input from one (so called principal) whisker, anatomically delineating the neural circuits associated with each whisker. Taking advantage of this organizational principle, previous studies have shown that targeted deprivation of select whiskers result in weakening of the sensory evoked responses in synaptic projections originating from barrel cortical layer (L)4 and targeting L2/3 in an experience-dependent manner [1, 2]. In contrast, corresponding projections in the neighbouring sparing whiskers' cortical columns are strengthened [3]. The molecular mechanisms of EDP, however, are still largely unknown. Understanding how sensory experience shapes neuronal circuits will benefit from systematic analysis of the transcriptome and proteome following altered sensory experience. In an accompanying manuscript we have provided a snapshot of the transcriptomic changes after 11-12 days long sensory deprivation resolved across cortical columns and layers (Kole et al, submitted). The database presented herein employs the same sensory deprivation protocol but focuses on the proteomic changes across cortical layers of L4 and L2/3 in columnar resolution.

Methods

Animals: All experiments were performed in accordance with NIH Guidelines for the Care and

Use of Laboratory Animals, and were approved by the Animal Ethics Committee of the Radboud University in Nijmegen, the Netherlands. Pregnant wild type mice (C57Bl6; Charles River, stock number 000664 [RRID:NCBITaxon_10090) were kept at a 12 hour light/dark cycle with access to food *ad libitum*. Cages were checked for birth daily. Experience-dependent plasticity was induced as described previously (Kole et al, submitted). Briefly, at P12, C-row whiskers were plucked under isoflurane anaesthesia while control animals were not plucked but anaesthetized and handled similarly (**Figure 1A**). Animals across groups were housed together with their mothers until tissue collection at P23-P26.

[Figure 1 comes about here]

Slice preparation and sample collection: Tissue samples were collected from acute brain slices as described before [4]. In short, pups were deeply anaesthetized using isoflurane and perfused with ice-cold carbogenated slicing medium before 400 µm thalamocortical slices [1] were prepared. Slices were incubated in carbogenated aCSF at 37 degrees Celcius for 30 min before they were transferred to a holding chamber containing carbogenated aCSF in room temperature. Slices remained in this chamber until cortical layers and columns were isolated within ~5-40 min.

For sample isolation, slices were placed under a microscope equipped with Dodt gradient contrast, used for visualization of the granular segments of the live neocortical tissue, such as the L4 in the barrel cortex. Visualized cortical columns (A-E) were separated from each other using a pulled pipette (Sutter Instruments P-2000), tip size of ~5 micrometers, serving as a microneedle. Layers (L) 2/3 and L4 were isolated based on the established contrast criteria commonly used in electrophysiological analysis of barrel cortical neurons in acute slices [1,2].

In the barrel cortex, cortical columns can be grouped by their relative distance to each other. Cortical columns B and D, for example, are named as the 1st order neighboring cortical columns in respect to the C row column. Similarly A and E row columns constitute the 2nd order neighboring columns. To increase the sample yield and have single animal resolution for the proteomic mapping, we pooled the samples within each layer across B and D, and A and E columns. Immediately after dissection, tissue samples were placed in Eppendorf tubes, snap frozen in liquid nitrogen and stored at -80ºC until further use.

In the control group, tissues were collected from three separate mice (biological replicates) whereas the deprived group consisted of four animals. Only C-row layers were sampled in the control animals, as the comparison across the C-rows between control and deprived animals allow to directly address the molecular changes associated with the whisker deprivation. Due to the small tissue sizes, obtaining successful LC-MS runs was technically challenging. Thus, not all laminar samples from all cortical columns are retained for the full analysis (See **Supplemental Table 1** for the distribution of samples across groups). In addition to these biological replicates, we ran 10 of the samples a second time, providing 10 technical replicates.

Lysate preparation and protein digestion: Samples were prepared for mass spectrometry using the filter-aided sample preparation (FASP) method, as described before [5] (**Figure 1B**). Briefly, mouse brain tissues were homogenized in lysis buffer (4% w/v SDS, 100 mM Tris/HCl and 0.1 M DTT, pH 7.6) and incubated at 95 °C for 3 min. To shear DNA and reduce sample viscosity, samples were ultrasonicated. Samples were then clarified by centrifugation, after which the proteins in the extract were denatured using urea buffer (8M urea, 0.1 M Tris/HCl, pH 8.5) and centrifuge-filtered using 30 kDa filters (Microcon YM-30). After washing with urea buffer (pH 8.0), proteins were alkylated with iodoacetamide, followed by washing with ammonium bicarbonate. Trypsin (Promega Cat#V5280) was applied to digest the extracted proteins. The resulting peptides were then collected by centrifugation and desalted using C18 (Empore)

StageTips. Given the small sample size protein yield was not determined before moving on to mass spectrometry.

Mass spectrometry: Tryptic peptides were separated on an Easy-nLC 1000 (Thermo, [RRID:SCR_014993]) using a 214 minute long gradient of acetonitrile (7% to 30%) followed by washes at 60%, followed by 95% acetonitrile for 240 min of total data collection. Mass spectra were collected on a LTQ-Orbitrap Fusion Tribrid mass spectrometer (Thermo, [RRID: SCR_014992]) in data-dependent top-speed mode with dynamic exclusion set at 60 s. Precursor MS spectra are acquired at an m/z range of 400-1500 at a resolution of 120.000 and a target value of 300000 ions per full scan in the Orbitrap. MS/MS spectra are acquired in HCD mode using 35% collision energy and fragmentation spectra are recorded in the ion trap.

Data processing: Raw data was analysed using MaxQuant ([RRID:SCR_014485]) version 1.5.1.0. with match-between-runs, label-free quantification and intensity based absolute quantification (iBAQ) enabled. Dependent peptides were enabled to perform an unbiased search against modifications on the identified peptides. The RefSeq protein sequence database downloaded on 28-06-2016 was used to identify proteins. Identified proteins were filtered for reverse hits and common contaminants. Contaminant proteins were determined by the MaxQuant software suite and include proteins that are often introduced during a typical mass spectrometry experiment such as keratins and trypsin. All other processing was performed in MATLAB ([RRID:SCR_001622]) or R ([RRID:SCR_001905]) programming languages.

Data validation and quality control

Peptides were assigned to protein groups based on shared peptide sequences, the majority of which consist mainly of unique peptide sequences (71%, **Figure 2A**). Razor peptides (i.e. peptides that can be assigned to more than one protein but are assigned to the protein group with

the most other peptides, i.e. Occam's razor principle) on average made up 13% of the designated protein groups; non-unique peptides on average constituted 16%. When testing how much of the total and theoretically observable protein sequence length was identified by the analyses, we observe for most proteins a good coverage of the theoretically observable peptides (44% on average, **Figure 2B**). Complete sequence coverage is never achieved, likely because of the remaining tryptic peptides being too long or too short to be measured by mass spectrometry. Since high numbers of peptide modifications and adducts can interfere with accurate protein quantification, we assessed the types of peptide modifications that we could observe on the identified peptides (**Figure 2C** and **2D**). Peptide modifications may occur in vivo but more likely arise during the sample preparation steps. Reassuringly, the majority of peptides (98.33%) were found to be unmodified. For 0.96% of the peptides we found a modified form with an unannotated mass shift, while 0.65% of peptides was modified and had a mass shift that could be annotated to a known peptide modification (**Figure 2C**). In total we could identify 25 different types of peptide modifications (**Figure 2D**). Of these, the top three modifications were deamidation (38.94%), oxidation (15.53%) and loss of ammonia (15.48%), which are all common peptide modifications. Next, we addressed the data quality for individual samples, which showed that on average 23,489 unique amino acid sequences (ranging from 13,095 to 72,418) could be identified per sample (**Figure 2E**); the majority of these (>98%) could be assigned to regular protein groups, excluding reverse hits, contaminants or peptides identified only by modification. Reverse hit rate (i.e. false discovery rate) or the number of proteins that could only be identified based on a modified peptide was never higher than 0.7%, suggesting high confidence of protein identification. Additionally, the number of potential contaminants was low for all samples (minimum, 29; first quartile, 33; median, 35; mean, 34.52; third quartile, 36; maximum, 38), suggesting high sample purity (**Figure 2F**).

[Figure 2 is about here]

Of the designated protein groups (i.e. protein groups with a Posterior Error Probability (PEP, confidence of peptide identification) of <0.01, n = 6,245), over 3,000 could be reliably identified in all of our samples (**Figure 3A** and **3B**); peptides in 4,676 protein groups could be identified with high confidence (PEP <0.0002). Of all identified proteins, 90% of the total protein content (as determined by intensity based absolute quantification [7]) was contained in the 979 most abundant proteins (**Figure 3C**). In this dataset we identified and quantified proteins over five orders of magnitude, suggesting high sensitivity even at low protein concentrations.

[Figure 3 is about here]

To estimate the variance in protein quantification across samples, we averaged the number of identified peptides per protein group, which showed similar distributions across experimental groups (**Figure 3D**). Additionally we have performed two different normalizations: 1) Averaging the LFQ intensity and copy number of each protein (as quantified according to the "proteomic ruler" approach [6], which uses the signal intensities of measured histones as an internal normalization) of each protein across samples within groups (**Figure 3E,F**, respectively), and 2) calculating the total LFQ intensity or protein mass across proteins within each sample and averaging across independent samples within a group (**Figure 3G,H**, respectively). In the former, we included only those proteins that had a protein copy number of non-zero. The results showed that independent of the method of quantification the experimental groups were similar to each other, suggesting that comparisons within protein groups between experimental groups should not be hampered by systematic differences in (inferred) protein abundances. Calculating the total mass of identified proteins per cell (by dividing inferred protein copy numbers per cell by

Avogrado's number and multiplying by protein mass in kDa) showed that L2/3 cells on average contain 18.42 ±0.78 picograms of identified protein; this was 12.29 ±1.28 picograms in L4 cells (p = 0.0004, Student's t-test) (**Figure 3H**). The number of identified proteins averaged per group across layers did not differ (p=0.6964, unpaired Student's t-test). Since protein identification rates are likely to be independent from cortical layer identity, these results suggests that total protein levels per cell are lower in L4. To investigate how the two quantification methods (i.e. LFQ and proteomic ruler approach) correspond, we examined the correlation between LFQ intensities and protein copy numbers (**Supplemental Figure 1**). The correlation (R^2) between the two quantification methods ranged from 0.76 to 0.80, suggesting good consensus of protein abundance estimation.

We then assessed the distributions of molecular mass (kDa) and amino acid sequence length of the proteins identified in our samples. On average, proteins were 71.65 ±82.77 kDa in mass (**Figure 4A**) and had a mean length of 643.63 ±745.27 amino acids (**Figure 4B**). To exclude any bias in protein abundance estimation based on protein length, we plotted mass or sequence length against LFQ intensities or estimated protein copy number [5]. This showed only weak, if any, correlations (R^2 values <~0.005) between LFQ intensity or copy number and peptide mass or length, suggesting proteins of all sizes are equally well identified (**Figure 4C, D, E, F**).

[Figure 4 is about here]

Next, we examined the variance between samples by calculating the coefficient of variation (CV) of inferred protein copy numbers [6] (**Figure 5A**). About 73% of proteins showed a CV of 45% or less, on average. We then employed principal component analysis (PCA), which showed

that 72.5% of variance was explained by PC1 and 2, and that samples were clustered mostly by cortical layer (**Figure 5B,C**). These analyses were repeated for identified peptides for each protein group in individual samples, using different cut-offs of identified peptides (**Figure 3D**). When no cut-off was used (i.e. including proteins identified by at least one peptide, see Figure 3D for the distribution across all groups), on average 73.88% of proteins showed a CV of 30% or less (**Supplemental Figure 2A**); With a cut-off of 10 identified peptides, a CV of 15% or less was found for 70.74% of proteins (**Supplemental Figure 2B**). PCA using both of these cutoffs showed that samples cluster mostly around C column-derived samples. Principal components (PC) 1 and 2 explained 77.6% and 86.5% of variance, depending on the cut-off value used (**Supplemental Figure 2C-F**).

[Figure 5 is about here]

Since our dataset contains several technical duplicates, we asked how well they correlate with the biological replicates and compared identified peptides per protein group and protein copy numbers of biological and technical replicates (**Figure 6**). Biological samples and their direct technical replicates were highly correlated ($R^2 \geq 0.89$, **Figure 6A-Ca,c**), which was also found for the remaining pairwise comparisons ($R^2 \geq 0.90$) (**Supplemental Figure 3, 4**). These results suggest that samples are highly comparable in terms of peptide and protein counts, and that sequential nature of the mass spectroscopy does not systematically, or in statistically appreciably fashion, bias protein quantifications, at least in our samples.

[Figure 6 is about here]

Re-use potential

The current dataset provides a proteomics view of the experience-dependent plasticity in the mouse barrel cortex. Since barrel cortex is a popular model system where sensory processing and experience-dependent plasticity are studied from molecules to behavior [e.g. 1-3, 8, 10,12,13], this resource should help to identify some of the molecular underpinnings of cortical plasticity. Given the relatively high anatomical resolution at which samples were collected, the current dataset would also be beneficial in the understanding of molecular constituents of cortical laminar identity and function. It should be noted however that the collected samples contain the entirety of the cellular population, *i.e.* are not cell type-specific. Signals originating from all cell types are thus averaged, which should be considered by researchers reusing this dataset. The cellular complexity of the samples studied herein will be particularly useful for those efforts aiming to identify the neuronal as well as the non-neuronal basis of experience dependent plasticity.

A combinatorial approach between proteomics and transcriptomics (e.g. RNA sequencing; Kole et al., submitted) is a promising outlook that could help identifying those molecular targets that are essential for reorganization of neural networks following sensory deprivation. Proteomics data can aid to broaden the scope of findings from transcriptomics studies as it can provide novel insights into post-transcriptional regulation of protein expression, the time course of protein expression (since proteins typically have a longer half-life than RNAs) and posttranslational modifications that could orchestrate specific protein functions.

Only a few studies are available that focus on large-scale molecular changes in neural circuits following sensory deprivation [9–10]. As large-scale molecular techniques are becoming more accessible, studies employing them to investigate the molecular bases of plasticity are likely to

follow suit. The phenotype of EDP in barrel cortex depends heavily on the experimental approach used (e.g. enrichment vs. deprivation, single whisker experience vs. whole row deprivation, developmental time points [12, 13]). The current dataset should prove useful to validate, expand and compare the findings of molecular studies employing different protocols. Moreover, comparing our dataset with those obtained from other brain regions (e.g. visual cortex, auditory cortex), would help to determine where previously observed differences in plasticity across different brain [13] regions might arise.

Availability of the supporting data

Data supporting this work are available in the GigaScience respository, GigaDB [14]. The raw mass spectrometry proteomics data have been deposited in the ProteomeXchange Consortium via the PRIDE partner repository [15] with the dataset identifier PXD005971.

List of abbreviations

EDP Experience dependent plasticity

L2/3 Cortical Layer 2/3, also known as supragranular layers

L4 Cortical Layer 4, i.e. granular layer

Ethics approval and consent to participate

Not applicable.

Consent for publication

Not applicable

Competing interests

The authors declare no competing interests.

Funding

The current work was funded by the Faculty of Science of the Radboud University, Nijmegen, the Netherlands (grant number 626830 – 6200821) as well as the ALW Open Programme of the Netherlands Organization for Scientific Research (NWO; grant number 824.14.022).

Author contributions

KK performed all experimental manipulations, sample acquisition and prepared the tables and figures, performed bioinformatic analysis. RL performed bioinformatic analysis and prepared the tables and figures. PJ and MB performed sample preparation and mass spectrometry. MV supervised the proteomics pipeline. PT co-supervised the project. TC designed and supervised the project, performed bioinformatic analysis. KK and TC wrote the manuscript. All authors edited otherwise approved the final version of the manuscript.

References

1. Allen CB, Celikel T, Feldman DE (2003) Long-term depression induced by sensory deprivation during cortical map plasticity in vivo. Nat Neurosci 6:291–9. doi: 10.1038/nn1012

2. Celikel T, Szostak VA, Feldman DE (2004) Modulation of spike timing by sensory deprivation during induction of cortical map plasticity. Nat Neurosci 7:534–41. doi: 10.1038/nn1222

3. Clem RL, Celikel T, Barth AL (2008) Ongoing in vivo experience triggers synaptic metaplasticity in the neocortex. Science 319:101–4. doi: 10.1126/science.1143808

4. Kole K, Komuro Y, Provaznik J, Pistolic J, Benes V, Tiesinga P, Celikel T. Transcriptional mapping of the primary somatosensory cortex upon sensory deprivation . GigaScience. 2017 (In Press)

5. Wiśniewski JR, Zougman A, Nagaraj N, Mann M (2009) Universal sample preparation method for proteome analysis. Nat Methods 6:359–362. doi: 10.1038/nmeth.1322

6. Wiśniewski JR, Hein MY, Cox J, Mann M (2014) A “Proteomic Ruler” for Protein Copy Number and Concentration Estimation without Spike-in Standards. Mol Cell Proteomics 13:3497–3506. doi: 10.1074/mcp.M113.037309

7. Schwanhäusser B, Busse D, Li N, Dittmar G, Schuchhardt J, Wolf J, Chen W, Selbach M (2011) Global quantification of mammalian gene expression control. Nature 473:337–342. doi: 10.1038/nature10098

8. Zeisel A, Munoz-Manchado AB, Codeluppi S, Lonnerberg P, La Manno G, Jureus A, Marques S, Munguba H, He L, Betsholtz C, Rolny C, Castelo-Branco G, Hjerling-Leffler J, Linnarsson S (2015) Cell types in the mouse cortex and hippocampus revealed by single-cell RNA-seq. Science (80-) 347:1138–1142. doi: 10.1126/science.aaa1934

9. Butko MT, Savas JN, Friedman B, Delahunty C, Ebner F, Yates JR, Tsien RY (2013) In vivo quantitative proteomics of somatosensory cortical synapses shows which protein levels are modulated by sensory deprivation. Proc Natl Acad Sci U S A 110:E726-35. doi:10.1073/pnas.1300424110/DCSupplemental.www.pnas.org/cgi/doi/10.1073/pnas.1300424110

10. Vallès A, Boender AJ, Gijsbers S, Haast R a M, Martens GJM, de Weerd P (2011) Genomewide analysis of rat barrel cortex reveals time- and layer-specific mRNA expression changes related to experience-dependent plasticity. J Neurosci 31:6140–58. doi: 10.1523/JNEUROSCI.6514-10.2011

11. Fernández-Montoya J, Buendia I, Martin YB, Egea J, Negredo P, Avendaño C (2016) Sensory Input-Dependent Changes in Glutamatergic Neurotransmission- Related

Genes and Proteins in the Adult Rat Trigeminal Ganglion. Front Mol Neurosci 9:132. doi: 10.3389/fnmol.2016.00132

12. Feldman DE, Brecht M (2005) Map plasticity in somatosensory cortex. Science 310:810–5. doi: 10.1126/science.1115807

13. Fox K, Wong ROL (2005) A comparison of experience-dependent plasticity in the visual and somatosensory systems. Neuron 48:465–77. doi: 10.1016/j.neuron.2005.10.013

14. Kole K, Baltissen M, Lindeboom R, Jansen P, Vermeulen M, Tiesinga P, Celikel T: Supporting data for "Proteomic landscape of the primary somatosensory cortex upon sensory deprivation" GigaScience Database. 2017. http://dx.doi.org/10.5524/100336

15. Vizcaíno JA, Csordas A, del-Toro N, Dianes JA, Griss J, Lavidas I, Mayer G, PerezRiverol Y, Reisinger F, Ternent T, Xu QW, Wang R, Hermjakob H. (2016) 2016 update of the PRIDE database and related tools. Nucleic Acids Res. 1;44(D1): D447-D456. doi: 10.1093/nar/gkv1145

Figure legends

Figure 1. Overview of the experimental setup, sample collection and data organization.

(A) Pups were bilaterally spared or deprived of their C-row whiskers between P12 and P23-P26. Whisker deprivation, i.e. plucking, was repeated every third day to ensure that there was no regrowth of the whiskers. **(B)** Proteins were denatured and purified, followed by on-filter digestion into tryptic peptides, which were subsequently desalted on C18 StageTips and sequenced on a mass spectrometer. **(C)** Organization of data files in the database. Colours correspond to the colour code codes in Figures 2,3, and 5, as well as the MS output file among the Supplemental Data. Sample codes of 5 digits (e.g. A2.1.1.2) indicate a technical replicate of the sample listed above it (e.g. A2.1.1). See Supplemental Table 1 for mapping of samples to mouse IDs.

Figure 2. Overview of protein groups, sequence coverage and peptide modifications. (A) Stack representation of designated protein groups with the mean contents of unique, razor and non-unique peptides represented in blue, yellow and red, respectively. **(B)** Sequence coverage of identified proteins was plotted as total protein sequence coverage against coverage of theoretically observable peptides (as determined by MaxQuant). **(C)** All identified peptides. **(D)** Identified peptide modifications with an annotated mass shift. **(E)** Submitted and identified MS spectra and uniquely identified amino acid sequences per sample. **(F)** Peptide and protein group identification confidence per sample. Colour coding corresponds to the experimental groups' in Figure 1C.

Figure 3. Quantification of protein groups across all samples. Control/Deprived, C column; 1st order spared, B/D columns; 2nd order spared, A/E columns (see Figure 1). **(A)** Number of observations per protein group in the entire dataset. **(B)** Confidence of protein group identification across samples. **(C)** Protein content versus identified protein groups. For every protein group all measured iBAQ values are plotted in grey, with the median value in black. **(D)** Averages and variances of peptides per protein group in each experimental group. **(E)** Box plot of LFQ intensity averages across samples within each group. **(F)** Box plot of protein copy numbers per cell (inferred as in [6]) averaged across samples within experimental groups. **(G)** Summed LFQ intensities averaged within experimental groups. **(H)** Total mass of identified proteins per cell, averaged within experimental groups. The inferred protein copy number per cell was divided by Avogrado's number (6.0221409 x 10^{23}) and then multiplied by the protein mass in kilodaltons (kDa), yielding the total mass of identified proteins per cell.

Figure 4. Protein quantification is independent from peptide mass or length. Distributions of **(A)** molecular mass and **(B)** amino acid sequence length; smaller and shorter proteins are the most prevalent. When plotting peptide mass or length against protein LFQ intensity **(C, D)** or protein copy number (inferred as in [6]) **(E, F)**, weak (if any, see R^2 values on figurines) correlations are observed, suggesting that protein abundance estimation is not biased by peptide mass or length (also see Figure 2B).

Figure 5. Variance quantification of individual samples. **(A)** Cumulative plot of the coefficient of variation (CV) of the inferred protein copy numbers [6] per cell and per experimental group. On average, ~73% of proteins show a CV of 45% or less. **(B)** Principal Component Analysis (PCA) based on inferred protein copy numbers per cell. Principal Component (PC) 1 and 2 explain ~73% of variance, and samples cluster mostly based on cortical laminar origin. **(C)** Cumulative plot of percent variance explained by each PC. The first 5 PCs explain 85% of the variance.

Figure 6. Matrix of correlation coefficients of biological and technical replicates. Data from **(A)** all biological samples and their corresponding replicates combined across experimental groups and cortical layers, **(B)** L2/3 **(C)** and L4. **(a, b).** Scatter plots showing peptides per protein group (a) or protein copy numbers (inferred copy numbers per cell [6], (b)) for biological samples (X axis) and their technical replicates (Y axis). **(b, d)** Histograms showing differences in identified peptides per protein group (b) or protein copy numbers (d) between biological and technical

replicates. Note that across all samples, the variation between the biological sample and the technical replicas are small, with Pearson R^2 values between 0.89-96.

Supplemental Figure 1. Correlation between LFQ and protein copy numbers. Scatter plots of LFQ values (x axis) and inferred protein copy numbers [6] (y axis), showing a linear correlation between the two quantification methods ($R^2 > 0.75$).

Supplemental Figure 2. Variance quantification of individual samples. (A, B) Cumulative plots of the coefficient of variance (CV) in the number of identified peptides in each experimental group. Including proteins with at least one identified peptide (A), CVs of 30% or less are found in ~74%. With an increased cut-off (10 peptides) ~70% of proteins show a CV of 15% or less (C). **(C, D)** Principal component analysis (PCA) using numbers of identified peptides per protein. With a cut-off of 1 identified peptide, ~78% of variance is explained by Principal Component (PC) 1 and 2 (B); this is ~87% when a cut-off of 10 identified peptides is used (D). **(E, F)** Cumulative plots of showing the percent variance explained by each PC. With a cutoff of 1 identified peptide (C) the first 5 PCs explain ~83% of the variance; using a cutoff of 10 peptides (F) this is ~91%.

Supplemental Figure 3. Distribution of peptides per protein group in biological and technical replicates. Scatter plots of identified peptides per protein group from biological and technical replicates (See Figure 1C for coding). Red-bordered graphs indicate pairwise comparisons between biological samples and their direct technical replicate; graphs with black

borders contain the remaining comparisons. Overall, a strong linear correlation is observed in pairwise comparisons (R^2 = 0.95 ±0.01), in particular between biological and technical replicate pairs ($R^2 \geq$ 0.96 ±0.01). Scale bars correspond to 100 peptides per protein group.

Supplemental Figure 4. Copy number distribution of biological and technical replicates. Log-log plots showing protein copy numbers from biological and technical replicates. Pairwise comparisons between biological samples and their direct technical replicate are indicated by red borders; black borders indicate the remaining comparisons. As in **Supplemental Figure 2**, pairwise comparisons show high correlations between individual samples (average R^2 = 0.90 ±0.01), which is highest for biological and technical replicate pairs (R^2 = 0.93 ±0.03).

Supplemental Table 1. Origin and distribution of samples. Colours correspond to those in Figure 1C. Samples that were run once are marked X, technically duplicated samples are marked XX.

Supplemental Table 2. R commands for PCA analysis and plots.

Figure 1
Click here to download Figure Figure1_Revised.pdf

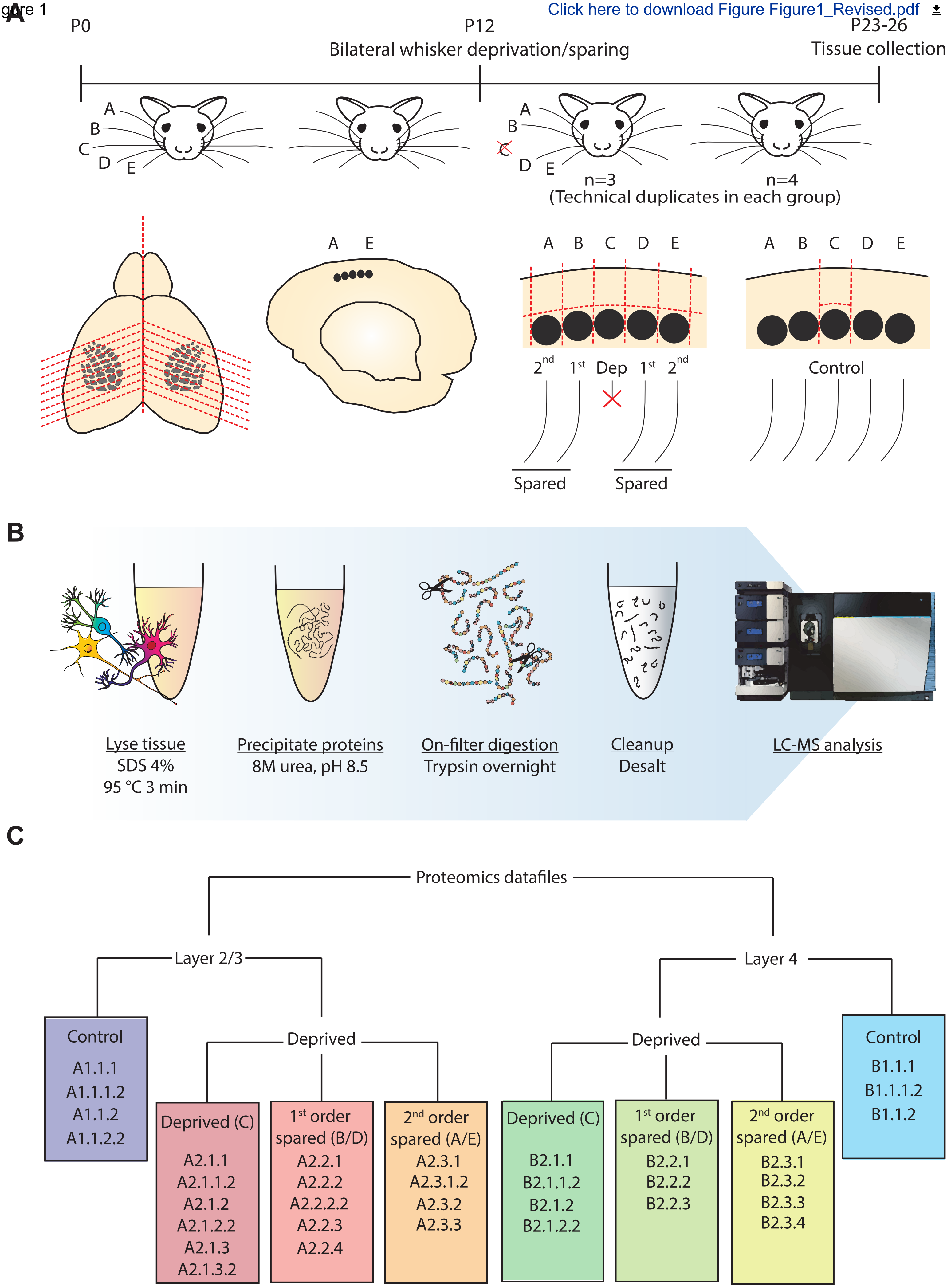

Click here to download Figure Figure2_Revised.pdf

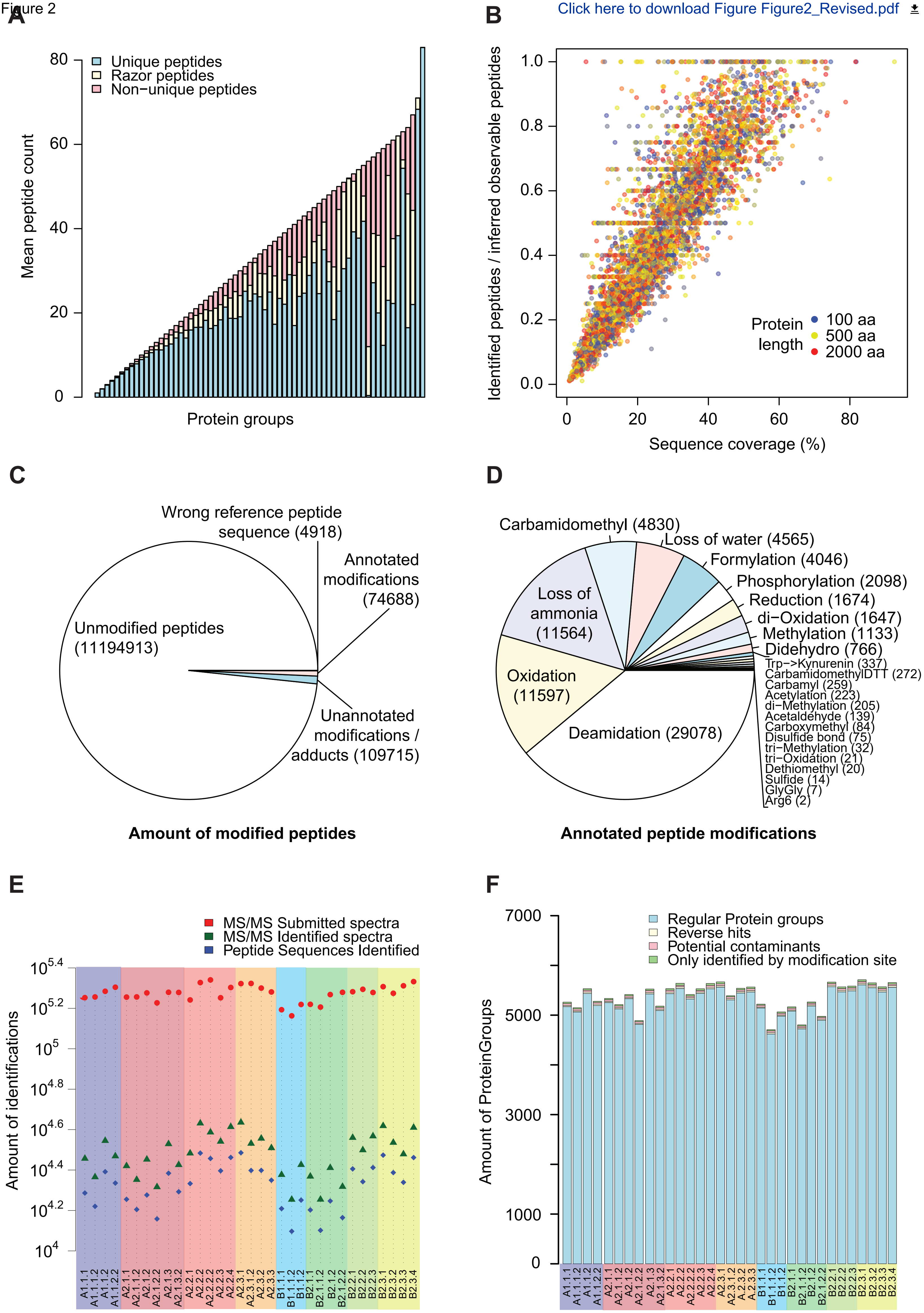

Figure 3

Click here to download Figure Figure3_Revised.pdf

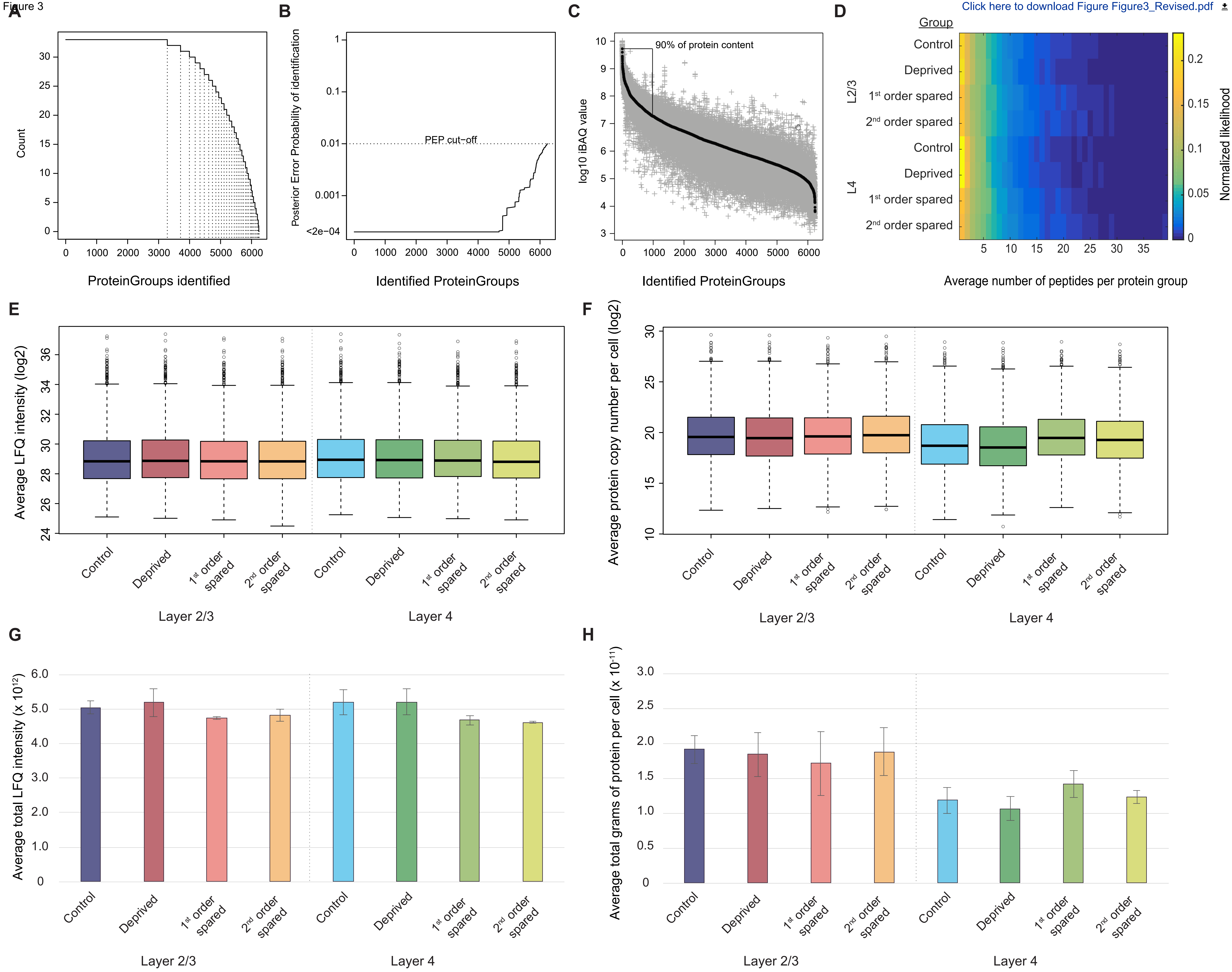

Figure 4

Click here to download Figure Figure4_Revised.pdf

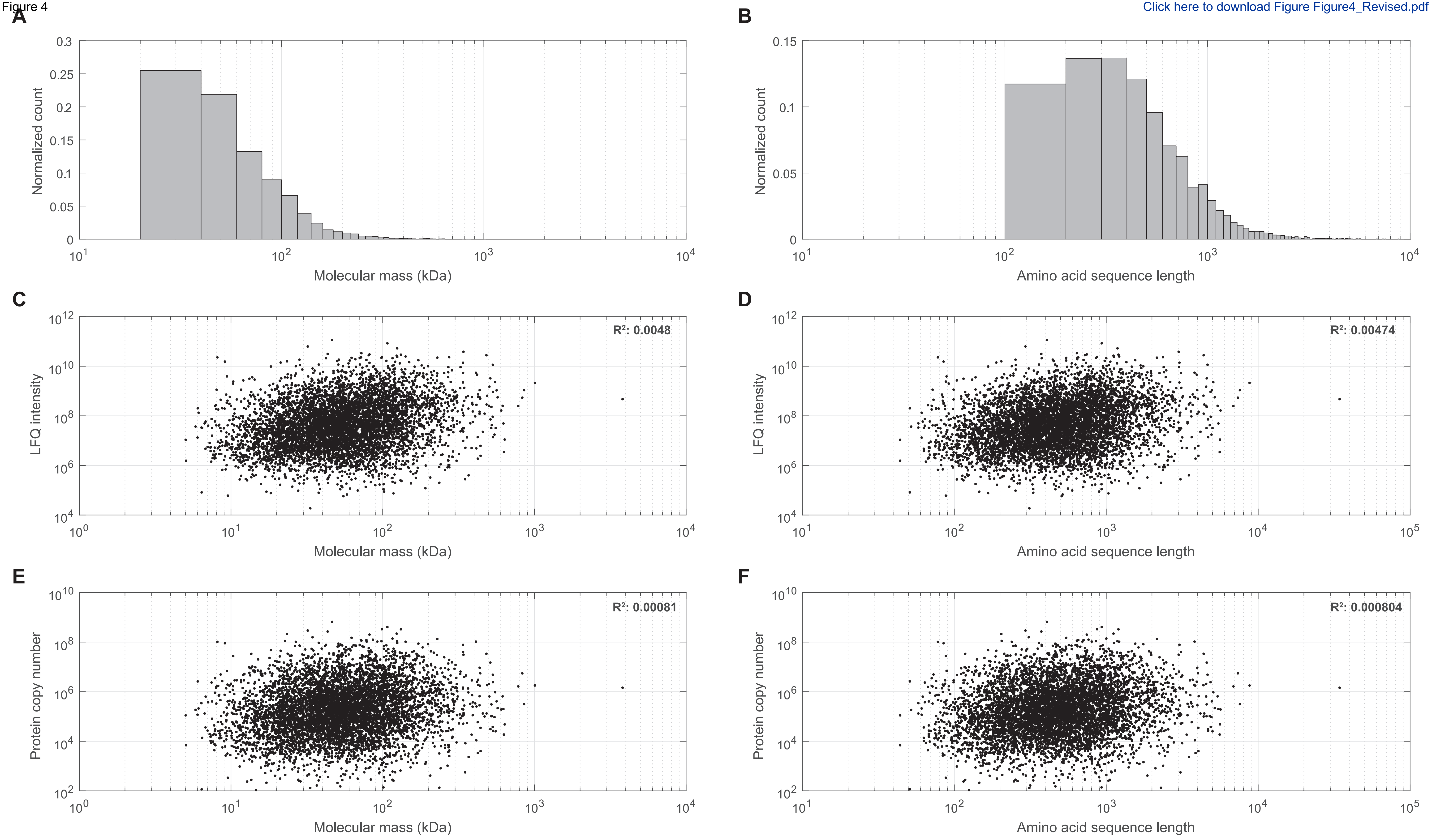

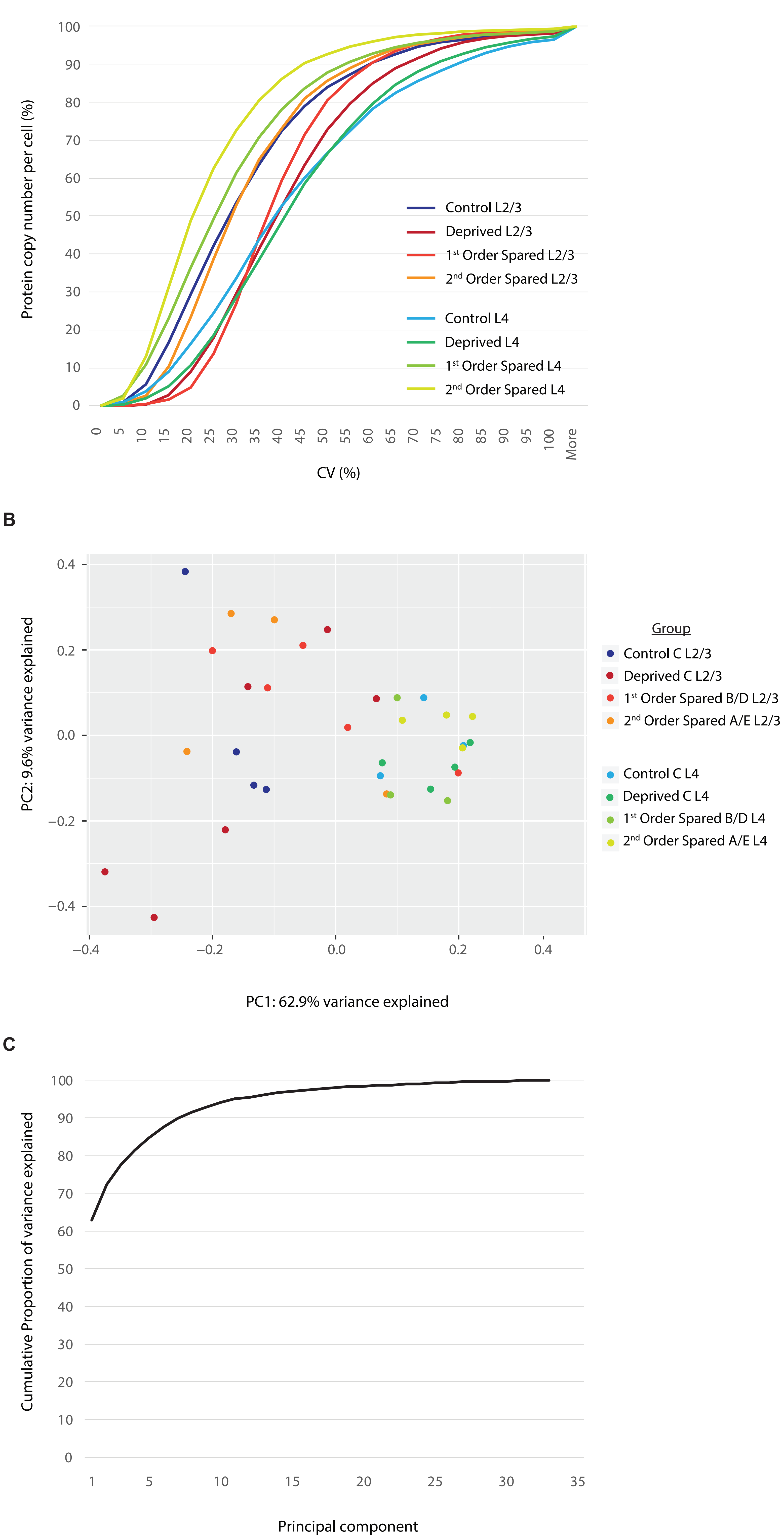
A
Protein copy number per cell (%)
CV (%)
Control L2/3
Deprived L2/3
1st Order Spared L2/3
2nd Order Spared L2/3
Control L4
Deprived L4
1st Order Spared L4
2nd Order Spared L4
More
B
PC2: 9.6% variance explained
PC1: 62.9% variance explained
Group
Control C L2/3
Deprived C L2/3
1st Order Spared B/D L2/3
2nd Order Spared A/E L2/3
Control C L4
Deprived C L4
1st Order Spared B/D L4
2nd Order Spared A/E L4
C
Cumulative Proportion of variance explained
Principal component

Figure 6

Click here to download Figure Figure6_Revised.pdf

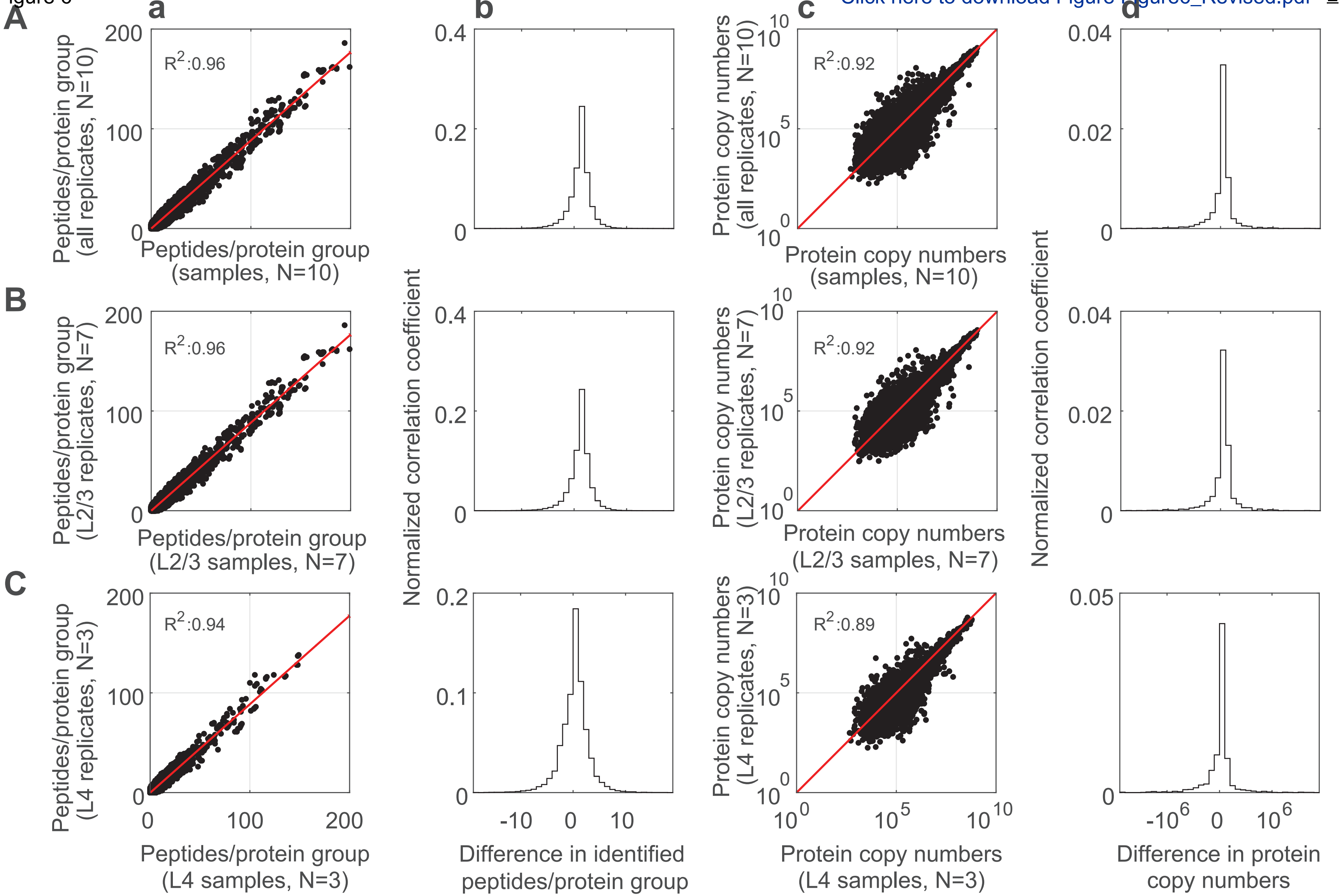

Supplemental Figure 1

Click here to access/download
Supplementary Material
Supplemental_Figure1.pdf

Supplemental Figure 2

Click here to access/download
Supplementary Material
Supplemental_Figure2_Vertical.pdf

Supplemental Figure 3

Click here to access/download
Supplementary Material
Supplemental_Figure3_Revised.pdf

Supplemental Figure 4

Click here to access/download
Supplementary Material
Supplemental_Figure4_Revised.pdf

Supplementary Table 1

Click here to access/download
Supplementary Material
Supplemental_Table1_Revised.pdf

Supplemental Table 2

Click here to access/download
Supplementary Material
Supplemental_Table2_RCommandline_Fig5.txt
