# Supplementary material for: Proteomic landscape of the primary somatosensory cortex upon sensory deprivation
Source: Gigascience. 2017 Aug 23;6(10):1–10. doi: 10.1093/gigascience/gix082 (PMC5632293; doi:10.1093/gigascience/gix082)
Supplement: Supplement materials [file gix082_Supp.zip › Supplemental_Table1_Revised.pdf]

	L2/3				L4			
**Mouse number**	**Con-trol**	**De-prived**	**First order spared**	**Second order spared**	**Con-trol**	**De-prived**	**First order spared**	**Second order spared**
21					XX			
37	XX				X			
38	XX							
20			X	XX			X	X
24		XX	XX				X	X
36		XX	X	X		XX	X	X
39		XX	X	X		XX		X
