# Supplementary figures and images for: Proteomic landscape of the primary somatosensory cortex upon sensory deprivation
Source: Gigascience. 2017 Aug 23;6(10):1–10. doi: 10.1093/gigascience/gix082 (PMC5632293; doi:10.1093/gigascience/gix082)

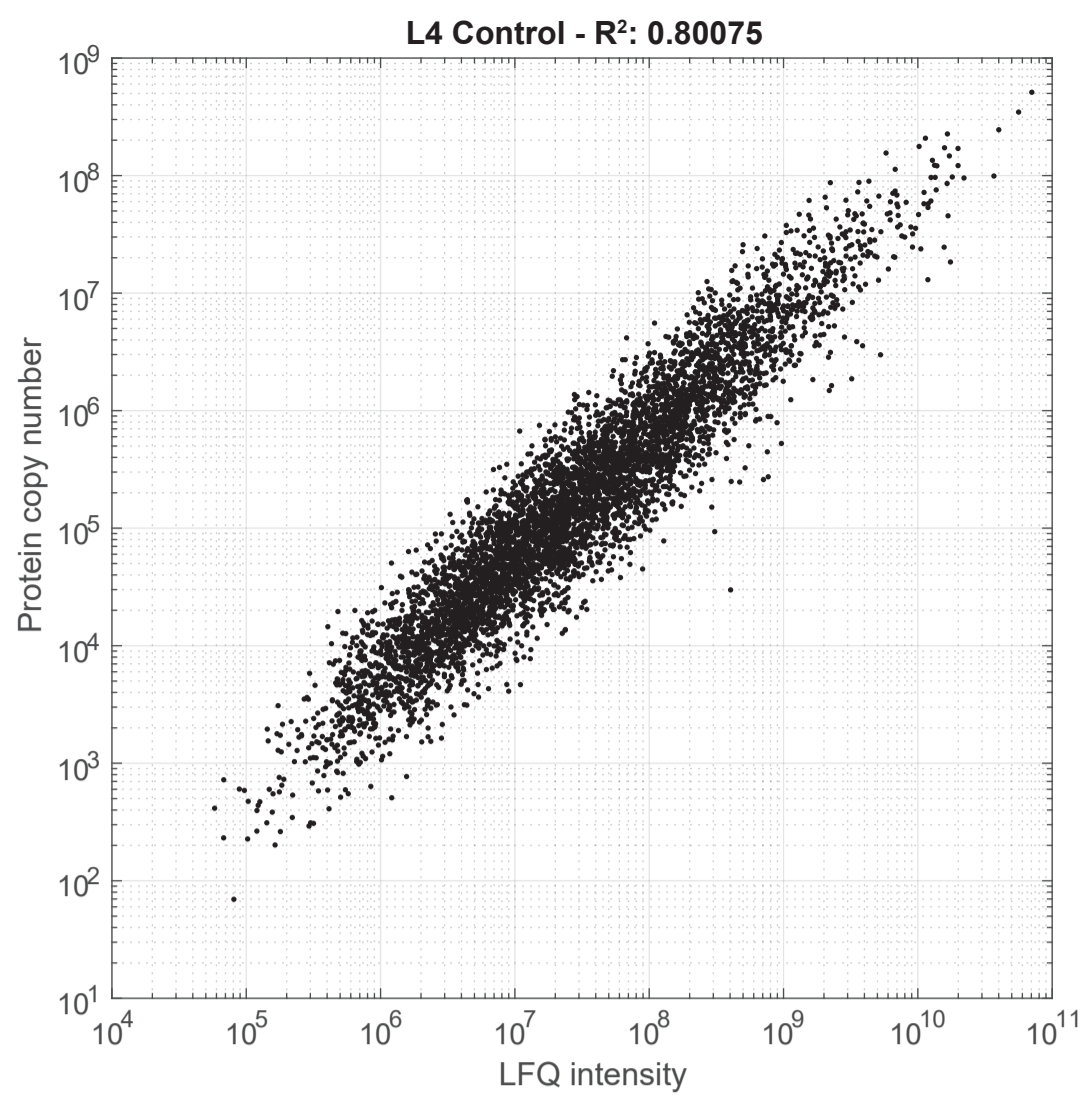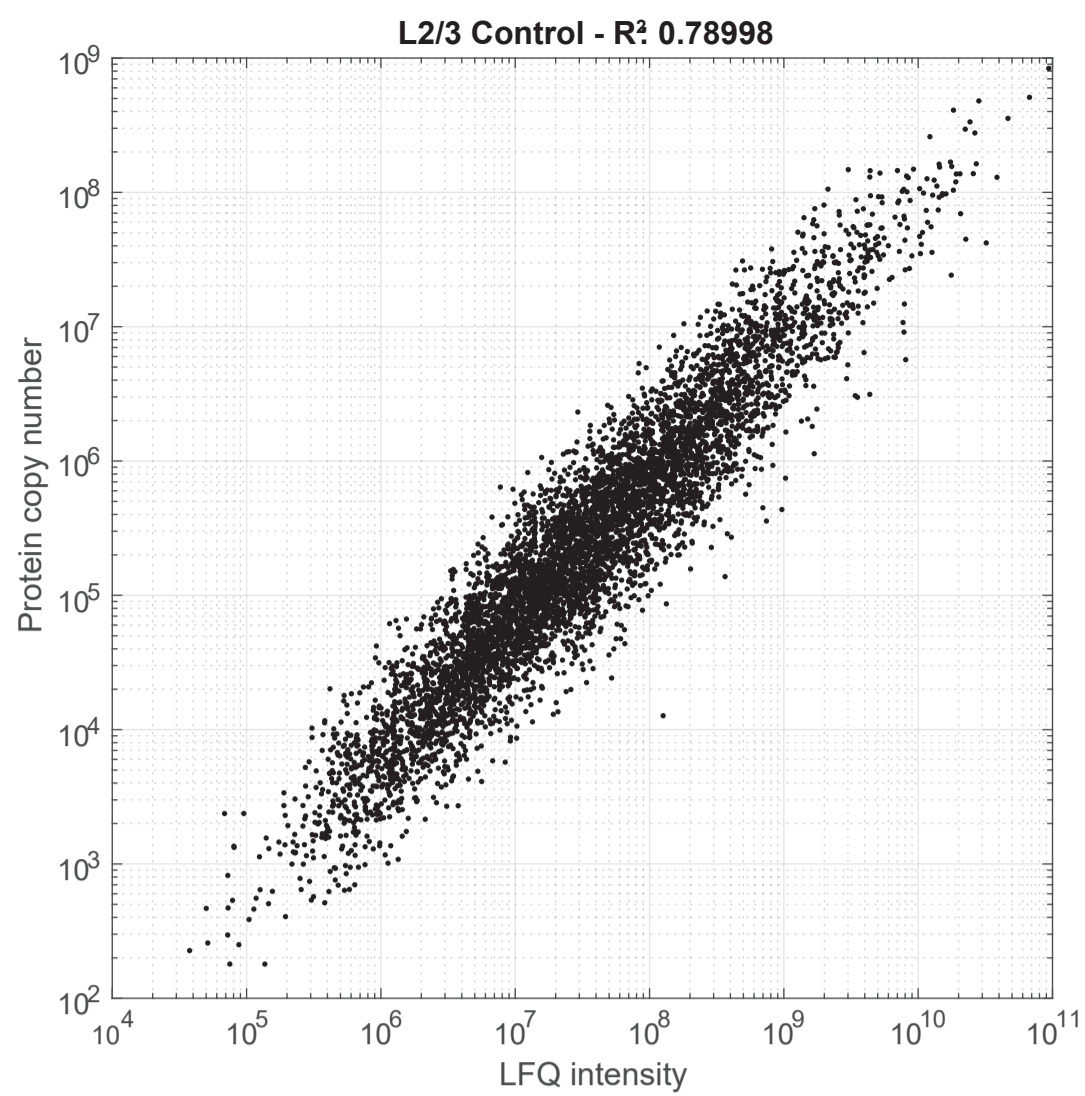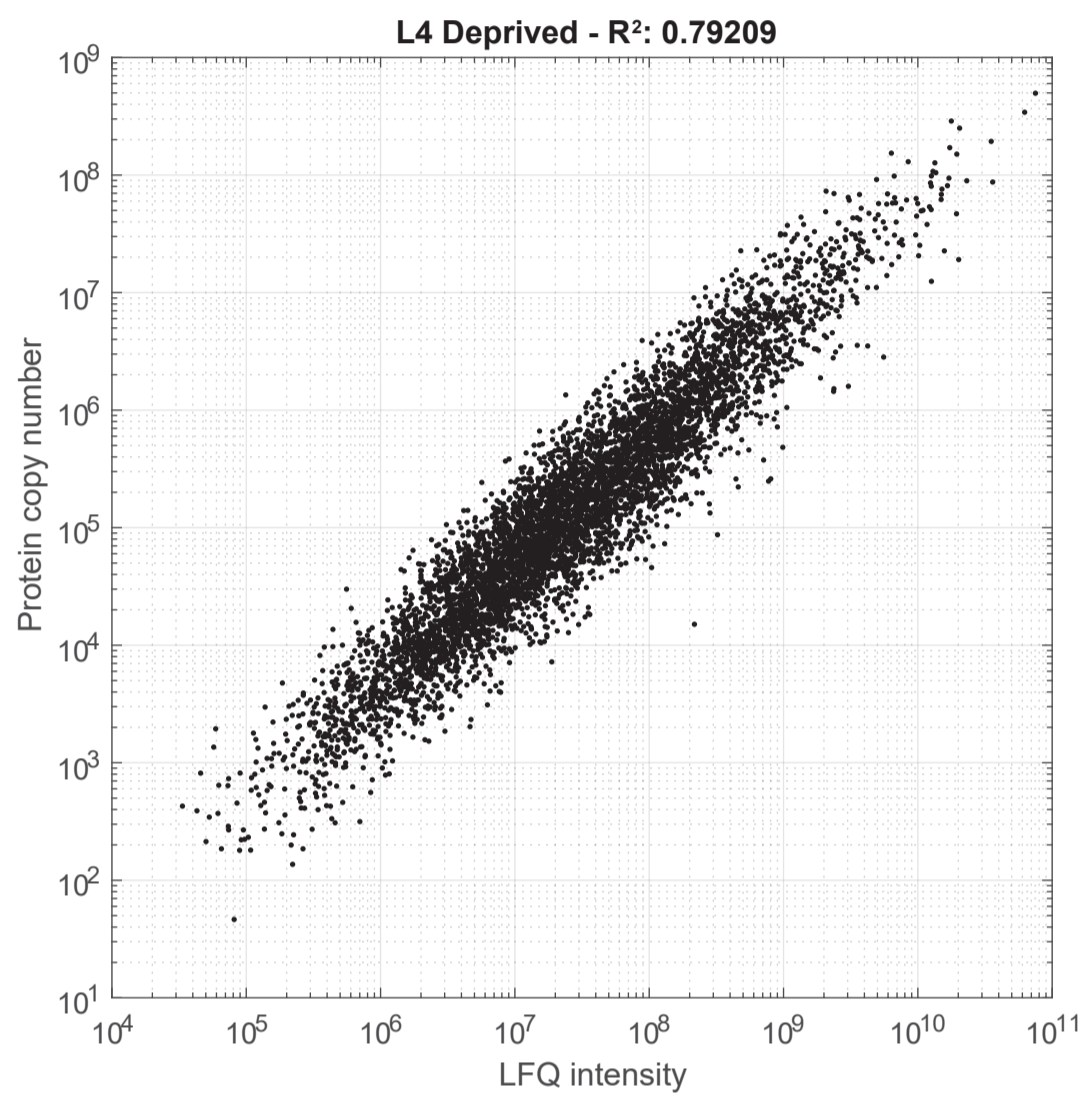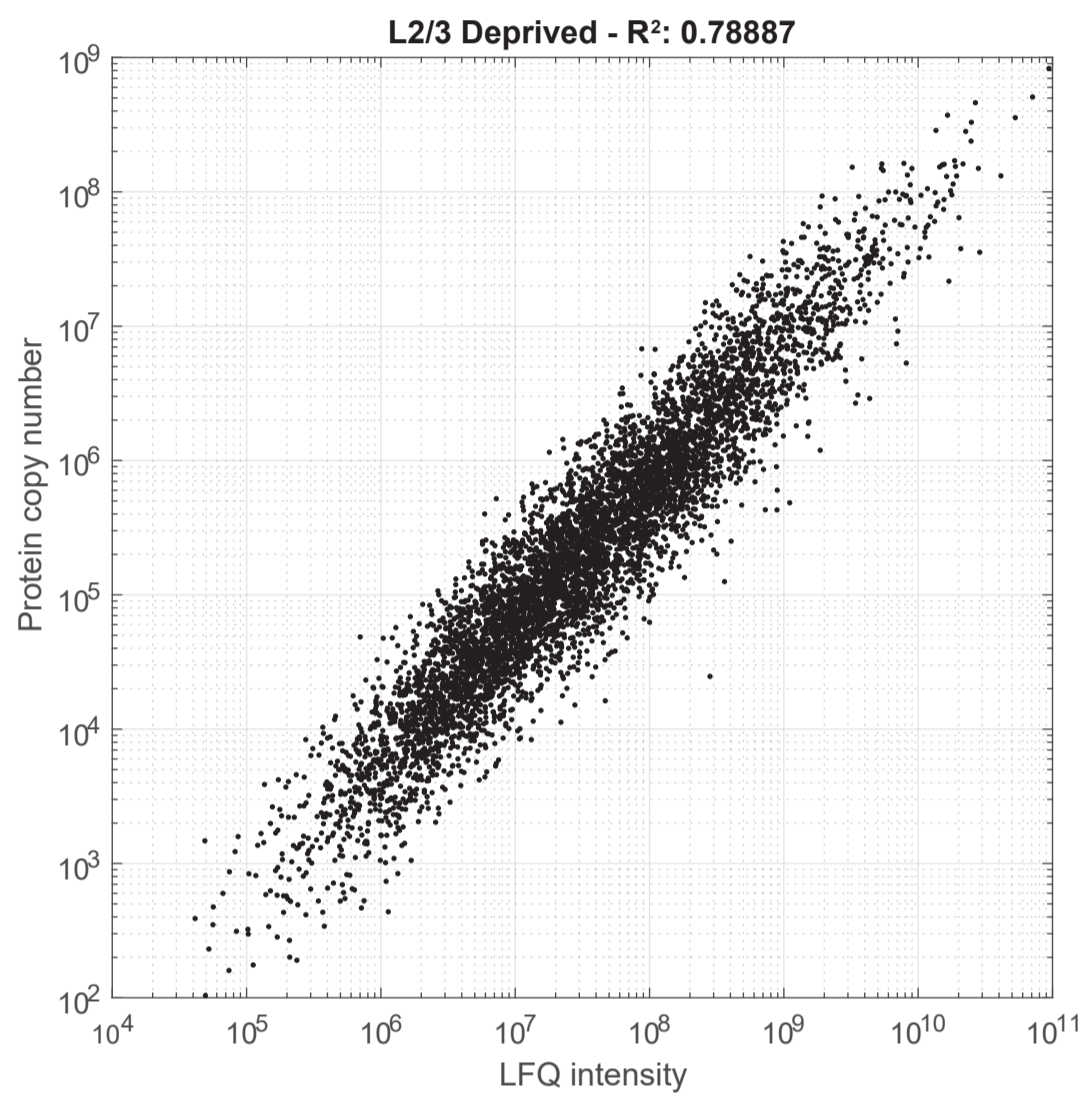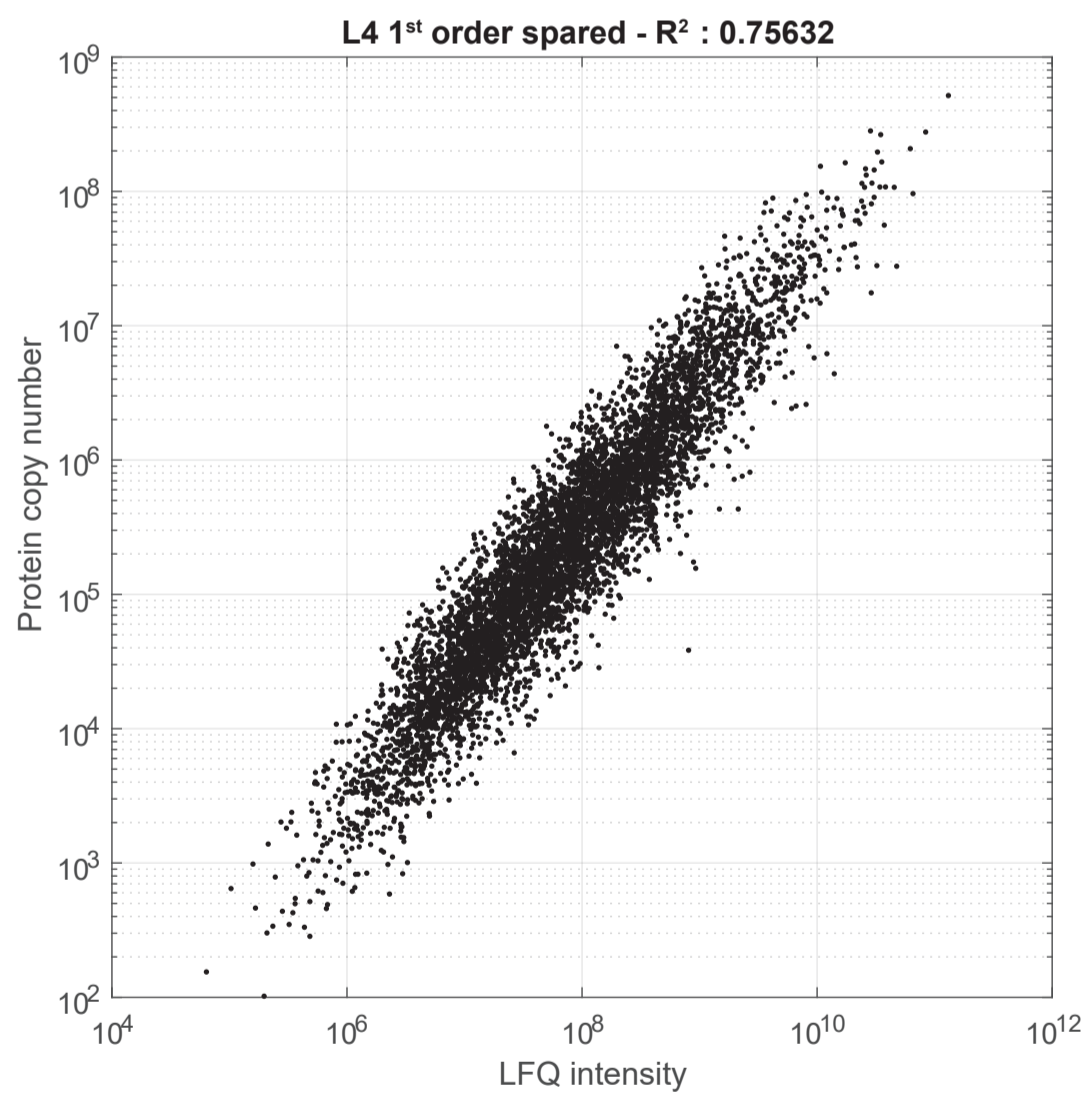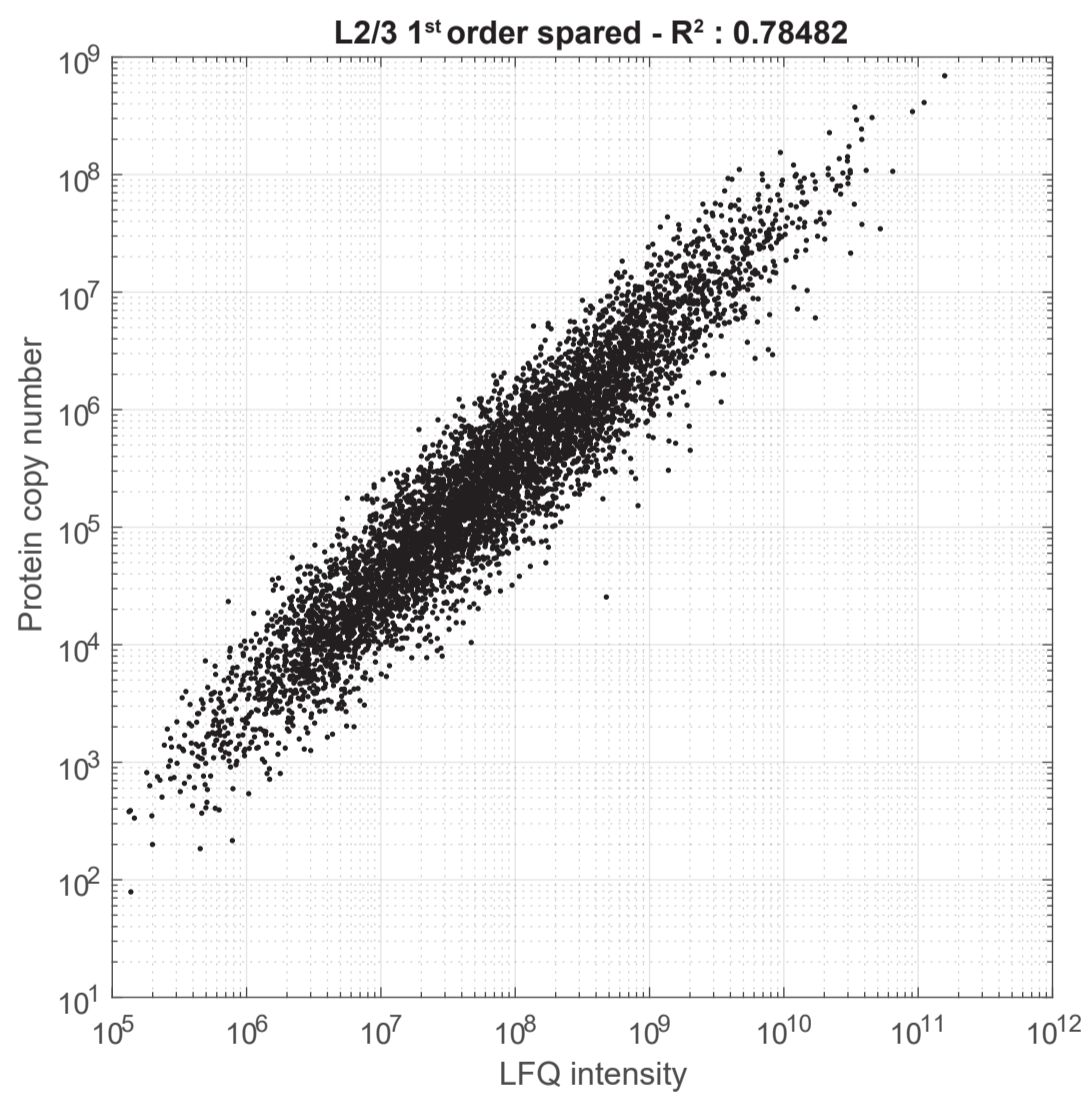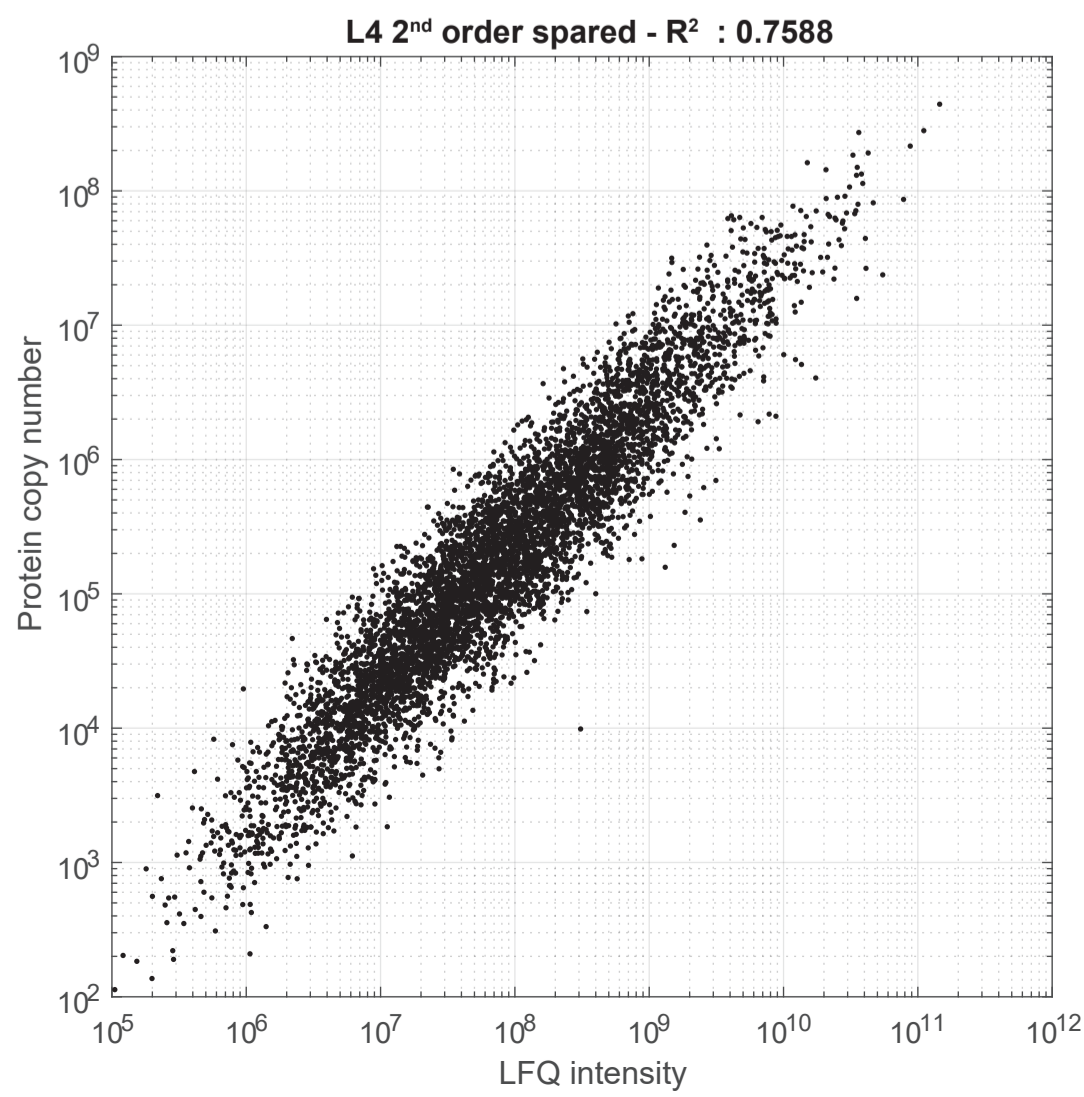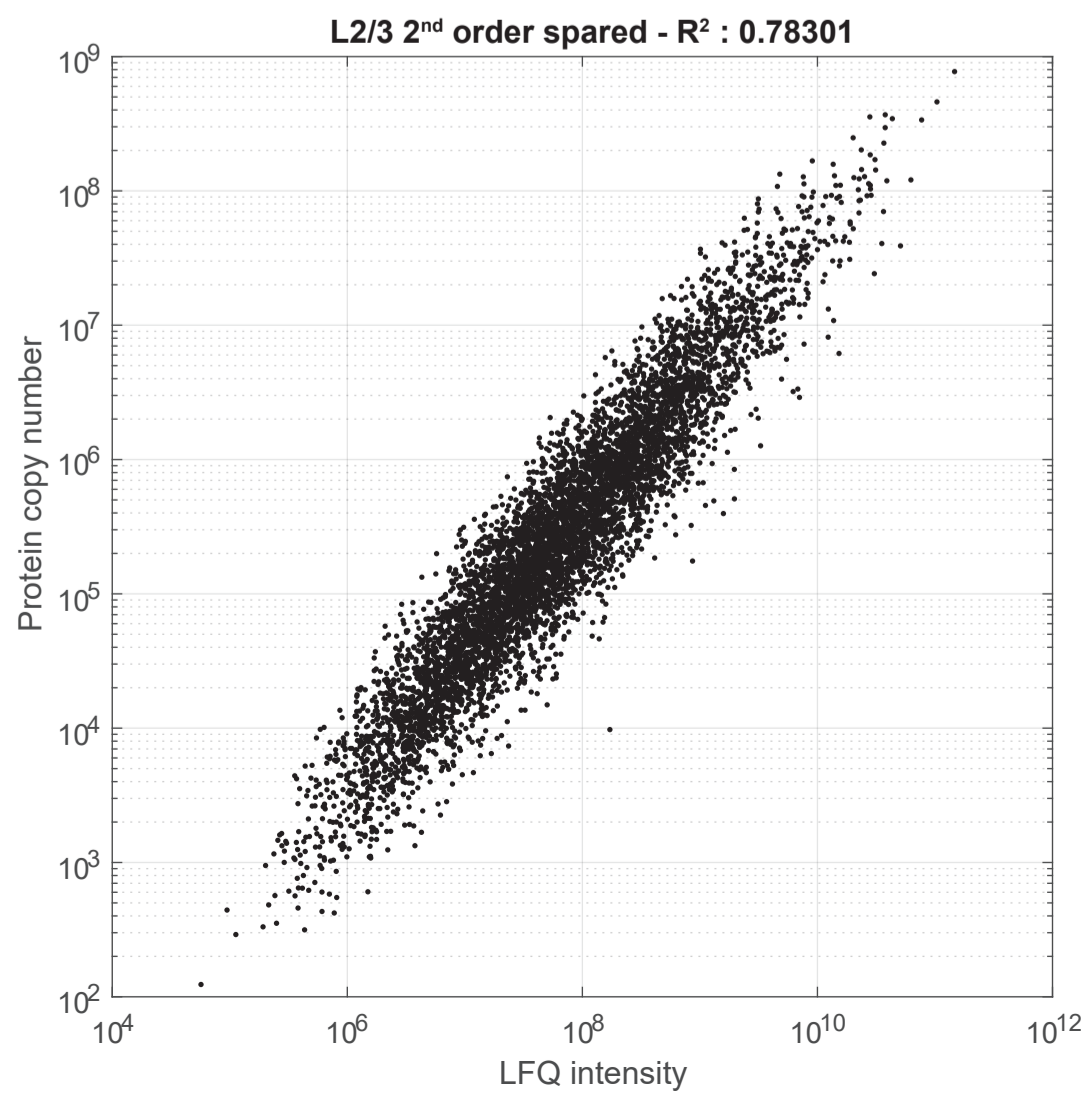

Supplement: Supplement materials [file gix082_Supp.zip › Supplemental_Figure1.pdf]

**A**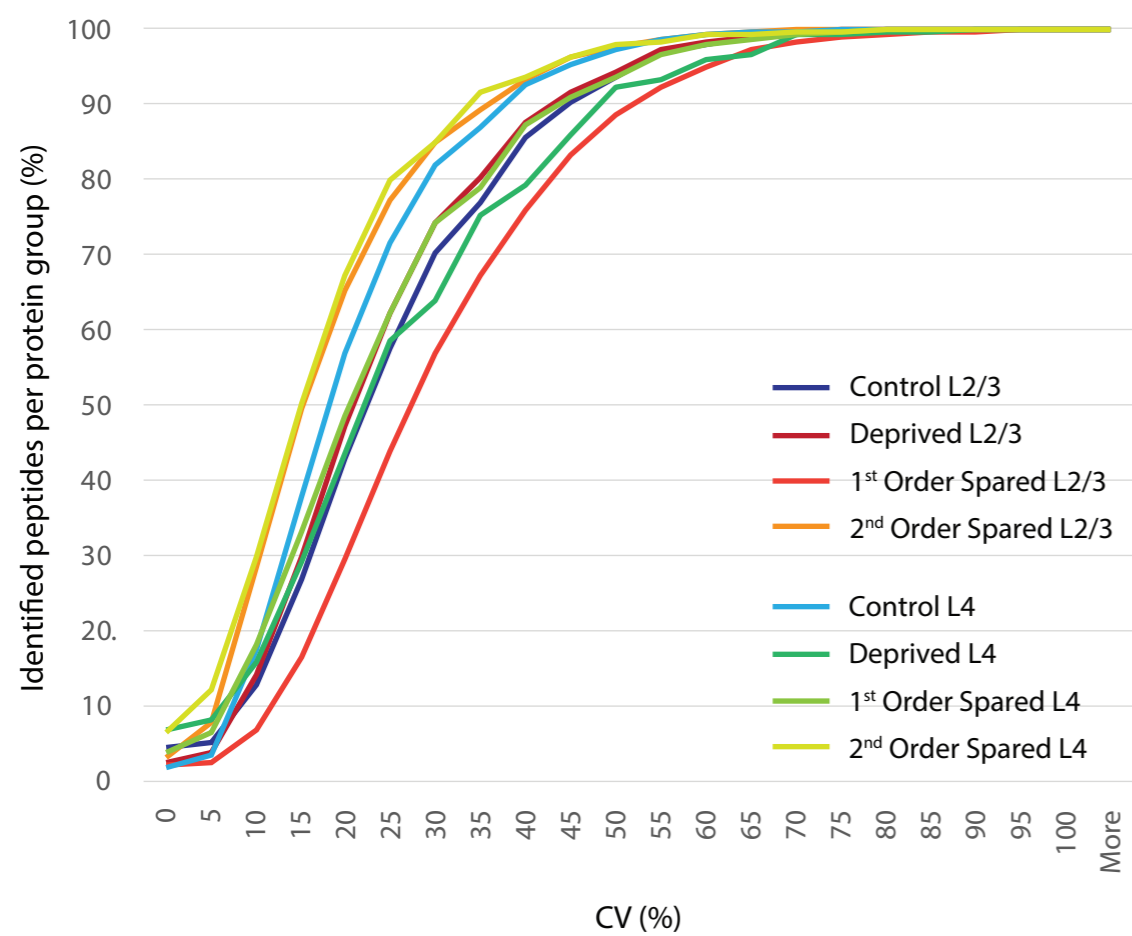**B**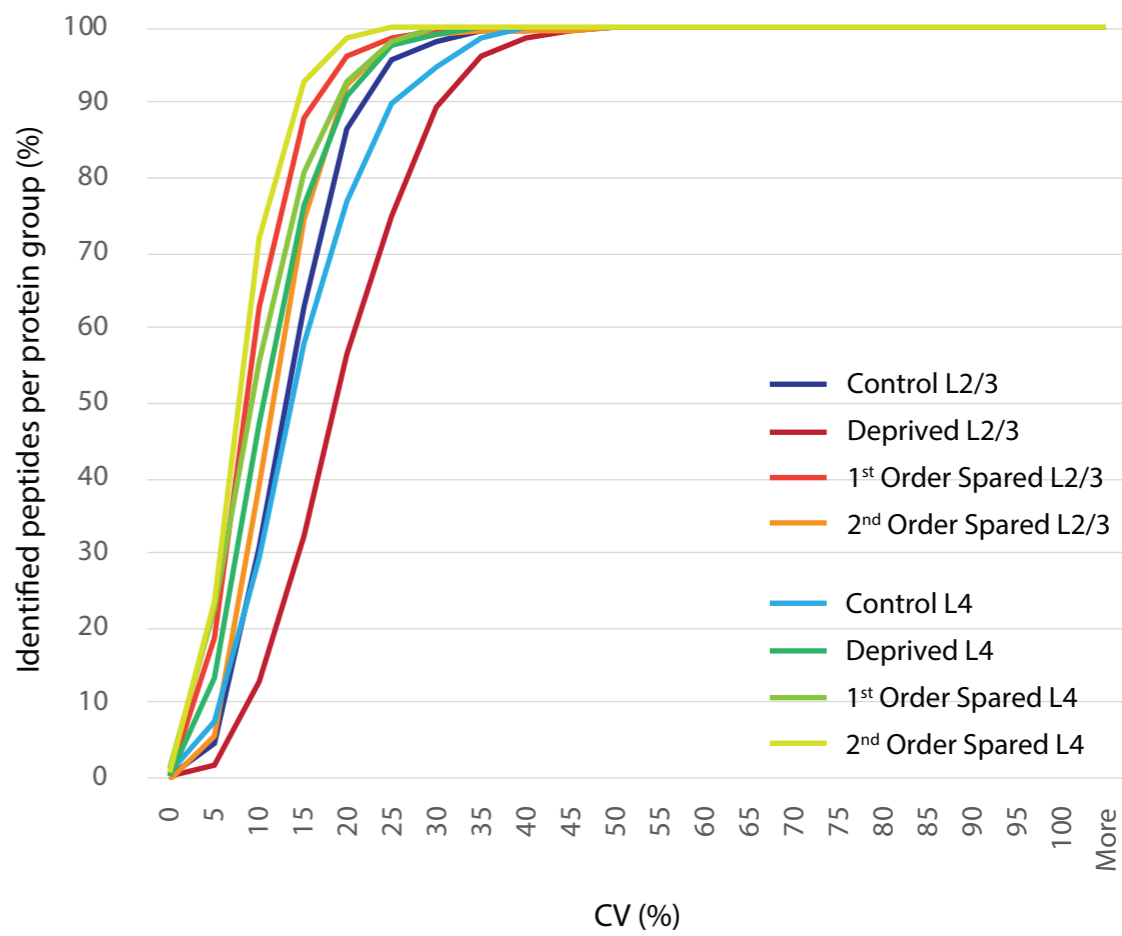**C**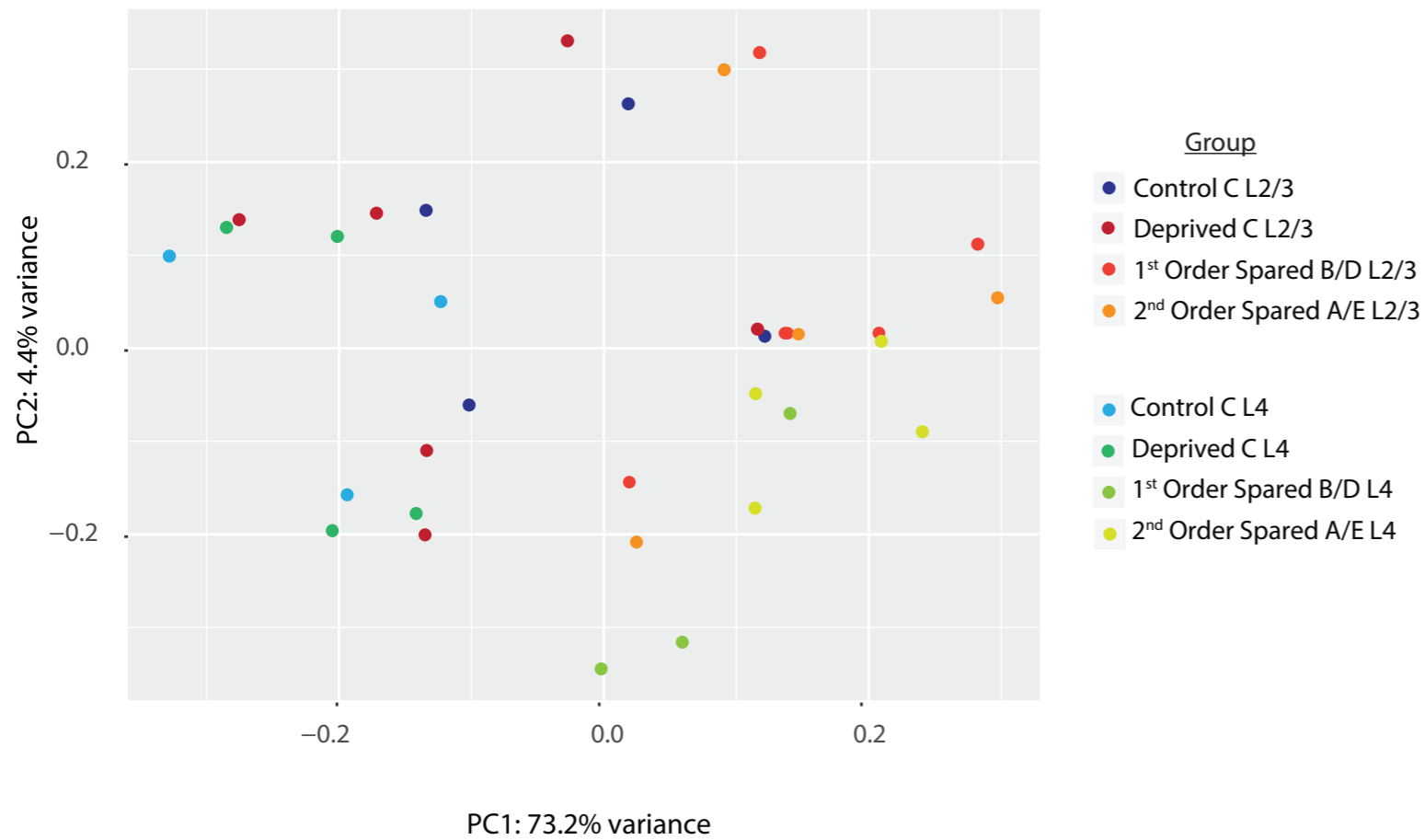**D**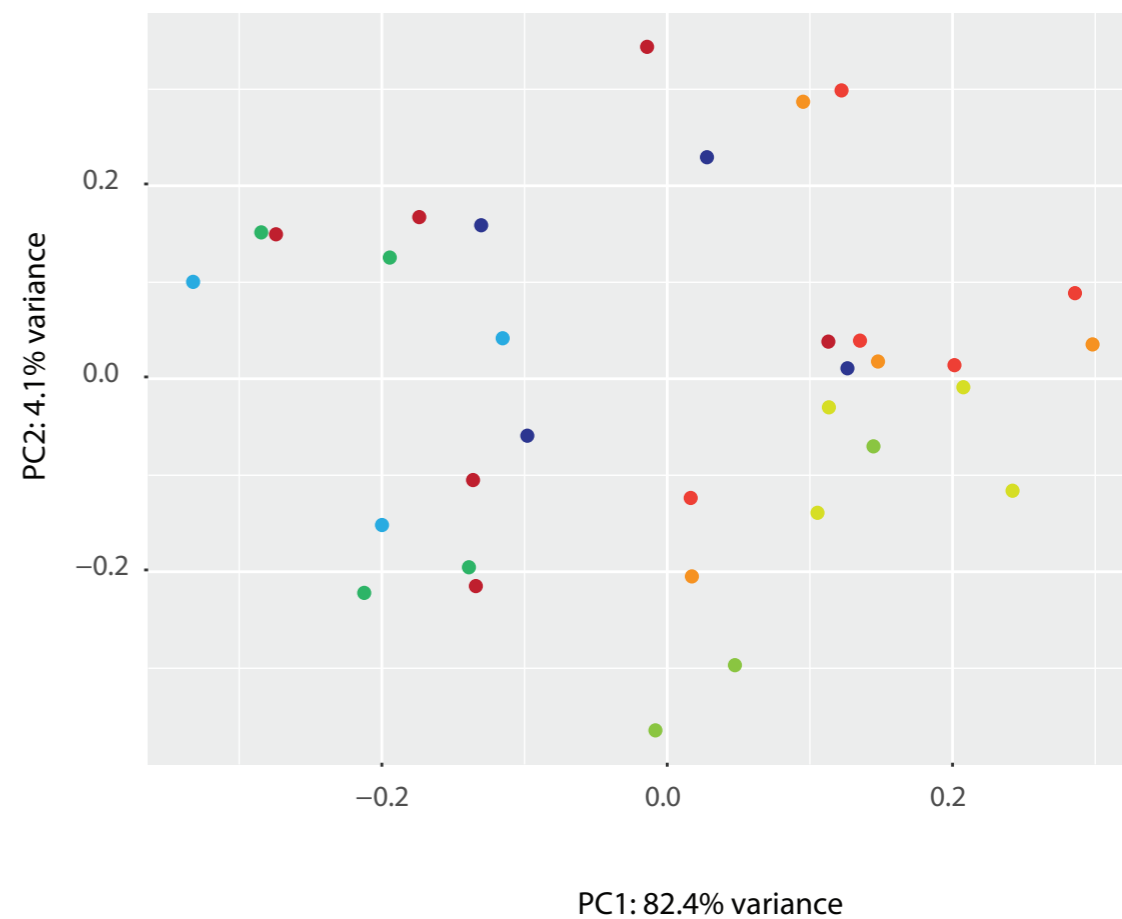**E**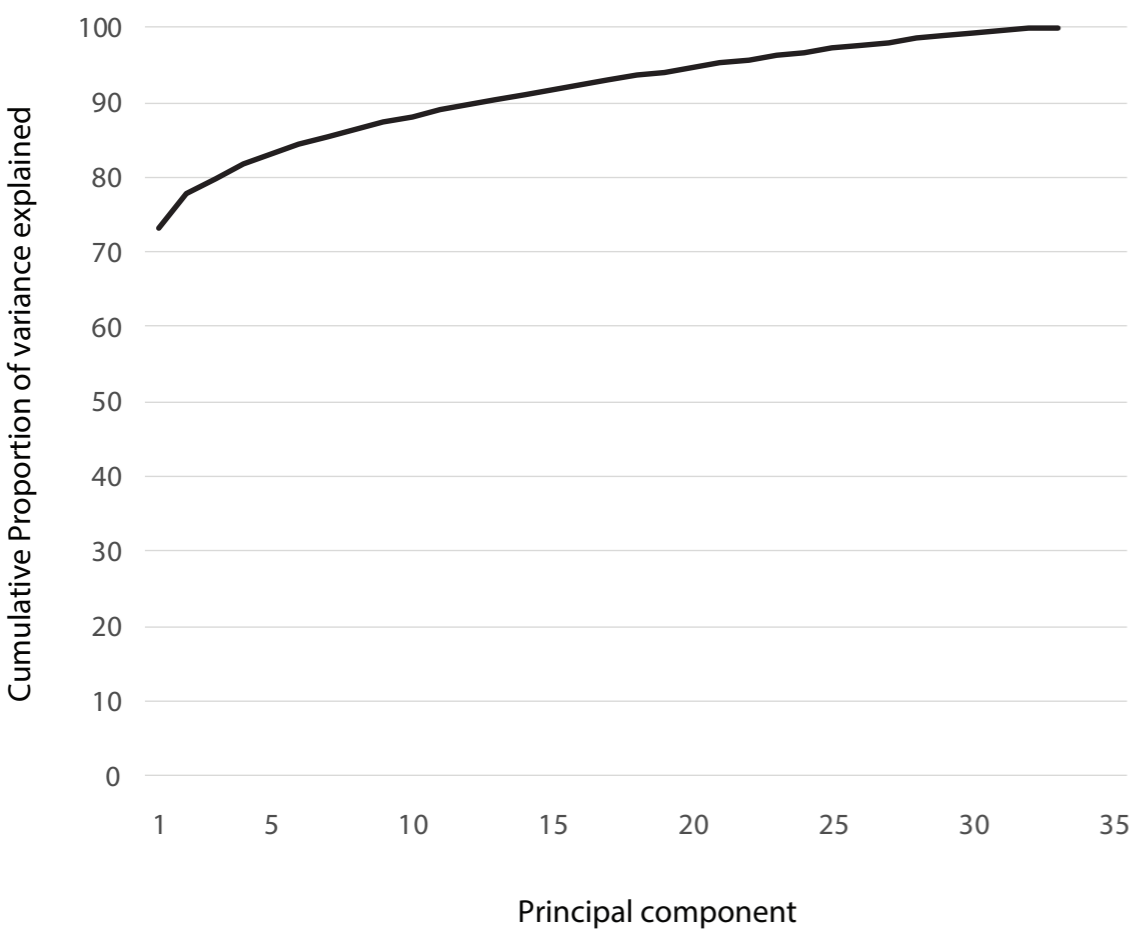**F**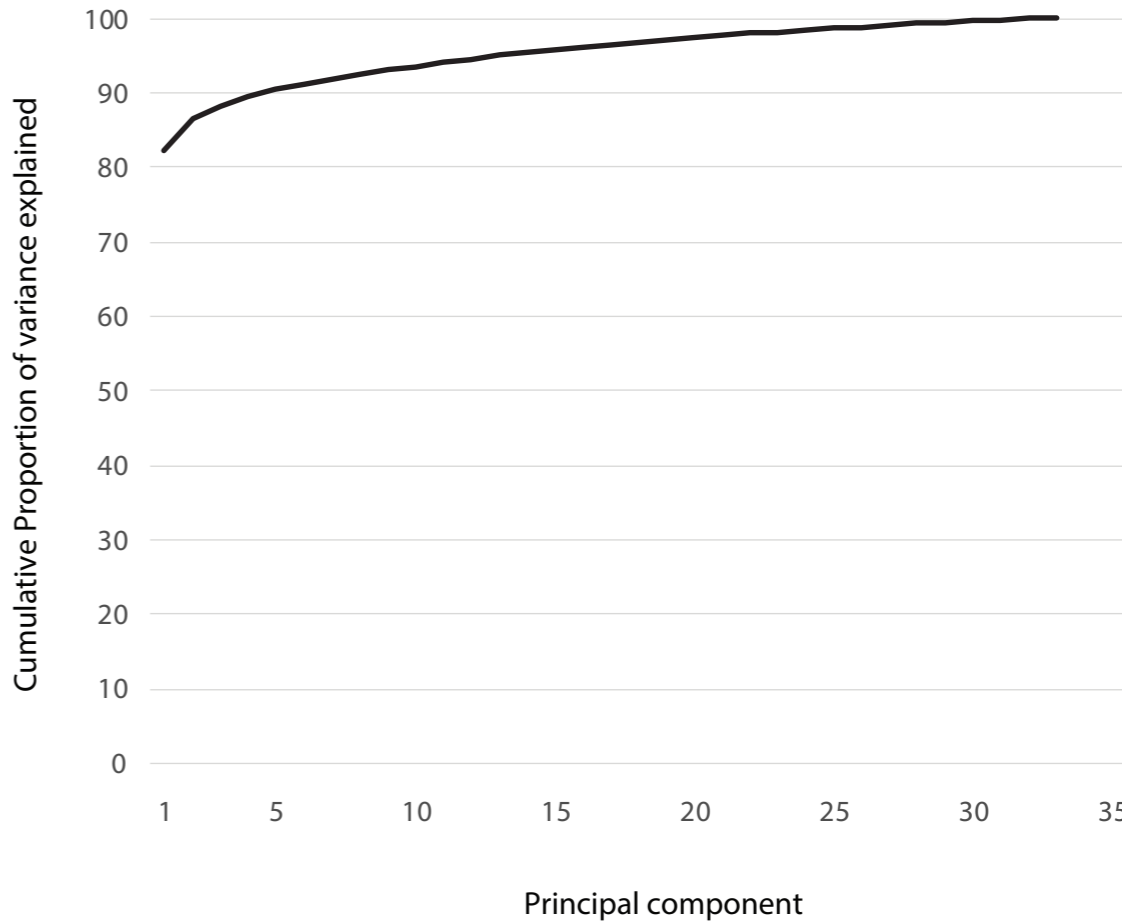

Supplement: Supplement materials [file gix082_Supp.zip › Supplemental_Figure2_Vertical.pdf]

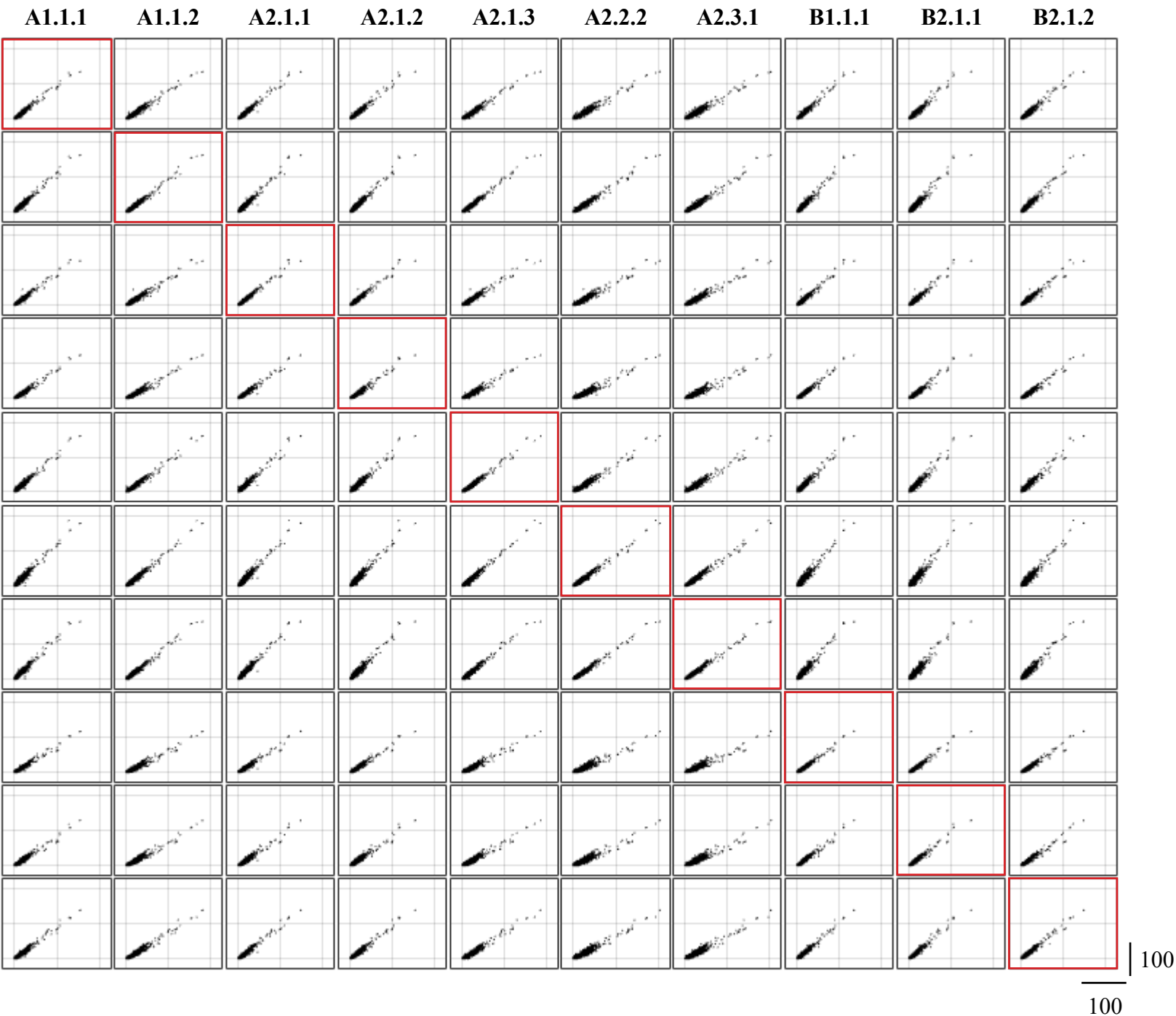

Supplement: Supplement materials [file gix082_Supp.zip › Supplemental_Figure3_Revised.pdf]

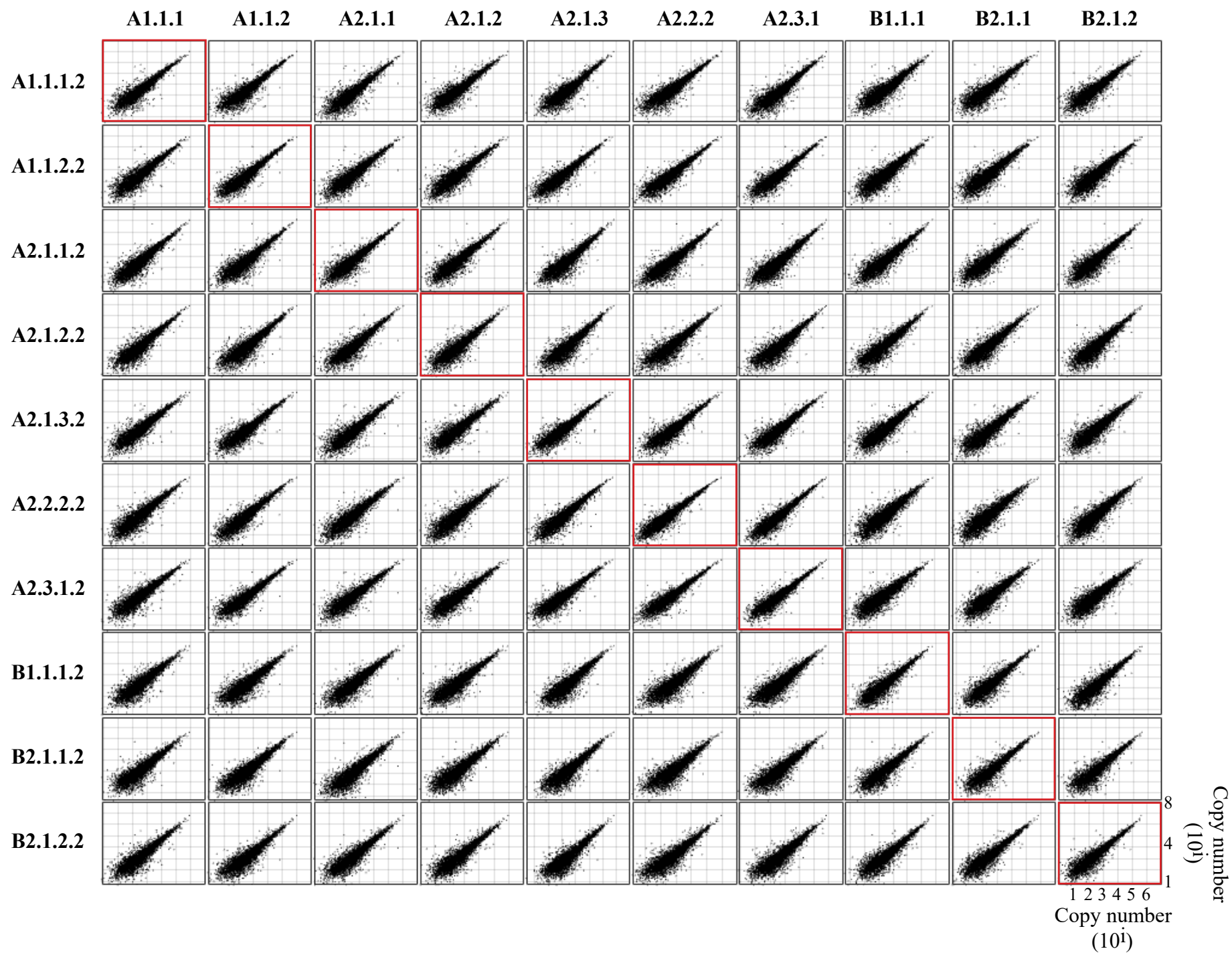

Supplement: Supplement materials [file gix082_Supp.zip › Supplemental_Figure4_Revised.pdf]
