# Supplementary material for: Proteomic landscape of the primary somatosensory cortex upon sensory deprivation
Source: Gigascience. 2017 Aug 23;6(10):1–10. doi: 10.1093/gigascience/gix082 (PMC5632293; doi:10.1093/gigascience/gix082)
Supplement: Response-to-Reviewer-Comments_Original-Submission.pdf [file gix082_Response-to-Reviewer-Comments_Original-Submission.pdf]

Author's Response To Reviewer Comments

Dear Dr. Nogoy,

We have now revised our manuscript, GIGA-D-17-00057, entitled “Proteomic landscape of the primary somatosensory cortex upon sensory deprivation”. Below we provide a point-by-point description of all the edits that we made in response to reviewers’ comments/requests. We would like to thank the editors for their insightful comments and constructive feedback. We believe that these edits have made the manuscript more accessible and will ensure that it can reach a larger audience. In addition, in this revision, we have included RRIDs wherever possible to improve reproducibility as per Gigascience policy. We hope that after these revisions the manuscript meets both the reviewer and editorial expectations. Thank you for your consideration in advance.

Sincerely,
Tansu Celikel, on behalf of the authors

[Reviewer #1 - R1]
In this Data Note Kole et al. present a nice proteomic analysis entitled "Proteomic landscape of the primary somatosensory cortex upon sensory deprivation" based on the widely used label free quantification within MaxQuant. Over all this is a nice analysis of how altered sensory input remodels the barrel cortex proteome. I found the analysis to be rigorous and in general very thorough however the presentation could be improved.
Short of a few minor improvements, i think this study should be accepted.

[R1] More instrument settings and operation details for the Fusion need to be included.
[Authors - AU] We have included a more detailed section on the Fusion in the Methods section.

[R1] P4 please add a comment mentioning that he deamidation, oxidation, and loss of ammonia may be occurring in vivo but more likely during the sample preparation steps.
[AU] This information has now been added in the Data validation and quality control section.

[R1] P5 please improve wording "decently correlated" is not very scientific. How about Showed a reasonable degree of correlation.
[AU] We agree that inclusion of a qualitative remark in this context is not an easy task. However, in order to help the novice readers (to the field) to interpret the degree of correlation, we find it useful to include a qualitative descriptor. Based on the reviewer’s suggestion, we have edited the aforementioned sentence, which now reads:
"The correlation (R2) between the two quantification methods ranged from 0.76 to 0.80, suggesting good consensus of protein abundance estimation.”

[R1] Does Figure 4 need a title? [Added] the legend is a bit funky
[AU] The title ‘Protein quantification is independent from peptide mass or length’ has been added to Figure 4. Furthermore, we have edited the legend of the Figure 4 to improve the readability.

[R1] Figure 5 PCA details and conclusions need to be improved, figure is a bit confusing, can it

be reduced to 1 or 2 panels? The text for the PCA analysis should also be clarified.
[AU] Panels A-D of the original Figure 5 have been moved to the Supplemental Material. To provide more details on the PCA, a single panel was added to the new Figure 5 containing a cumulative plot of the percent explained variance of each principal component. In addition, we now provide examples of the command lines that were used to obtain the data presented in Figure 5 and the associated supplemental figure as a supplemental material.

[R1] In general many of the labels in the figures should be improved so at first glance the reader can follow for example -- Figure 3 labels are a bit un-intuitive. what exactly does 1st Order Spared mean?
[AU] To improve clarity, the Figure 3 legend now includes a description of the axis labels as well as a reference to Figure 1, where the nomenclature is described in detail. In short, 1st order spared refers to the experimental group where the sample comes from cortical columns neighboring the deprived whisker column. Because our experimental design includes only a single row whisker deprivation all neighboring whiskers were intact, i.e. spared. In addition, we have included the following brief statement in the methods section to independently attract the attention of the reader to the nomenclature:
“ In the barrel cortex, cortical columns can be grouped by their relative distance to each other. Cortical columns B and D, for example, are named as the 1st order neighboring cortical columns in respect to the C row column. Similarly A and E row columns constitute the 2nd order neighboring columns.”

[R1] In the discussion, a sentence or 2 should be added to mention the significant limitation that the apparent protein level could arise from averaging signals from different cells.
[AU] The Re-use potential section has been expanded with the following to accommodate this important point:
“It should be noted however that the collected samples contain the entirety of the cellular population, i.e. are not cell type-specific. Signals originating from all cell types are thus averaged, which should be considered by researchers reusing this dataset.”

[R1] There are a lot of figure panels here, if possible please try to focus the scope of the figures a bit and move any supportive figures to the supp in order to keep the main text punchy.
[AU] As mentioned above, we have substantially edited the Figure 5 and reduced the number of panels therein. We feel, however, that the remaining figures are key for readers to assess the quality of the dataset and have therefore not been moved into Supplementary Materials.

[Reviewer #2 – R2]
The article from Kole and co-authors entitled 'Proteomic landscape of the primary somatosensory cortex upon sensory deprivation' describes a layer-and-column specific proteomic profiling of the barrel cortex with the goal to elucidate the molecular mechanisms involved in experience-dependant plasticity affecting neural circuits. This article addresses a key need in the field of brain proteomics, which is the analysis of microscopic and functionally isolated brain regions, to ultimately cope with the high level of brain cellular and molecular complexity. The authors have done a very exhaustive work to show that they are able to successfully apply mass-spectrometry based methods to investigate microscopic brain regions, obtaining sample protein

coverage comparable to present day proteomics standards and high reproducibility between biological and technical replicas. From a technical perspective this is thus an important contribution to the field. The absence of biological data (which is not required for Data Note Articles) does not allow clarifying if this work will also represent an important biological contribution.
Nevertheless, several issues should be addressed prior to publication:

[R2] It is my understanding that the dissection method used to isolate L4 from L2/3 in a column-specific manner is quite new. The authors should better describe how they do it, or give appropriate references. Particularly relevant would be to explain how do they make sure that they can collect L4 separately from L2/3.
[AU] The method we have employed is indeed novel although it is based on acute slice preparations commonly used for in vitro recordings from the barrel cortex. We have originally developed the laminar and columnar isolation protocol in a separate manuscript which is currently under review for publication. In that manuscript we employ RNA-seq to address the experience dependent transcriptomic changes observed in the barrel cortex in columnar and layer resolution. Although we have cited this manuscript to redirect those readers who are interested in replicating the work-flow for the sample isolation, we of course agree that the methods should be clear independent from any citation to prior work. Thus we have included the following statement under the “slice preparation and sample collection” subheading :
“For sample isolation, slices were placed under a microscope equipped with Dodt gradient contrast, used for visualization of the granular segments of the live neocortical tissue, such as the L4 in the barrel cortex. Visualized cortical columns (A-E) were separated from each other using a pulled pipette (Sutter Instruments P-2000), tip size of ~5 micrometers, serving as a microneedle. Layers (L) 2/3 and L4 were isolated based on the established contrast criteria commonly used in electrophysiological analysis of barrel cortical neurons in acute slices (Celikel et al. 2004; Allen et al. 2003).”

[R2] Could the authors get 3 biological replicas for all samples? For instance in control L2/3 and L4, there are only 2 biological replicas, no? For other samples there seems to be 4 biological replicas (L4 2nd order), but only three female pups are said to be used for each group in this work. (?). The methods section should be re-written to accommodate all these discrepancies. Actually, extending the methods section to explain in more detail the samples gathered and analysed would help the reader. Similarly an extra panel could be added to figure 1 to show this in a clear and schematic manner.
[AU] As the reviewer correctly noted, control L2/3 and L4 have two biological replicas; the remaining samples in these groups are indeed technical replicas. The sample numbers and grouping information in Figure 1 aim to communicate the number of biological and technical replicas across all groups. In this revised version of the manuscript, we are also providing an additional Supplemental table (Supplemental Table 1) to further clarify the sample origin (using Mouse ID). As requested we have also edited the method section to better explain the sample collection, handling and analysis procedures.
We would like to also note that we have performed extensive analysis, as detailed in the Figure 6, Supplemental Figure 2 and 3, that provide quantitative information about peptide count and protein copy number correlations between and across biological samples and their technical replicas. These analysis showed that independent from whether technical replicas are compared

to their original sample (Figure 6), or other samples within the same experimental group (Supplemental Figures 2,3) there is a strong correlation between the reads (R-squared >0.89-96). Re: the use of only female pups. Although we have collected data from both male and female mice, we have performed mass-spectroscopic analysis of only female mice as the sample yield was more favorable in this group. Because the animals were pups, i.e. not having reached their sexual maturity, inclusion of samples from a single sex enabled us to control for the confounding variable of sex.

[R2] The authors briefly refer to the low level of protein contaminants (page 4 lane 51) found in their preparations. What do they refer to,what are contamiant proteins? Please further develop and give some figures. i.e. what is the fraction of contaminant proteins.
[AU] During sample preparation contaminant proteins such as keratins and trypsin can enter the processed samples. The Methods section has been expanded with a brief explanation of what is meant by contaminant proteins and how they were identified. Additionally, the Data validation and quality control section has been expanded with the minimum, first quartile, median, mean, third quartile and maximum numbers of contaminants.
Minor points

[R2] Have the authors only used somatosensory column C for controls? If so please clarify in the text.
[AU] This is indeed the case, and has been added to the Methods section

[R2] Figure 1B last step, please change 'MS Analysis' by 'LC-MS analysis'.
[AU] The label is edited as requested.

[R2] Page 6 lane 11 , change 'decently' for a more appropriate word.
[AU] This was also noted by the Reviewer #1. As noted above (Comment #3, R1): We agree that inclusion of a qualitative remark in this context is not an easy task. However, in order to help the novice readers (to the field) to interpret the degree of correlation, we find it useful to include a qualitative descriptor. Based on the reviewer's suggestion, we have edited the aforementioned sentence, which now reads:
"The correlation (R2) between the two quantification methods ranged from 0.76 to 0.80, suggesting good consensus of protein abundance estimation."
