# Supplementary material for: Proteomic landscape of the primary somatosensory cortex upon sensory deprivation
Source: Gigascience. 2017 Aug 23;6(10):1–10. doi: 10.1093/gigascience/gix082 (PMC5632293; doi:10.1093/gigascience/gix082)
Supplement: Reviewer-1-Report-(Original-Submission).pdf [file gix082_Reviewer-1-Report-(Original-Submission).pdf]

Reviewer Report

Title: Proteomic landscape of the primary somatosensory cortex upon sensory deprivation

Version: Original Submission **Date:** 3/28/2017

Reviewer name: Jeffrey Savas

Reviewer Comments to Author:

In this Data Note Kole et al. present a nice proteomic analysis entitled "Proteomic landscape of the primary somatosensory cortex upon sensory deprivation" based on the widely used label free quantification within MaxQuant. Over all this is a nice analysis of how altered sensory input remodels the barrel cortex proteome. I found the analysis to be rigorous and in general very thorough however the presentation could be improved.Short of a few minor improvements, i think this study should be accepted.1. More instrument settings and operation details for the Fusion need to be included.2. P4 please add a comment mentioning that he deamidation, oxidation, and loss of ammonia may be occurring in vivo but more likely during the sample preparation steps.3. P5 please improve wording "decently correlated" is not very scientific. How about Showed a reasonable degree of correlation.4. Does Figure 4 need a title? the legend is a bit funky5. Figure 5 PCA details and conclusions need to be improved, figure is a bit confusing, can it be reduced to 1 or 2 panels? The text for the PCA analysis should also be clarified.6. In general many of the labels in the figures should be improved so at first glance the reader can follow for example -- Figure 3 labels are a bit un-intuitive. what exactly does 1st Order Spared mean?7. In the discussion, a sentence or 2 should be added to mention the significant limitation that the apparent protein level could arise from averaging signals from different cells.8. There are a lot of figure panels here, if possible please try to focus the scope of the figures a bit and move any supportive figures to the supp in order to keep the main text punchy.

Level of Interest

Please indicate how interesting you found the manuscript: An article of importance in its field

Quality of Written English

Please indicate the quality of language in the manuscript: Needs some language corrections before being published

Declaration of Competing Interests

Please complete a declaration of competing interests, considering the following questions:

- Have you in the past five years received reimbursements, fees, funding, or salary from an organisation that may in any way gain or lose financially from the publication of this manuscript, either now or in the future?

- Do you hold any stocks or shares in an organisation that may in any way gain or lose financially from the publication of this manuscript, either now or in the future?
- Do you hold or are you currently applying for any patents relating to the content of the manuscript?
- Have you received reimbursements, fees, funding, or salary from an organization that holds or has applied for patents relating to the content of the manuscript?
- Do you have any other financial competing interests?
- Do you have any non-financial competing interests in relation to this paper?

If you can answer no to all of the above, write 'I declare that I have no competing interests' below. If your reply is yes to any, please give details below.

I declare that I have no competing interests

I agree to the open peer review policy of the journal. I understand that my name will be included on my report to the authors and, if the manuscript is accepted for publication, my named report including any attachments I upload will be posted on the website along with the authors' responses. I agree for my report to be made available under an Open Access Creative Commons CC-BY license (http://creativecommons.org/licenses/by/4.0/). I understand that any comments which I do not wish to be included in my named report can be included as confidential comments to the editors, which will not be published.

I agree to the open peer review policy of the journal
