# Supplementary material for: Proteomic landscape of the primary somatosensory cortex upon sensory deprivation
Source: Gigascience. 2017 Aug 23;6(10):1–10. doi: 10.1093/gigascience/gix082 (PMC5632293; doi:10.1093/gigascience/gix082)
Supplement: Reviewer-2-Report-(Original-Submission).pdf [file gix082_Reviewer-2-Report-(Original-Submission).pdf]

Reviewer Report

Title: Proteomic landscape of the primary somatosensory cortex upon sensory deprivation

Version: Original Submission **Date:** 5/9/2017

Reviewer name: Alex Bayes

Reviewer Comments to Author:

The article from Kole and co-authors entitled 'Proteomic landscape of the primary somatosensory cortex upon sensory deprivation' describes a layer-and-column specific proteomic profiling of the barrel cortex with the goal to elucidate the molecular mechanisms involved in experience-dependant plasticity affecting neural circuits. This article addresses a key need in the field of brain proteomics, which is the analysis of microscopic and functionally isolated brain regions, to ultimately cope with the high level of brain cellular and molecular complexity. The authors have done a very exhaustive work to show that they are able to successfully apply mass-spectrometry based methods to investigate microscopic brain regions, obtaining sample protein coverage comparable to present day proteomics standards and high reproducibility between biological and technical replicas. From a technical perspective this is thus an important contribution to the field. The absence of biological data (which is not required for Data Note Articles) does not allow clarifying if this work will also represent an important biological contribution.Nevertheless, several issues should be addressed prior to publication:Major points1. It is my understanding that the dissection method used to isolate L4 from L2/3 in a column-specific manner is quite new. The authors should better describe how they do it, or give appropriate references. Particularly relevant would be to explain how do they make sure that they can collect L4 separately from L2/3.2. Could the authors get 3 biological replicas for all samples? For instance in control L2/3 and L4, there are only 2 biological replicas, no? For other samples there seems to be 4 biological replicas (L4 2nd order), but only three female pups are said to be used for each group in this work. (?). The methods section should be re-written to accommodate all these discrepancies. Actually, extending the methods section to explain in more detail the samples gathered and analysed would help the reader. Similarly an extra panel could be added to figure 1 to show this in a clear and schematic manner.3. The authors briefly refer to the low level of protein contaminants (page 4 lane 51) found in their preparations. What do they refer to,wha tare contamiant proteins? Please further develop and give some figures. i.e. what is the fraction of contaminant proteins.Minor pointsHave the authors only used somatosensory column C for controls? If so please clarify in the text.Figure 1B last step, please change 'MS Analysis' by 'LC-MS analysis'.Page 6 lane 11 , change 'decently' for a more appropriate word.

Level of Interest

Please indicate how interesting you found the manuscript: An exceptional article

Quality of Written English

Please indicate the quality of language in the manuscript: Acceptable

Declaration of Competing Interests

Please complete a declaration of competing interests, considering the following questions:

- Have you in the past five years received reimbursements, fees, funding, or salary from an organisation that may in any way gain or lose financially from the publication of this manuscript, either now or in the future?
- Do you hold any stocks or shares in an organisation that may in any way gain or lose financially from the publication of this manuscript, either now or in the future?
- Do you hold or are you currently applying for any patents relating to the content of the manuscript?
- Have you received reimbursements, fees, funding, or salary from an organization that holds or has applied for patents relating to the content of the manuscript?
- Do you have any other financial competing interests?
- Do you have any non-financial competing interests in relation to this paper?

If you can answer no to all of the above, write 'I declare that I have no competing interests' below. If your reply is yes to any, please give details below.

I declare that I have no competing interests

I agree to the open peer review policy of the journal. I understand that my name will be included on my report to the authors and, if the manuscript is accepted for publication, my named report including any attachments I upload will be posted on the website along with the authors' responses. I agree for my report to be made available under an Open Access Creative Commons CC-BY license (http://creativecommons.org/licenses/by/4.0/). I understand that any comments which I do not wish to be included in my named report can be included as confidential comments to the editors, which will not be published.

I agree to the open peer review policy of the journal
