# Supplementary material for: Proteomic landscape of the primary somatosensory cortex upon sensory deprivation
Source: Gigascience. 2017 Aug 23;6(10):1–10. doi: 10.1093/gigascience/gix082 (PMC5632293; doi:10.1093/gigascience/gix082)
Supplement: Supplement materials [file gix082_Supp.zip › Supplemental Figure Table Legends.doc]

**Supplemental Figure 1. Correlation between LFQ and protein copy numbers.** Scatter plots of LFQ values (x axis) and inferred protein copy numbers [6] (y axis), showing a linear correlation between the two quantification methods (R^2^ > 0.75).

**Supplemental Figure 2. Variance quantification of individual samples.** **(A, B)** Cumulative plots of the coefficient of variance (CV) in the number of identified peptides in each experimental group. Including proteins with at least one identified peptide (A), CVs of 30% or less are found in ~74%. With an increased cut-off (10 peptides) ~70% of proteins show a CV of 15% or less (C).

**(C, D)** Principal component analysis (PCA) using numbers of identified peptides per protein. With a cut-off of 1 identified peptide, ~78% of variance is explained by Principal Component

(PC) 1 and 2 (B); this is ~87% when a cut-off of 10 identified peptides is used (D). **(E, F)** Cumulative plots of showing the percent variance explained by each PC. With a cutoff of 1 identified peptide (C) the first 5 PCs explain ~83% of the variance; using a cutoff of 10 peptides (F) this is ~91%.

**Supplemental Figure 3. Distribution of peptides per protein group in biological and technical replicates.** Scatter plots of identified peptides per protein group from biological and technical replicates (See Figure 1C for coding). Red-bordered graphs indicate pairwise comparisons between biological samples and their direct technical replicate; graphs with black borders contain the remaining comparisons. Overall, a strong linear correlation is observed in pairwise comparisons (R^2^ = 0.95 ±0.01), in particular between biological and technical replicate pairs (R^2^ ≥ 0.96 ±0.01). Scale bars correspond to 100 peptides per protein group.

**Supplemental Figure 4. Copy number distribution of biological and technical replicates.** Log-log plots showing protein copy numbers from biological and technical replicates. Pairwise comparisons between biological samples and their direct technical replicate are indicated by red borders; black borders indicate the remaining comparisons. As in **Supplemental Figure 2**, pairwise comparisons show high correlations between individual samples (average R^2^ = 0.90 ±0.01), which is highest for biological and technical replicate pairs (R^2^ = 0.93 ±0.03).

**Supplemental Table 1. Origin and distribution of samples.** Colours correspond to those in Figure 1C. Samples that were run once are marked X, technically duplicated samples are marked XX.

**Supplemental Table 2. R commands for PCA analysis and plots.**
